# Physical Exercise Modulates L-DOPA-Regulated Molecular Pathways in the MPTP Mouse Model of Parkinson’s Disease

**DOI:** 10.1007/s12035-017-0775-0

**Published:** 2017-10-10

**Authors:** Cornelius J. H. M. Klemann, Helena Xicoy, Geert Poelmans, Bas R. Bloem, Gerard J. M. Martens, Jasper E. Visser

**Affiliations:** 1 Department of Molecular Animal Physiology, Donders Institute for Brain, Cognition and Behaviour, Radboud University, Nijmegen, The Netherlands; 2 Department of Cell Biology, Radboud University Medical Center, Nijmegen, The Netherlands; 3 Department of Human Genetics, Radboud University Medical Center, Nijmegen, The Netherlands; 4 Department of Neurology, Donders Institute for Brain, Cognition and Behaviour, Radboud University Medical Center, P.O. Box 9101, 6500 HB Nijmegen, The Netherlands; 5 Department of Neurology, Amphia Hospital, Breda, The Netherlands

**Keywords:** Parkinson’s disease, Physical exercise, MPTP, L-DOPA, (Non-)motor function, Molecular landscape

## Abstract

Parkinson’s disease (PD) is characterized by the degeneration of dopaminergic (DA) neurons in the substantia nigra pars compacta (SNpc), resulting in motor and non-motor dysfunction. Physical exercise improves these symptoms in PD patients. To explore the molecular mechanisms underlying the beneficial effects of physical exercise, we exposed 1-methyl-4-phenyl-1,2,3,6-tetrahydropyrimidine (MPTP)-treated mice to a four-week physical exercise regimen, and subsequently explored their motor performance and the transcriptome of multiple PD-linked brain areas. MPTP reduced the number of DA neurons in the SNpc, whereas physical exercise improved beam walking, rotarod performance, and motor behavior in the open field. Further, enrichment analyses of the RNA-sequencing data revealed that in the MPTP-treated mice physical exercise predominantly modulated signaling cascades that are regulated by the top upstream regulators L-DOPA, RICTOR, CREB1, or bicuculline/dalfampridine, associated with movement disorders, mitochondrial dysfunction, and epilepsy-related processes. To elucidate the molecular pathways underlying these cascades, we integrated the proteins encoded by the exercise-induced differentially expressed mRNAs for each of the upstream regulators into a molecular landscape, for multiple key brain areas. Most notable was the opposite effect of physical exercise compared to previously reported effects of L-DOPA on the expression of mRNAs in the SN and the ventromedial striatum that are involved in—among other processes—circadian rhythm and signaling involving DA, neuropeptides, and endocannabinoids. Altogether, our findings suggest that physical exercise can improve motor function in PD and may, at the same time, counteract L-DOPA-mediated molecular mechanisms. Further, we hypothesize that physical exercise has the potential to improve non-motor symptoms of PD, some of which may be the result of (chronic) L-DOPA use.

**Electronic supplementary material:**

The online version of this article (10.1007/s12035-017-0775-0) contains supplementary material, which is available to authorized users.

## Introduction

Parkinson’s disease (PD) is characterized by the degeneration of dopaminergic (DA) neurons in the substantia nigra pars compacta (SNpc). The clinical phenotype encompasses motor symptoms—including bradykinesia, rigidity, tremor, gait dysfunction, and postural instability—and non-motor symptoms such as sleeping disturbances, pain, or cognitive deficits that affect executive functions, attention, mood, and working memory [1–3]. Levodopa (L-DOPA), a precursor of DA, has been used since the 1960s to treat PD motor symptoms and is still considered the gold standard of therapy [4, 5]. In recent years, physical exercise—including intervention strategies such as aerobic exercise (e.g., treadmill exercise, cycling, or dancing) or strength training (e.g., using a modified fitness counts program or progressive resistance exercising)—has been reported to improve DA signaling [6, 7] and motor dysfunction [8–11], including bradykinesia [12, 13], rigidity [14], and tremor [12]. Physical exercise has also been reported to improve less dopamine-dependent symptoms involving postural control such as turning performance [6] and instability [15], as well as cognitive function [2, 16, 17] in PD patients. Although these beneficial clinical effects of exercise on PD symptoms are evident, the underlying molecular mechanisms are not well understood. A better understanding of these processes may ultimately lead to a more efficient treatment of these symptoms, through directly targeting the underlying pathways.

Systemic administration of 1-methyl-4-phenyl-1,2,3,6-tetrahydropyridine (MPTP) in mice results in the loss of nigrostriatal DA neurons, and is widely used to study the pathophysiological mechanisms underlying DA neuron degeneration in PD [18]. Moreover, similar to human PD, physical exercise improves motor behavior and reduces cognitive impairment in MPTP-treated mice [19–23]. To our knowledge, in this study, we elucidate for the first time the molecular pathways underlying the beneficial effects of exercise in PD, using the MPTP mouse model of PD.

## Methods

### Animals

Six-month-old male C57BL/6J mice were housed, five to a cage, with *ad libitum* access to food and water and at a constant 12/12 h light/dark cycle (lights on between 07:00 and 19:00 h). Room temperature was controlled at 21 °C and rooms were homogenously lighted by 60 LUX with controlled humidity. Following arrival, the mice were acclimatized to their new housing for 1 week, after which they were randomly assigned to one of four treatment groups: (1) saline-treated; (2) saline-treated with physical exercise; (3) MPTP-treated; and (4) MPTP-treated with physical exercise. MPTP-HCl (Sigma-Aldrich) dissolved in saline was administered via four intraperitoneal injections at 2 h intervals, amounting to a total administered dose of 70 mg/kg (free-base), which in a dose-response pilot experiment was found as the highest tolerable dose, with a survival rate of 75%. The intended concentration of 80 mg/kg, based on similar previous experiments [7, 24, 25], resulted in a higher toxicity and death rate that could not be justified. The control mice underwent the same protocol using saline injections. Mice were allowed to recover from the injections for 2 weeks. All saline-treated mice survived the protocol, *n* = 14 for group (1) and *n* = 14 for group (2), whereas, due to MPTP treatment, group (3) and group (4) eventually consisted of *n* = 10 and *n* = 13 mice, respectively.

### Physical Exercise

A forced chronic and aerobic physical exercise regimen was initiated 3 weeks following MPTP or saline treatment and was performed daily. Mice ran 30 min twice a day during a training period of 28 consecutive days in individual, horizontal lanes on a five-lane treadmill (Panlab Harvard Apparatus) at a speed of 20 cm/s (as used before in comparable experimental setups [7, 24, 26, 27]). Automated short air puffs were used to stimulate the mice to keep running when drifting too far to the back of the lane. All mice were able to perform the physical exercise without any noticeable problems. Mice assigned to the groups without physical exercise were placed in their housing cage in the same experimental room, adjacent to the treadmill.

### Behavioral Testing

Behavioral testing commenced 1 week before the physical exercise regimen started (week 0), and was repeated each week (similar to comparable experiments by others [7, 28–30]) during the exercise regimen (weeks 1–4): beam walk on the first, rotarod on the third, and open field on the fifth day of each week, in each case performed between 08:00 and 13:00 h. Prior to all behavioral tests, the animals of all four treatment groups were habituated to the experimental room for 1 h. Mice from different treatment groups were tested concurrently on the rotarod and in the open field.

#### Open Field

The mice were placed in a white plexiglass box (50×50×40cm) and video recorded from above for 30 min using EthoVision XT 7.0 software (Noldus Information Technology B.V., Wageningen, The Netherlands). Afterwards, the parameters “total walking distance”, “total movement time”, “mean velocity”, and “mean angular velocity” were calculated by the software, as described previously [31].

#### Rotarod

Mice were placed on the rotarod apparatus (IITC Inc., Woodland Hills, CA, USA) with a rod diameter of 32 mm and an increasing speed of 4 to 38 rpm in 300 s. Five mice were tested simultaneously on the rotarod and their latency to fall was measured (similar to [7, 30, 32]). On each testing day, each mouse performed one pre-trial and three trials, each with a maximum duration of 300 s and with a minimum of 1 h of rest between the trials. The pre-trial enabled the mice to habituate (again) to the rotarod and was not included in the results. For each testing day, the latency times of the three trials were averaged per mouse.

#### Beam Walk

We assessed the motor coordination and balance by measuring the ability of the mice to transverse a narrow beam [28, 33]. The mice were placed on a white plasticized iron rod (full length 80 cm, diameter 10 mm) suspended at 40 cm height and were trained to cross the beam to their home cage. Training of the mice occurred on the first day. During the training, the distance to cross was increased each time they successfully reached their cage, until they were able to reach their home cage over the full length of the beam. Each week, the mice were habituated again to the experimental setup by a pre-trial, which was followed by three trials in which the time was recorded how long it took for the mice to cross the full beam to reach their home cage; inter-trial interval was in all cases at least 1 h. For each testing day, the times of the three trials were averaged per mouse.

### Immunohistochemistry

We performed immunohistochemistry to establish TH protein expression [24, 34] in the DL, VM, SNpc, and VTA. Twenty-four hours following their last exercise training, mice were sacrificed by cervical dislocation, and brains were dissected and fixated in 4% paraformaldehyde in PBS solution for 3 h and subsequently cryoprotected by immersion in 30% sucrose for 24 h. After cryosectioning, DAB staining was performed on 20-μm-thick coronal slices, placed on gelatinized glass slides. For this, the sections were washed with PBS (3×10min), non-specific sites blocked with blocking buffer (2.5% normal donkey serum, 2.5% normal goat serum, 1% BSA, 1% glycine, 0.1% lysine, and 0.4% Triton X-100 in PBS) for 30 min and incubated with rabbit anti-tyrosine hydroxylase (TH, 1:1000; Pel-Freez Biologicals #P40101-0; lot no. 19335) for 16 h at 4 °C. This was followed by 1 h incubations with biotinylated goat-anti-rabbit (1:200; Jackson ImmunoResearch; 711-065-152; lot no.117858) and avidin-biotin-peroxidase complex (A and B 1:800; Vectastain Elite ABC kit, PK-6100 Standard), with PBS washing steps in between. To visualize antibody binding, the sections with SNpc and ventral tegmental areas (VTA) were incubated for 30 min, and those with dorsolateral striatum (DL) and ventromedial striatum (VM) areas were incubated for 20 min in a DAB/H_2_O_2_-solution potentiated by ammonium nickel sulfate. The sections were subsequently dehydrated and cover slipped. For each mouse, every sixth section throughout the DL, VM, SNpc, and VTA was included in the counting procedure, and for optimal comparison between groups, sections of different treatment groups were stained concurrently.

Images were captured by a Leica DM6000 B microscope. TH-positive (TH+) cells were counted in the sections of the SNpc (−2.54 to −3.88 mm to Bregma [35]) and VTA (−2.92 to −3.88 mm to Bregma [35]), using a 20× magnification. The number of TH+ cells in each section (both the left and right side) was counted by a blinded assessor and averaged over the total number of sections per animal. DA fiber density was estimated in the DL (1.18 to −0.10 mm to Bregma [35]) and VM (1.54 to 0.62 mm to Bregma [35]) by quantifying the optic density (OD) with FIJI [36], using a 5× magnification. In both areas, the OD per section was determined by averaging the OD of ten separate areas within the striatal matrix (i.e., in-between the striosomes). Subsequently, the OD was normalized by subtracting the OD of the corpus callosum (CC) or anterior commissure (AC) for the DL and VM respectively, in the same section, and all sections were averaged per animal.

### RNA Isolation and Sample Preparation

Twenty-four hours following the last physical exercise training, brains of 8–10 mice per group—that were sacrificed by cervical dislocation—were dissected, immediately frozen on dry ice, and stored at −80 °C until further preparation. Specific brain areas, i.e., prefrontal cortex (PFC), DL, VM, VTA, SN, and pedunculopontine nucleus (PPN), were then cryopunched based on the stereotaxic atlas of the mouse brain [35] from 200-μm-thick coronal slices, using punch needles with a diameter of 0.5 and 0.75 mm (see Online Resource 1 for the estimated punching locations per area). All specimens were kept at −20 °C during processing. For RNA isolation, punched samples were homogenized with a TissueLyser (Retsch GmbH) in 800 μL TRIzol reagent and RNA isolation was performed according to the manufacturer’s instructions (Invitrogen). Total RNA concentration was determined with a Nanodrop^TM^ ND-1000 spectrophotometer (Thermo Fisher Scientific Inc.), and RNA quality was visually assessed by 1% agarose gel electrophoresis. Genomic DNA was removed by treatment with DNase I in the presence of RNAsin (Thermo Fisher) in 5× FSB buffer and RNAse-free water. Subsequently, total RNA samples were stored at −80 °C until further use. For each treatment group and brain area, RNA samples of six mice were pooled for RNAseq analysis.

### RNA Sequencing and Data Processing

All RNA samples were subjected to RNA sequencing (RNAseq; HudsonAlpha Genomic Services Lab, Huntsville, AL) as performed before [37]. In short, total RNA concentration was estimated by Qubit 2.0 Fluorometer (Invitrogen, Carlsbad, CA, USA) and RNA integrity by using the Agilent 2100 Bioanalyzer (Applied Biosystems, Carlsbad, CA, USA). RNAseq libraries were formed from approximately 500 ng total RNA of each pooled sample, followed by poly(A) enrichment. RNAseq was performed using paired-end sequencing on Illumina HiSeqH2000 (Illumina, San Diego, CA, USA), at 50 base pairs, generating over 25 million paired reads per sample. Raw RNAseq FASTQ files were demultiplexed by bcl2fastq conversion software v1.8.3 (Illumina, Inc., San Diego, CA, USA) using default settings.

RNAseq data was analyzed using GeneSifter software (VizX Labs, Seattle, WA). RNAseq reads were mapped to the *Mus musculus* reference genome build 37.2, and for this, the reads were trimmed by 15 base pairs at the five-prime end. Subsequently, transcript abundance was calculated by estimating the reads per kilobase of exon per million mapped reads (RPKM), and normalization to the number of mapped reads was used for comparison of two mRNA sets. A *t* test was used for pairwise comparison and a likelihood ratio test to adjust for distribution probability.

### qPCR Validation

The RNAseq results were validated by comparing expression levels of at least eight mRNAs/genes per area with their expression as established by qPCR. These genes were chosen randomly, although there was one requirement, namely that genes from all three comparisons of interest, i.e., the comparisons to assess the effect of MPTP (group 3 vs. group 1), physical exercise (group 2 vs. group 1), and physical exercise in the MPTP model of PD (group 4 vs. group 3), should be included. RNA from the same samples used for the RNAseq pools was reverse-transcribed to cDNA with random primers using the RevertAid H Minus First Strand cDNA Synthesis Kit (Thermo Scientific, #K1632 lot no 00167909) according to the manufacturer’s protocol. Three-step qPCR (95 °C for 10 min, followed by 45 three-step cycles of 95 °C for 5 s, 65 °C for 10 s, and 72 °C for 20 s, and the generation of melting curves from 70 °C to 95 °C; Rotor-Gene 6000 Series, Corbett Life Science Pty. Ltd.) was performed using the 2× SensiFAST SYBR No-ROX mix (Bioline lot no SF582-313209) and primers designed with NCBI Primer-Blast (www.ncbi.nlm.nih.gov/tools/primerblast/) and synthesized at Sigma Life Sciences (The Netherlands) (For a complete overview of used primers, see Online Resource 10, Supp. Table 1). The housekeeping genes ACTB and YWHAZ were used as reference for normalization of gene expression. Based on the qPCR results, the minimum requirements to be included in the enrichment analysis—regarding fold change (FC) cut-off, maximum likelihood ratio value, and minimal RPKM value—were adjusted so that at least 90% of the expression changes could be validated by qPCR. As there was insufficient remaining RNA available to perform the complete qPCR validation for the PPN RNAseq data, the same cut-off values were used as for the other brain areas.

### Overlap of MPTP- and Exercise-Regulated Genes

To determine the direct effect of exercise on MPTP-regulated genes, we looked at the overlap between the genes regulated by MPTP (group 3 vs. group 1) and the genes regulated by exercise in the MPTP model (group 4 vs. group 3). To quantify this overlap, we used the hypergeometric distribution test [38]:

$$ p\left(x|\ n,M,N\right)=\frac{\left(\begin{array}{c}\hfill M\hfill \\ {}\hfill x\hfill \end{array}\right)\left(\begin{array}{c}\hfill N-M\hfill \\ {}\hfill n-x\hfill \end{array}\right)}{\left(\begin{array}{c}\hfill N\hfill \\ {}\hfill n\hfill \end{array}\right)} $$

and determined the chance of observing exactly *x* overlapping genes from a total of *n* differentially expressed genes by exercise in the MPTP model, with a total of *M* genes that were differentially expressed by MPTP and a total of *N* genes detected with RNAseq. The number of unique genes detected with RNAseq in each brain area (*N*), consists of genes detected in both comparisons (group 3 vs. group 1 and group 4 vs. group 3), irrespective of their FC or expression *p* value. Of note, for all comparisons only protein-coding genes were considered.

### Enrichment Analysis and Building of Molecular Landscapes

The Ingenuity pathway analysis software package (www.ingenuity.com) was used to identify enriched categories in the lists of differentially expressed protein-coding mRNAs in each of the brain areas [39]. Again, we focused on the three main comparisons of interest (see above)—i.e., the comparisons that assess the effect of MPTP, physical exercise, and physical exercise in the MPTP model of PD—in the six brain areas. Ingenuity assigns genes or rather their corresponding mRNAs/proteins to functional (sub)-categories, i.e., “canonical pathways” and “biofunctions”, with the latter including “diseases and disorders” and “molecular and cellular functions”. In addition, Ingenuity generates a list of “upstream regulators”, i.e., proteins or compounds that regulate multiple proteins/mRNAs from the input list. When possible, the program also calculates a *z* score that is based on the expression changes of the input mRNAs and that is a measure for the directionality of the upstream regulator, canonical pathway, or biofunction. A *z* score < −2 or > 2 is considered significant. For all analyses, only functional categories and upstream regulators with significant enrichment (i.e., Benjamini-Hochberg corrected *p* < 0.05) and containing at least two genes were taken into account.

Proteins/mRNAs regulated by the top upstream regulators were analyzed in more depth to identify their relation to physical exercise-induced processes in the MPTP model of PD (i.e., the comparison of group 4 with group 3). Guided by the results of the Ingenuity enrichment analyses, an extensive literature search was performed for the (putative) roles of all proteins encoded by the differentially expressed mRNAs as well as their functional interactions, using the UniProt Protein Knowledge Base (http://www.uniprot.org) and PubMed (http://www.ncbi.nlm.nih.gov/pubmed). Based on these findings and applying an approach similar to the one we used previously for genome-wide association and expression data [39–42], we then built molecular landscapes containing interacting proteins encoded by the mRNAs that are differentially expressed by physical exercise and are known to be regulated by the top regulators for each brain area. To complement these protein interaction cascades, we added a number of proteins that were not encoded by the differentially expressed mRNAs but that have been implicated in PD etiology through other lines of (genetic) evidence. In this respect, proteins encoded by familial PD candidate genes were included if they have at least one functional interaction with one or more other landscape proteins. Additional proteins were included when having at least two interactions with other landscape proteins. The molecular landscapes were drawn with Serif DrawPlus 4.0.

### Statistics

Statistical comparisons of values between multiple treatment groups were carried out using a two-way ANOVA. For behavioral test data, with data at multiple time points, a linear mixed model was applied using SPSS (IBM, version 23), with “week”, “physical exercise”, and “MPTP” as fixed factors to calculate the main effects of the training period, physical exercise, and the interaction between physical exercise and MPTP. The main effect of MPTP in the behavioral tests was assessed using a pairwise comparison of saline-treated and MPTP-treated mice before the start of the exercise regimen. For pairwise comparison, an *F* test was used to determine if the distributions of the compared two groups have the same variance. Based on the *F* test, a Student’s *t* test for equal or unequal variance was then used to evaluate the significance of the expression differences. For all comparisons, data are represented as mean with the standard error of the mean (SEM), and a *p* value < 0.05 was considered statistically significant.

The *p* values calculated with the hypergeometric distribution test were adjusted for multiple testing using the Bonferroni correction.

## Results

In this study, we assessed the effects of physical exercise in the MPTP-treated mouse model of PD at the behavioral and molecular levels.

### Physical Exercise Affects the Motor Function of MPTP-Treated Mice

**Fig. 1:**
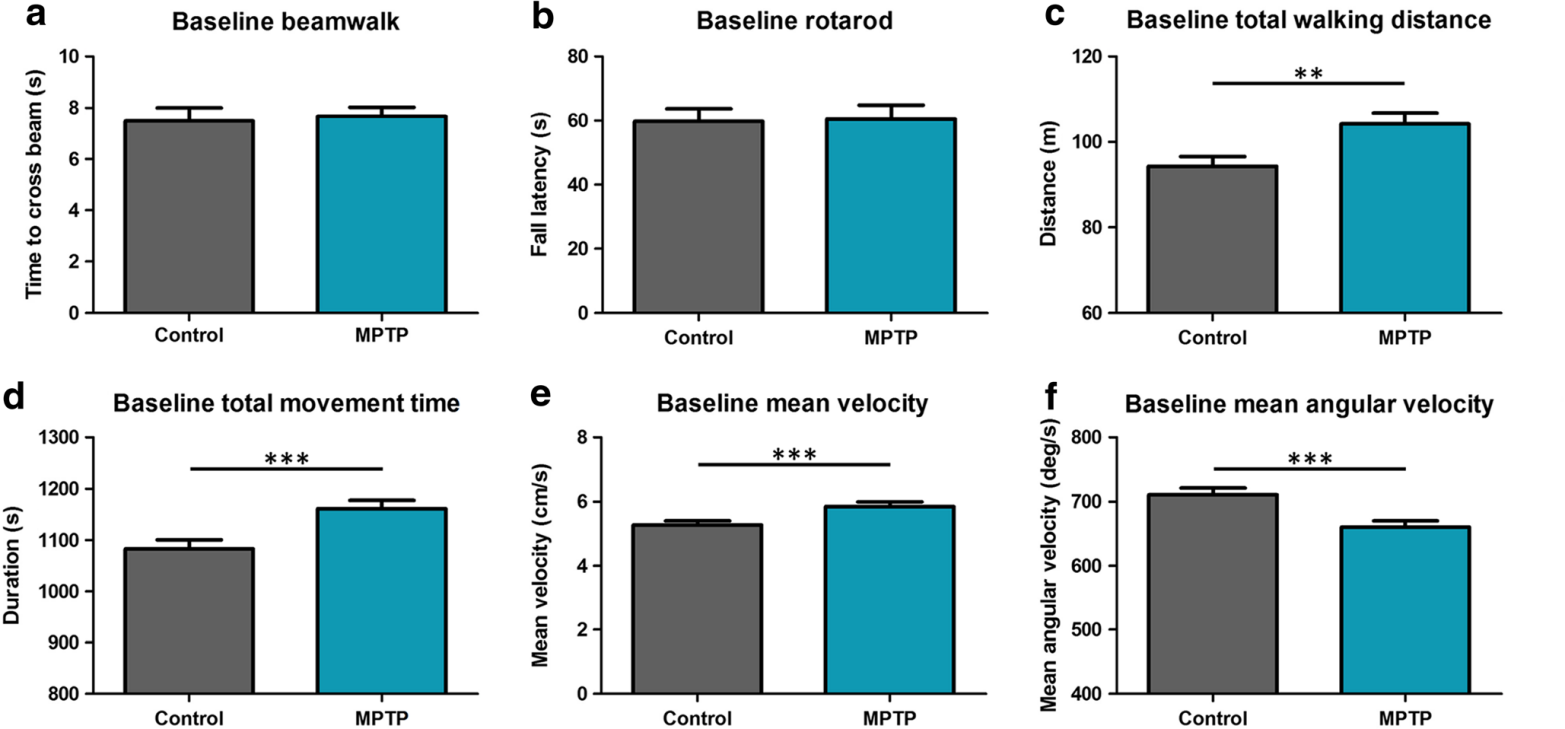
Effect of MPTP. Results of the behavioral tests in week 0 (set as the baseline (100%) for the effect of physical exercise, which is shown in Fig. 2). No effect of MPTP was shown for the beam walk (**a**), or rotarod (**b**) tests, but MPTP significantly affects the parameters in the open field (**c**–**f**). **p < 0.01; ***p < 0.005, mean + SEM, *n* = 28 for controls (saline-treated), and *n* = 23 for MPTP-treated mice

At baseline, i.e., following recovery from MPTP treatment but before the exercise regimen started, MPTP-treated mice showed an increased total walking distance (*p* < 0.01), total movement time (*p* < 0.005), and mean velocity (*p* < 0.005), and a decreased mean angular velocity (*p* < 0.005) in the open field compared to saline-treated control mice. In contrast, their performance on rotarod and beam walk tests was not significantly different from controls (Fig. 1).

**Fig. 2:**
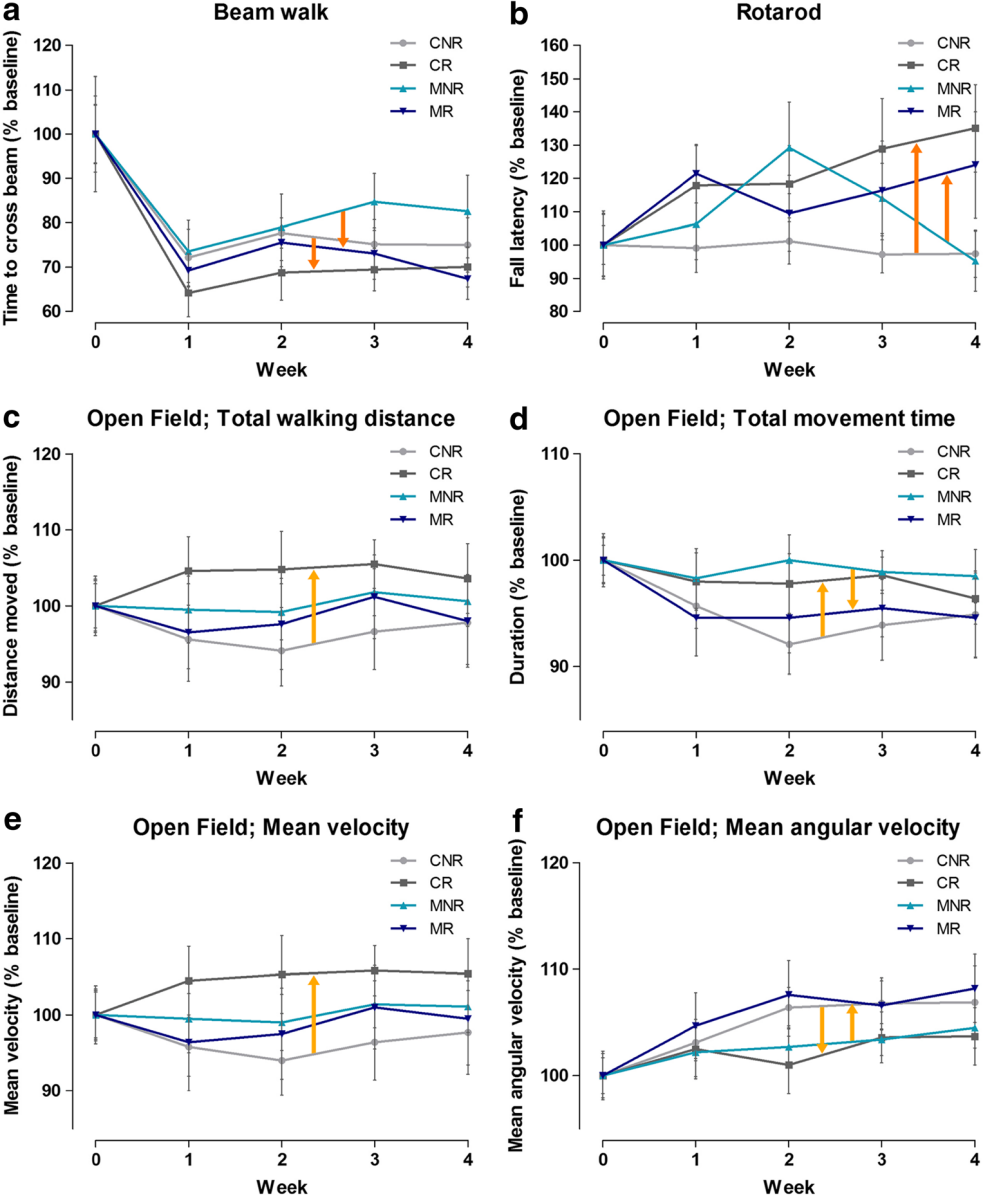
Effect of physical exercise and interaction with MPTP compared to the baseline (week 0); results of the behavioral tests in week 0–4. Measurements in week 1–4 were normalized to week 0 (100%). Dark-orange arrows indicate the main effect of physical exercise (**a**, **b**), and light orange arrows show the effect of the interaction between physical exercise and MPTP treatment (**c**–**f**) mean ± SEM *n* = 14 for both CNR and CR, *n* = 10 for MNR and *n* = 13 for MR. CNR, control not running; *CR* control running, *MNR* MPTP-treated but not running, *MR* MPTP-treated and running

In Fig. 2, the effects of physical exercise during the course of the training period relative to baseline are shown for each of the four treatment groups. The beam walk task showed a clear training effect over time in all groups (main effect of “week” *p* < 0.001), and the test performance was improved by physical exercise in both the MPTP-treated and saline-treated mice, without significant differences between the groups (main effect of physical exercise *p* < 0.05) and no significant interaction between physical exercise and MPTP treatment (Fig. 2a). Rotarod performance was also significantly improved by physical exercise (*p* < 0.01), but no improvement over time or interaction with MPTP treatment was found (Fig. 2b). Of the tested parameters in the open field (total walking distance, total movement time, mean velocity, and mean angular velocity), the mean angular velocity was increased (*p* < 0.001), and the total movement time showed a decreasing trend (*p* = 0.051) for all treatment groups over time during the exercise regimen (i.e., main effect of “week”). There was no significant (main) effect of physical exercise on any of the four tested open field parameters, only a trend towards a higher “mean velocity” (*p* = 0.082). However, for all four open field parameters, significant interactions between physical exercise and MPTP treatment were found (*p* < 0.05). Physical exercise *increased* the walking distance and mean velocity of saline-treated mice, but *not* of MPTP-treated mice. Moreover, physical exercise *increased* the total movement time of saline-treated mice and *decreased* that of MPTP-treated mice. This opposite effect was also observed for mean angular velocity, i.e., a *decrease* by physical exercise in saline-treated mice and an *increase* in MPTP-treated mice (Fig. 2c–f).

### TH Depletion in the SNpc and Striatum Following MPTP Treatment

**Fig. 3:**
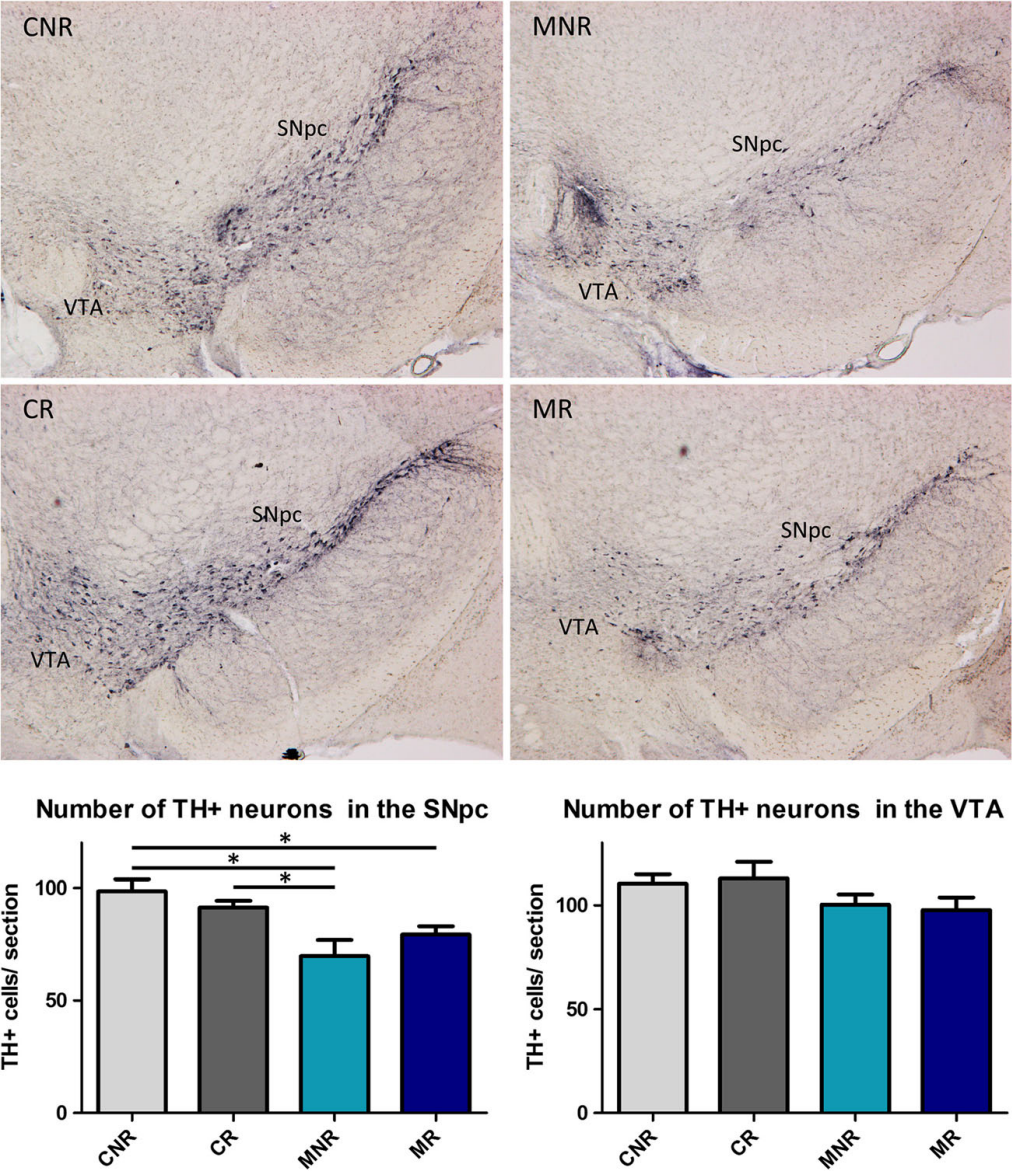
TH+ neurons in the SNpc and VTA. The upper panel shows a representative picture for each of the four treatment groups and the lower panel shows the average number of TH+ neurons in the SNpc and the VTA per treatment group. *p < 0.05, mean + SEM, *n* = 5 for CNR and MR and *n* = 4 for CR, and MNR for both brain areas. *CNR* control not running, *CR* control running, *MNR* MPTP-treated but not running, *MR* MPTP-treated and running, *SNpc* substantia nigra pars compacta, *VTA* ventral tegmental area

The number of DA neurons in the SNpc and VTA of each treatment group, as well as an estimate of DA fiber density in striatal target areas (DL and VM, respectively) was determined by immunohistochemistry for TH—the rate-limiting enzyme in DA synthesis. These measures were primarily taken to confirm and estimate the degree of neuronal loss due to MPTP treatment, but they may also provide some insight into whether exercise could affect these structural changes. MPTP significantly reduced the number of TH+ cells in the SNpc (*p* < 0.005), but not in the VTA. Pairwise comparison between the treatment groups revealed that the number of TH+ cells in the SNpc of MPTP-treated mice *without* and *with* physical exercise was reduced by 29 and 20%, respectively, compared to the saline-treated group *without* exercise (both *p* < 0.05; Fig. 3). There was no significant effect of physical exercise on the number of TH+ cells in either the SNpc or the VTA, and no interaction between MPTP and physical exercise.

In Online Resource 2, the relative OD of TH+ fibers in the DL, the primary striatal target area of the SNpc, is shown. The OD of TH+ fibers was reduced by MPTP (*p* < 0.05), without a main effect of physical exercise or an interaction between MPTP and physical exercise. Pairwise comparison showed that MPTP decreased the density of TH+ fibers in MPTP-treated mice *without* exercise by 33% (*p* < 0.005) compared to saline-treated mice *without* physical exercise. There was a trend towards an increased TH+ OD by physical exercise in MPTP-treated mice, but this increase was not significant.

Online Resource 3 shows the OD of TH+ fibers in the VM, the primary striatal target area of the VTA. Although all treatment groups (physical exercise, MPTP, and MPTP + physical exercise) showed a reduced OD of TH+ fibers, no significant effects of MPTP, physical exercise, or their interaction were found.

### qPCR Validation of the RNAseq Data

The RNAseq data were obtained from pooled samples, and in order to validate these data, the mRNA expression levels in each of the investigated brain areas were determined in individual samples by qPCR. The results of the qPCR experiments (Online Resource 4) led us to adopt the following requirements for the inclusion of differentially expressed protein-coding mRNAs in the subsequent analyses: FC > 1.2, likelihood ratio < 0.05, RPKM > 5.

### A Direct Effect of Physical Exercise on MPTP-Regulated Genes

The overlap between the protein-coding mRNAs that are differentially expressed due to MPTP alone and due to exercise in MPTP-treated mice is represented in Online Resource 5. In all brain areas, the probability of this overlap was calculated by using the hypergeometric distribution test, which showed that for all areas, the overlap is greater than would be expected based on random gene selection (*p* < 0.05). Further, in all areas, 82–99% of the overlapping mRNAs are regulated in opposite directions by MPTP and exercise. Enrichment analyses of mRNAs that overlap but are regulated in opposite directions are summarized in Online Resource 10, Supp. Table 2. The VTA and PFC show the most significant results, and are also the brain areas with the biggest absolute and relative overlap (i.e., the overlap in number and proportion of mRNAs). The analysis of the VTA displays a *downregulation* of the top regulator “inosine”, whereas the PFC and to a lesser extent also the DL show an *increase* in effect of dalfampridine and bicuculline.

### RNAseq Data Analysis: Enriched Regulators, Pathways, and Biofunctions

Enrichment analysis of the differentially expressed mRNAs was performed for each of the brain areas examined to investigate the effects of MPTP (i.e., comparing the MPTP-treated group *without* exercise to the saline-treated mice *without* exercise), physical exercise (i.e., comparing saline-treated mice *with* exercise to saline-treated mice *without* exercise), and the effects of physical exercise in MPTP-treated mice (i.e., comparing the MPTP-treated mice *with* exercise to MPTP-treated mice *without* exercise). In Tables 1, 2, and 3, a short overview of the main effects—the top regulator(s), canonical pathway(s), and biofunction(s)—of MPTP, physical exercise, and physical exercise in MPTP-treated mice is provided for each brain area separately. A more elaborate overview of these enrichment analyses per brain area can be found in Online Resource 10, Supp. Tables 3–8.

**Table 1:**
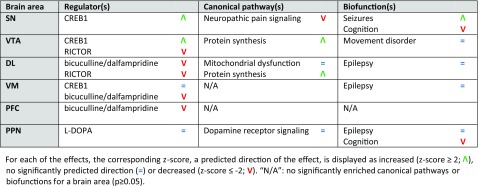
Main effects of MPTP (MPTP-treated mice *without* physical exercise vs. saline-treated mice *without* physical exercise) per brain area

**Table 2:**
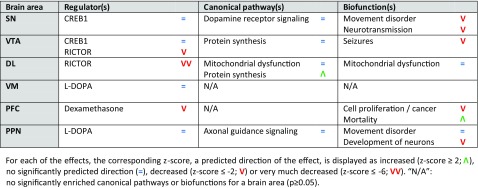
Main effects of physical exercise (saline-treated mice *with* physical exercise vs. saline-treated mice *without* physical exercise) per brain area

**Table 3:**
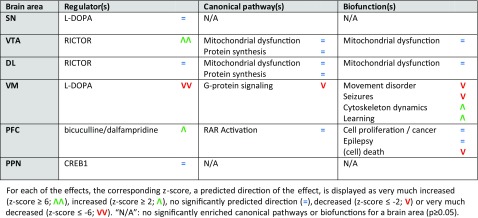
Main effects of physical exercise in MPTP-treated mice (MPTP-treated mice *with* physical exercise vs. MPTP-treated mice *without* physical exercise) per brain area

In all brain areas examined, MPTP treatment affected a set of mRNAs that is involved in epilepsy, which is reflected by the presence of the epilepsy-regulating transcription factor CREB1, the convulsants bicuculline and dalfampridine, and the biofunction “epilepsy”. Other regulators and related functional themes enriched within the mRNAs affected by MPTP are RICTOR and its regulation of ribosomal and mitochondrial proteins, as well as L-DOPA and DA receptor signaling (Table 1). Of note, in line with the MPTP-mediated decrease in TH expression in the DL and VM (not significant; see above), L-DOPA is a significant upstream regulator in both the DL (*p* = 2.85E-06; *z* = −2.635) and the VM (*p* = 9.97E-04; *z* = −1.234), but was not among the top 10 upstream regulators and is therefore not included in the (Supplementary) Tables.

Furthermore, in the various brain areas examined, physical exercise affected sets of mRNAs that are regulated by the upstream regulators CREB1, RICTOR, L-DOPA, and dexamethasone. These regulators overlap to some extent with the upstream regulators for the MPTP-regulated mRNAs as mentioned above. However, the top canonical pathways and biofunctions due to physical exercise are not epilepsy-related, but rather associated with “mitochondrial dysfunction” and “movement disorder” (Table 2).

The top regulators of the mRNAs differentially expressed due to physical exercise in MPTP-treated mice are L-DOPA, RICTOR, bicuculline/dalfampridine, and CREB1. The top canonical pathways and biofunctions enriched in exercised MPTP-treated mice are “mitochondrial dysfunction” and “protein synthesis” in the VTA and DL, “G-protein signaling”, “movement disorder”, “seizures and cytoskeleton dynamics” in the VM and are related to (cell) death in the PFC (Table 3).

Of note, the predicted direction of effect of the top regulators RICTOR and L-DOPA is changed in the VTA, DL, and VM of exercised MPTP-treated mice compared to exercised saline-treated mice. More specifically, the predicted direction of effect of RICTOR is (strongly) *decreased* in the VTA and DL after exercise in saline-treated mice, but is strongly *increased* and has no significant predicted direction in the VTA and DL of exercised MPTP-treated mice, respectively. Further, L-DOPA shows a strongly *decreased* predicted direction of effect in the VM of exercised MPTP-treated mice, whereas this direction of effect was *absent* after exercise alone.

### The Main Molecular Pathways Regulated by Physical Exercise

To elucidate the main molecular pathways regulated by physical exercise in MPTP-treated mice, the mRNA sets regulated by the top upstream regulators L-DOPA (in the SN and VM, Online Resource 10, Supp. Tables 9 and 10), RICTOR (in the DL and VTA, Online Resource 10, Supp. Tables 11 and 12), bicuculline/dalfampridine (in the PFC, Online Resource 10, Supp. Table 13), and CREB1 (in the PPN, Online Resource 10, Supp. Table 14) were studied in greater detail and used to build molecular landscapes for each top upstream regulator in the various brain areas. Here, we provide a short description of each of these molecular landscapes. In Online Resource 10, all landscapes are described in full detail. Of note, in the PPN, L-DOPA is the top upstream regulator following physical exercise or MPTP treatment, but L-DOPA (*p* = 1.97E-02; *z* score = −1.964), although significant, was not among the top 10 upstream regulators following physical exercise in MPTP-treated mice.

**Fig. 4:**
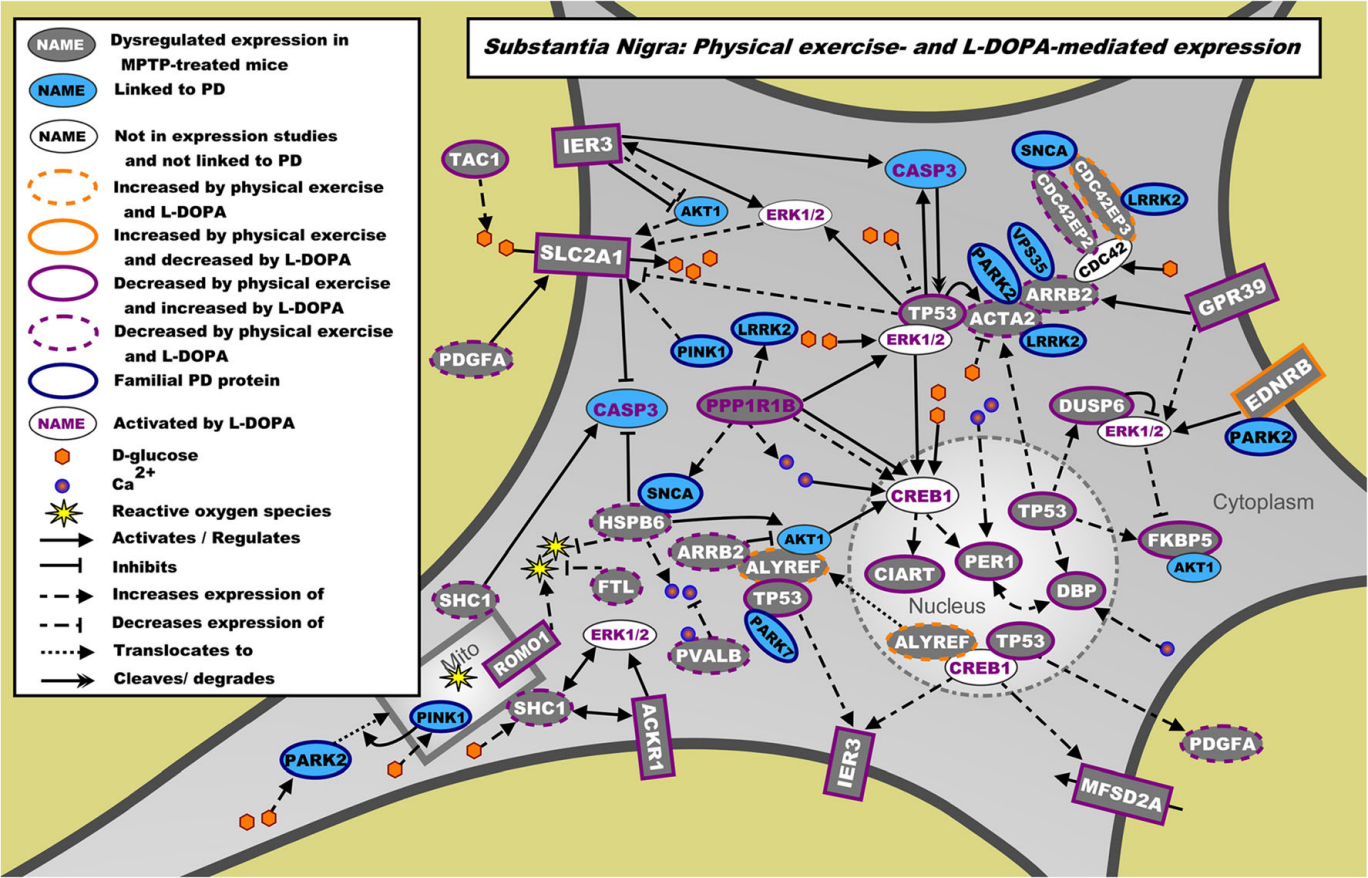
Landscape of proteins encoded by the mRNAs regulated by physical exercise and the upstream regulator L-DOPA in the SN. mRNAs differentially expressed in the SN due to physical exercise in MPTP-treated mice are shown in gray. Blue proteins are additional genes/proteins that are associated with PD through genetic and/or expression studies, whereas white proteins have no known link with PD. The direction of effect of physical exercise (measured) and L-DOPA (from literature) on the expression of these mRNAs is depicted through colored borders. L-DOPA-activated proteins are shown with purple writing for the protein name, and familial PD proteins are shown with a blue border

**Fig. 5:**
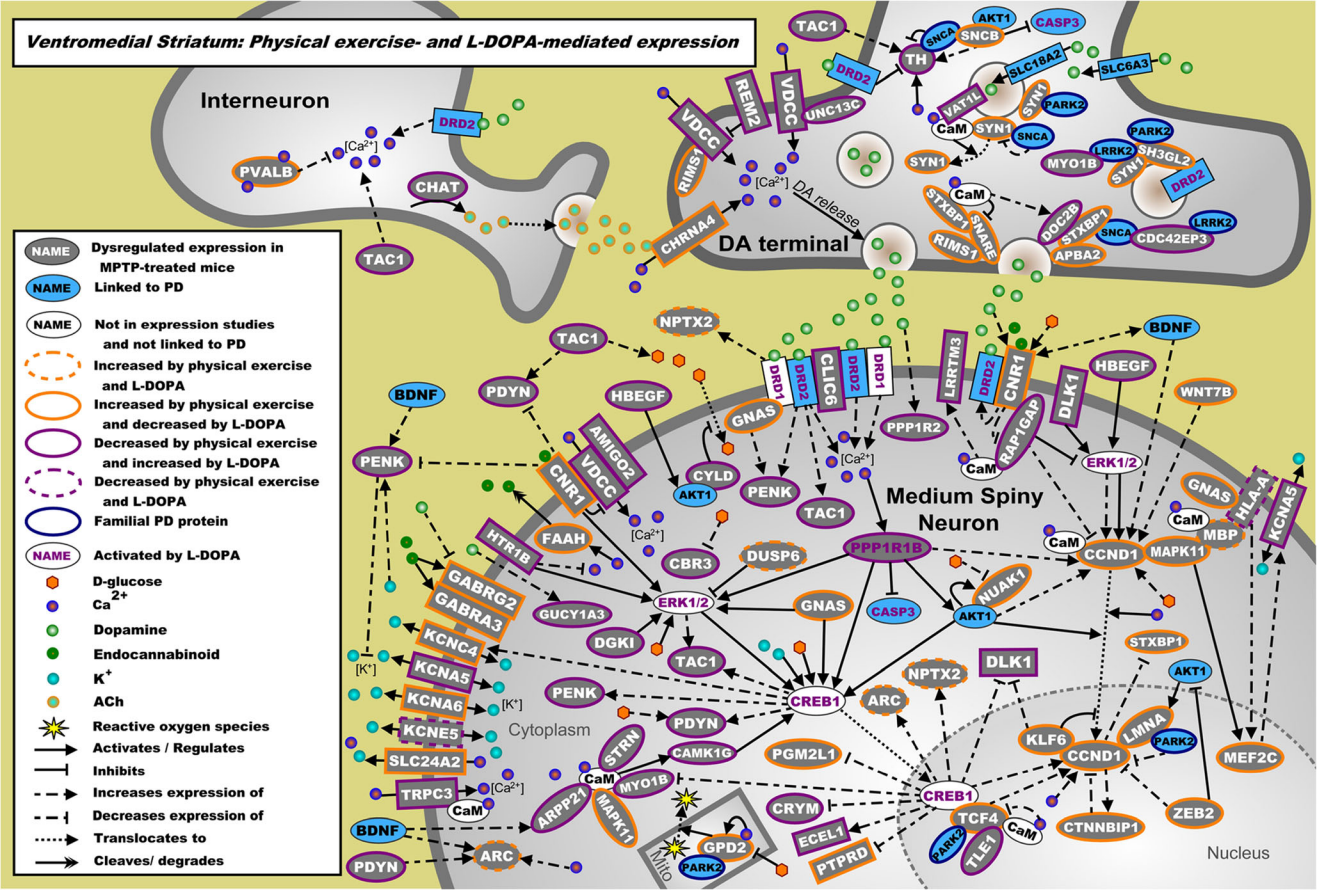
Landscape of proteins encoded by the mRNAs regulated by physical exercise and the upstream regulator L-DOPA in the VM. mRNAs differentially expressed in the VM due to physical exercise in MPTP-treated mice are shown in gray. Blue proteins are additional genes/proteins that are associated with PD through genetic and/or expression studies, whereas white proteins have no known link with PD. The direction of effect of physical exercise (measured) and L-DOPA (from literature) on the expression of these mRNAs is depicted through colored borders. L-DOPA-activated proteins are shown with purple writing for the protein name, and familial PD proteins are shown with a blue border

The molecular landscapes of interacting proteins encoded by the L-DOPA-regulated mRNAs that are differentially expressed in the SN and the VM due to physical exercise in MPTP-treated mice, are shown in Figs. 4 and 5, respectively. In the SN landscape, G-coupled receptor signaling (involving the proteins ARRB2 and GRP39), glucose uptake and signaling (SLC2A1), DA signaling (PPP1R1B), and reactive oxygen species (ROS) regulation (HSPB6, FTL, ROMO1) converge on the activation of ERK1/2 (ACKR1, EDNRB, GPR39, IER3, TP53), apoptotic pathways (CASP3, TP53), CREB1, and circadian clock regulation (PER1, DBP, CIART) (Fig. 4). In the VM landscape, the main molecular pathways are (interneuron-mediated) DA release (involving the proteins CHAT, DOC2B, SYN1, and TH) and signaling (DRD2, PPP1R1B), cannabinoid signaling (CNR1, FAAH), and neuropeptide signaling (PDYN, PENK, TAC1) that subsequently regulate/activate ERK1/2, CREB1, and CCND1 signaling. The latter is a cell cycle regulator that may also be involved in synaptic plasticity and learning [43] (Fig. 5). Of note, almost all proteins in this landscape are regulated by physical exercise and L-DOPA in opposite directions.

The RICTOR-regulated mRNAs that are differentially expressed in the DL and VTA due to physical exercise in the MPTP-treated mice encode proteins that are specifically involved in three cellular systems: the complex I-V of the electron transport chain, the 40S and 60S ribosomal subunits, and the proteasome (see Online Resource 10, Supp. Tables 11 and 12). These are complexes that regulate cellular energy, protein translation, and protein degradation, respectively (Online Resources 6 and 7). Of note, physical exercise and RICTOR have an opposite effect on the expression of all differentially expressed mRNAs in the mitochondrial electron transport chain in the DL, whereas physical exercise and RICTOR exert the same direction of effect (i.e., a decreasing effect) on the expression of electron transport chain mRNAs in the VTA.

In the PFC, 8 out of 9 mRNAs differentially expressed due to physical exercise in MPTP-treated mice and regulated by bicuculline/dalfampridine have been linked to epilepsy (Online Resource 10, Supp. Table 13). Immediate-early gene activation is one of the main processes regulated by these mRNAs e.g., via the early response genes/proteins FOS, FOSB, and NR4A1, which in turn are regulated by insulin and low-density lipoprotein. In Online Resource 8, an overview of the interactions of the proteins encoded by these mRNAs and their regulation by bicuculline/dalfampridine and physical exercise is shown in a molecular landscape.

In the PPN, the proteins encoded by the mRNAs that were differentially expressed due to physical exercise in MPTP-treated mice and regulated by CREB1 have only a limited number of interactions in the built landscape (Online Resource 9). Nevertheless, a few functional themes such as vascular remodeling, neuropeptide signaling, lipid metabolism, epilepsy/immediate-early gene regulation, and calcium signaling were identified, with CREB1 as their central regulator (Online Resource 10, Supp. Table 14).

## Discussion

This study aimed to explore the molecular mechanisms underlying the beneficial effects of physical exercise on motor functioning in the MPTP-treated mouse model of PD. After validation of the model, through demonstrating significant nigral neuronal loss following MPTP treatment, the effects of a four-week physical exercise regimen on motor performance, and the accompanying molecular changes in multiple brain areas were assessed using behavioral tests and RNAseq analysis, respectively. The behavioral tests showed that physical exercise improved beam walk and rotarod performance in both MPTP-treated and control mice, but had a different and often opposite effect on the four tested open field parameters in these groups. Our RNAseq findings demonstrated that physical exercise in MPTP-treated mice mainly affects the expression of mRNAs involved in L-DOPA-mediated pathways in the SN and VM that regulate DA signaling, RICTOR-mediated pathways in the VTA and DL involved in energy metabolism and cellular stress [44, 45], and bicuculline/dalfampridine-mediated pathways in the PFC and CREB1-mediated pathways in the PPN that are both a measure of neuronal activity [46, 47]. To further elucidate the specific molecular mechanisms underlying the effects of physical exercise in MPTP-treated mice, the differentially expressed mRNAs regulated by these top regulators were integrated into molecular landscapes, depicting the main biological processes and signaling cascades affected.

Our animal model was validated by demonstrating a significant nigral DA neuronal loss following MPTP treatment. The observed moderate neuronal loss in the midbrain due to MPTP treatment, i.e., a 29% reduction of TH-positive neurons in the SNpc without a statistical significant loss in the VTA, is in keeping with earlier studies using a similar MPTP treatment regimen in 5-month-old mice showing 33% loss in the SNpc and no significant loss in the VTA [48]. Other studies, on 8–10-week-old mice, have reported a neuronal loss of 29–45% [49, 50], but also of more than 50% loss in the SNpc [7, 24, 51]. Differences in level of neurodegeneration [52] and molecular effects [39] due to MPTP toxicity may be explained by MPTP dosing, age of the mice, and the duration between MPTP injection and sacrifice [48, 52]. We used aged (6-month-old) mice to better model age-dependent processes such as regulation of anti-oxidants [53], neuroplasticity, neurogenesis [54, 55], and the immune response in PD [56, 57]. To assess how exercise may boost any neuroplastic mechanisms of the injured basal ganglia, the physical exercise regime was performed within the recovery phase of striatal DA levels as reported in younger MPTP-treated mice [58], but after the acute neurotoxic (molecular) effects of MPTP [39, 59]. We did not find a significant effect of physical exercise on the number of surviving DA neurons, but noted a trend towards an increased number of TH-positive neurons in the SNpc and an increased TH-positive fiber density in the DL and VM in MPTP-treated mice with physical exercise compared to MPTP-treated mice without exercise. From previous studies, it remains unclear whether physical exercise can protect against cellular loss in the MPTP-mouse model. Preservation of SNpc neurons by physical exercise has been described before [27, 51], but the findings were inconsistent [7, 22].

Regarding motor function, forced exercise has more effect than voluntary exercise in both PD patients [60] and mice [61], and it activates the same brain areas as anti-PD medication does [62]. In this study, the mice were able to perform the physical exercise without any noticeable problems, suggesting that their physical exercise regimen is comparable to the forced moderate aerobic exercise that has been shown to improve both motor and non-motor functions in PD patients [11, 60, 63, 64]. MPTP treatment alone resulted in an increased activity in the open field, as reported before [48, 52, 65–67], but did not affect the performance on beam walk and rotarod. It should be noted that the training effect on the beam walk as seen in all four treatment groups, especially in week 1 compared to week 0, may implicate the necessity for more extensive training of the mice before the beam walk task in week 0. Further, the effects of exercise on the motor performance included an improvement on the beam walk and rotarod in both saline and MPTP-treated groups. However, the effects of physical exercise on the open field parameters in saline-treated mice was either absent or opposite in MPTP-treated animals. These findings suggest that some effects of physical exercise may be dependent on the “disease-state” (i.e., saline- or MPTP-treated). It could be argued, however, that the lack of effect of physical exercise in MPTP-treated mice on total walking distance and mean velocity (Fig. 2) may be due to their MPTP-induced hyperactivity (Fig. 1) that could have limited a further increase in motor performance due to physical exercise. This hyperactivity has been observed more often following MPTP treatment [48, 65, 66, 68–70] and may result from compensatory effects induced by e.g., brain areas of the mesolimbic pathway (see also below). Furthermore, the opposite effect of exercise on total movement time and mean angular velocity in MPTP-treated mice (Fig. 2) compared to the effect of MPTP alone (Fig. 1) suggests that physical exercise counteracts the effect of MPTP. This finding could have important translational value as axial symptoms in PD—such as hypokinetic rigidity which is reflected by reduced angular velocity [71–73]—are notoriously more difficult to treat by medication than appendicular symptoms.

The RNAseq analysis showed that the level of overlap between MPTP-regulated genes and physical exercise-regulated genes differed between the brain areas studied and was particularly high in the PFC and VTA. These data suggest that in the PFC and VTA, physical exercise influences the processes affected by MPTP more directly than in the other areas in which more indirect mechanisms may prevail. Nevertheless, in all brain areas examined, the majority of overlapping genes (82–99%) were regulated in opposite directions by physical exercise compared to MPTP, suggesting counteracting effects of physical exercise on MPTP-regulated mechanisms. For example, the enrichment analysis of the overlapping genes in the PFC and DL (see Online Resource 10, Supp. Table 2) shows a predicted activation of the top regulators dalfampridine, bicuculline, and CREB1—indicative for neuronal activation [46, 47]—whereas these are inactivated by MPTP.

**Fig. 6:**
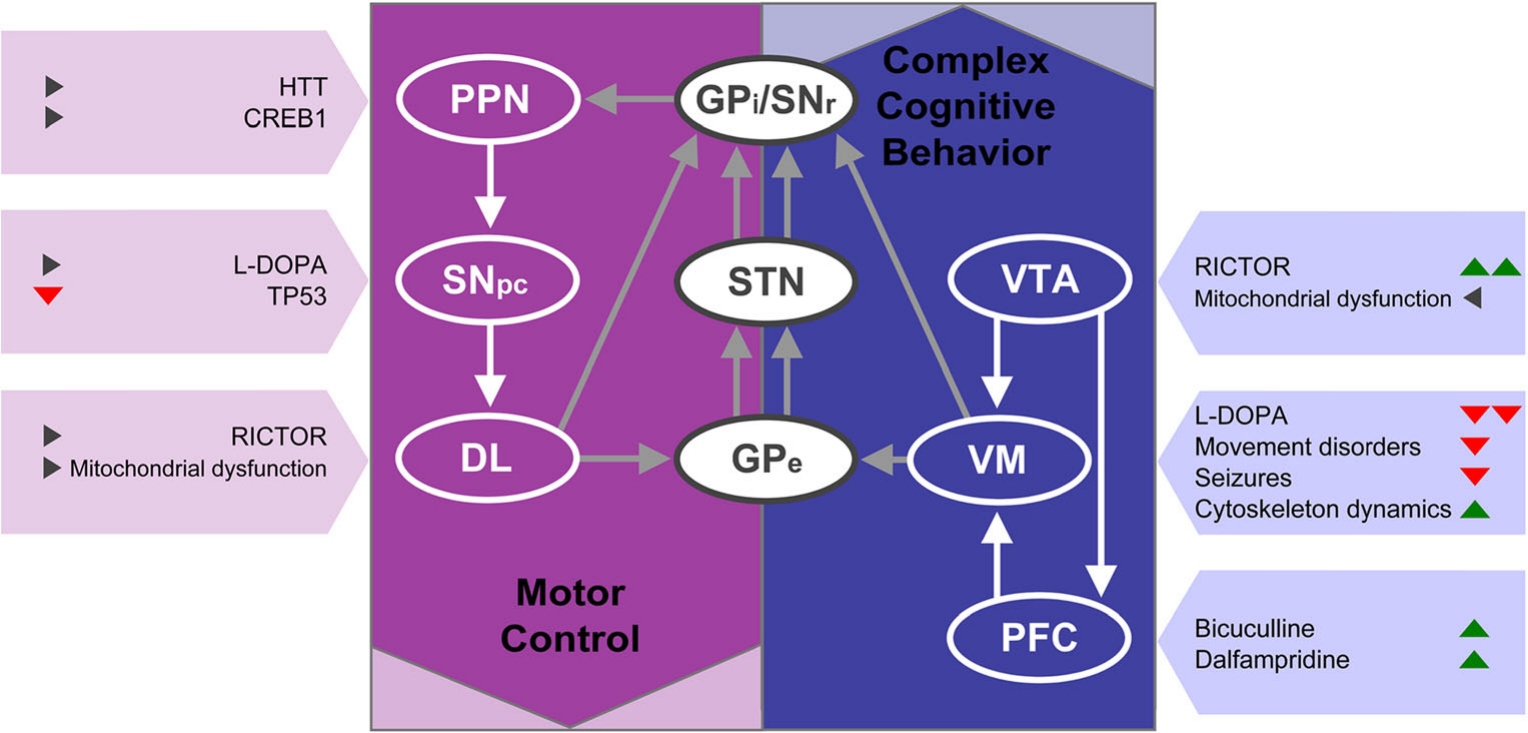
Overview of the brain areas analyzed, and the top upstream regulators, and processes per area. The brain areas are shown in a simplified model of the basal ganglia circuitry. Green, red, and gray triangles depict positive (> 2), negative (< −2) or non-significant *z* scores, respectively, from the enrichment analyses of the physical exercise-regulated mRNAs in MPTP-treated mice. *DL* dorsolateral striatum, *GPe* globus pallidus external, *GPi* globus pallidus internal, *PFC* prefrontal cortex, *PPN* pedunculopontine nucleus, *SNpc* substantia nigra pars compacta, *SNr* substantia nigra reticularis, *STN* subthalamic nucleus, *VM* ventromedial striatum, *VTA* ventral tegmental area

The roles of the PD-related brain areas examined in this study can be summarized in a simplified basal ganglia circuitry model, wherein PPN, SN, and DL are mainly involved in motor control, and the VTA, VM, and PFC contribute particularly to the regulation of (complex) behavior and cognition (Fig. 6) [74–79]. The top regulators—and to a lesser extent also the canonical pathways and biofunctions—regulated by physical exercise in the cognition-associated brain areas of MPTP-treated mice, showed highly significant predicted directions of effect, whereas these effects were less prominent in the motor-related areas. This implicates that, although physical exercise is able to improve motor function (as supported by the behavioral tests), it may also have strong effects on cognition and behavior. This is interesting from a therapeutic point of view, because non-motor symptoms in PD patients—including cognitive impairment, depression, pain, and sleep disorders—are usually less responsive to dopamine replacement therapy and therefore treatment options are limited [80–82]. It remains unclear, however, to what extent these motor and non-motor features of PD have truly discernible neuroanatomical or molecular substrates, as effects of changes in mRNA expression in the “behavioral areas” VTA, VM, and PFC on motor function of our animals cannot be excluded. For example, a recent paper reported that VTA-specific knockout of RICTOR in mice may affect cognition and mood, but also results in hyperactivity in the open field [83]. In addition, it has been suggested that during exercise, the mesolimbic pathway (including the VTA and VM) may provide a compensatory functional activation of the motor loop [84]. Furthermore, whereas L-DOPA is known to improve DL-mediated motor symptoms, it may impair VM function in PD patients [85, 86]. Therefore, exercise may counteract L-DOPA-mediated pathways in the VM and as such improve VM functionality, which could in turn result in increased compensatory motor-loop activation. Finally, inhibition of GABAergic interneurons in the PFC by bicuculline increases the release of DA in the DL through the glutamatergic corticostriatal pathway [87–89] and may increase the locomotor activity of mice [88, 90]. This is in line with the reduced TH expression we observed in the DL and the inactivation of bicuculline/dalfampridine-regulated pathways in the PFC following MPTP treatment, as predicted on the basis of the RNAseq analysis. Moreover, we found no significantly reduced TH expression in the DL of exercised MPTP-treated mice that, in contrast to MPTP-treated mice without exercise, showed a predicted activation of the bicucullin/dalfampridine-regulated pathways in the PFC.

Almost five decades after its introduction [4], the DA precursor L-DOPA is still the gold standard for symptomatic treatment to alleviate the motor symptoms of PD [5]. It should be noted, however, that chronic high-dose L-DOPA use is associated with complications such as dyskinesias [91–93]. Moreover, the effects of L-DOPA on non-motor symptoms in PD are even less predictable and L-DOPA use may even lead to deterioration of these symptoms, e.g., impaired reversal learning or motor sequence learning deficits [94–101]. It has been suggested that these adverse cognitive effects of L-DOPA may be due to a higher L-DOPA demand in the motor systems compared to cognitive areas, resulting in a relative L-DOPA overdose in cognitive areas [102–104] e.g., the VM (see also above). Therefore, novel “add-on” treatments that can enable low-dose L-DOPA use and/or reduce the adverse effects of (long-term) L-DOPA use are desirable. In this respect, our study suggests that physical exercise is an attractive add-on treatment for PD, and that exercise combined with L-DOPA treatment may be more beneficial than treatment of PD patients with L-DOPA alone [9, 105]. Other findings that support this hypothesis include the reports indicating that physical exercise not only improves the motor symptoms of PD patients [8, 9], but also L-DOPA-induced dyskinesias in PD patients [106] and animal models [107], and cognitive function in PD patients [2, 16, 17]. In this light, it is of note that L-DOPA use may result in alpha-synuclein-induced neuroinflammation [108] that very recently has been shown to be reduced by physical exercise [109–111]. Although the major pathways regulated in our study are not directly related to inflammation, L-DOPA-mediated pathways may affect alpha-synuclein regulation [108], and the RICTOR-regulated pathways may improve mitochondrial function and protein turn-over, i.e., processes that have been suggested to reduce alpha-synuclein-induced neuroinflammation [111].

Considering the above, it is worth noting that our landscapes revealed that physical exercise and L-DOPA regulate similar pathways in the SN and VM—often in an opposite direction—and that most of these pathways have been linked to sleeping problems (SN) and cognitive and/or motor dysfunctioning (VM) in PD. For example, the expression of clock proteins was affected by physical exercise and L-DOPA in the SN, a brain region known to be involved in the regulation of REM sleep [112, 113] and causing circadian rhythm irregularities when damaged by MPTP [114, 115]. Further, the use of L-DOPA can disturb REM sleep [116] and result in a delayed sleep onset in PD patients, which suggests an uncoupling of sleep and circadian regulation [117]. On the other hand, physical exercise can improve circadian rhythm regulation [118–120] and may therefore serve as a complementary therapy to strengthen circadian function in PD, as suggested earlier [121].

In the VM, both physical exercise and L-DOPA regulate DA, neuropeptide, and endocannabinoid signaling, but in opposite directions. L-DOPA treatment results in sustained DA signaling in the striatum and can disrupt DA and (endo)cannabinoid receptor crosstalk [122, 123]. In contrast, physical exercise may rebalance DA signaling after sustained L-DOPA treatment (by reducing PPP1R1B activation) [107], attenuates depression-like behavior by decreasing the expression of neuropeptides [124] and activates the endocannabinoid system [125–127]. In turn, the endocannabinoid system modulates synaptic (DA) transmission in the striatum of PD patients [128–130], restores homeostasis following DA depletion [131, 132] and exerts beneficial effects on cognition, mood, and nociception [126]. Therefore, physical exercise seems to exert a positive effect on the regulation of DA, neuropeptide, and endocannabinoid signaling. Moreover, these three signaling pathways are not only associated with L-DOPA-induced dyskinesia [133–138], a process that is mainly due to dysregulation in the DL, but are also involved in regulating VM-associated cognitive functions and behaviors [124, 139–144], supporting the notion that the anatomical and neurophysiological boundaries of the striatal domains regulating control of movement (DL) and (more) cognition-related processes (VM) may functionally overlap [145, 146].

In summary, the molecular pathways that are regulated in the SN and VM by both physical exercise and L-DOPA can be directly linked to clinical features of PD. Interestingly, the overall effects of physical exercise on these pathways seem to particularly improve the motor and behavioral clinical phenotype, whereas (chronic) L-DOPA-treatment can also cause adverse effects. Moreover, to our knowledge, physical exercise exerts—although it may counteract some L-DOPA-regulated pathways—no adverse effects on PD patients. To confirm the positive effects of physical exercise on cognitive function, future physical exercise studies in PD animal models and patients should include cognitive tests, e.g., the Y-maze, the water maze, or reversal learning tasks. Furthermore, these studies should aim at further elucidating the molecular pathways underlying physical exercise in relation to (chronic) L-DOPA treatment in animal models.

Taken together, our findings provide further evidence that physical exercise improves motor function in PD, while it also affects the regulation of non-motor brain areas of MPTP-treated mice. We found that physical exercise and L-DOPA exert opposite effects on molecular pathways in several PD-associated brain areas, including those involved in sleeping and cognitive function. Overall, the present study suggests that physical exercise has therapeutic potential, not only to improve motor function but it may also improve non-motor symptoms of PD—and perhaps even alleviate detrimental effects associated with (chronic) L-DOPA use.

## Electronic supplementary material

ESM 1 Punching locations per brain area. For each brain area—PFC (A), DL (B), VM (C), SN (D), VTA (E), and PPN (F)—the punching locations are visualized in a cross section adapted from the Paxinos mouse brain atlas [35]. Punching locations with a punching needle of 0.5 mm are shown with red circles, and blue circles indicate the location with 0.75-mm punch needles. (TIFF 2554 kb)

High resolution image (GIF 204 kb)

ESM 2 Optical density of fibers in the DL. The upper panel shows a representative picture for each of the four treatment groups and the lower panel shows the optic density in the DL per treatment group. *p < 0.05, means ± SEM, *n* = 5 for CNR, and MR and *n* = 4 for CR and MNR. *CC* corpus callosum, *DL* dorsolateral striatum. (TIFF 9852 kb)

High resolution image (GIF 670 kb)

ESM 3 Optical density of fibers in the VM. The upper panel shows a representative picture for each of the four treatment groups and the lower panel shows the optic density in the VM per treatment group. Means ± SEM, *n* = 5 for CNR and MR, and *n* = 4 for CR and MNR. *AC* anterior commissure, *VM* ventromedial striatum. (TIFF 9563 kb)

High resolution image (GIF 691 kb)

ESM 4 Validation of the RNAseq data using qPCR. The fold change of each mRNA in the RNAseq dataset and the brain area (PFC, DL, VM, SN, VTA) is shown above each graph, with the expression levels as measured by qPCR shown underneath. Expression levels are normalized to ACTB and YWHAZ and shown in arbitrary units (AU). Mean + SEM. The *p* values are indicated in each graph (Student’s *t* test). (PDF 1160 kb)

ESM 5 The effect of physical exercise on MPTP-mediated genes. Per brain area, the number of differentially expressed genes due to MPTP alone (in blue) or due to exercise in MPTP-treated mice (in gray), and their overlap are shown. The chance of observing this overlap is calculated with the hypergeometric distribution test and shown next to the overlapping area. Below the blue and gray circles, the total number of unique genes detected by RNAseq for each brain area is shown and also the number and percentage of overlapping genes that is regulated in opposite direction by MPTP and exercise in MPTP-treated mice. (TIFF 313 kb)

High resolution image (GIF 86 kb)

ESM 6 mRNAs differentially expressed in the DL due to physical exercise in MPTP-treated mice and regulated by RICTOR. The expression of the purple mRNAs is decreased by both physical exercise and RICTOR. The expression of orange mRNAs is increased by physical exercise and decreased by RICTOR. (TIFF 1309 kb)

High resolution image (GIF 146 kb)

ESM 7 mRNAs differentially expressed in the VTA due to physical exercise in MPTP-treated mice and regulated by RICTOR. The expression of the purple mRNAs is decreased by both physical exercise and RICTOR. (TIFF 1511 kb)

High resolution image (GIF 160 kb)

ESM 8 mRNAs differentially expressed in the PFC due to physical exercise in MPTP-treated mice and regulated by bicuculline/dalfampridine. mRNAs differentially expressed in the PFC due to physical exercise in MPTP-treated mice are shown in gray. Blue proteins are additional genes/proteins that are associated with PD through genetic and/or expression studies, whereas white proteins have no known link with PD. The direction of effect of physical exercise (measured) and bicuculline/dalfampridine (from literature) on the expression of these mRNAs is depicted through colored borders. Bicuculline-regulated proteins are shown with purple writing for the protein name, and familial PD proteins are shown with a blue border. (TIFF 1075 kb)

High resolution image (GIF 178 kb)

ESM 9 mRNAs differentially expressed in the PPN due to physical exercise in MPTP-treated mice and regulated by CREB1. mRNAs differentially expressed in the PPN due to physical exercise in MPTP-treated mice are shown in gray. Blue proteins are additional genes/proteins that are associated with PD through genetic and/or expression studies, whereas white proteins have no known link with PD. The direction of effect of physical exercise (measured) on the expression of these mRNAs is depicted through orange (increase) or purple (decrease) borders. Familial PD proteins are shown with a blue border. (TIFF 1180 kb)

High resolution image (GIF 166 kb)

ESM 10 Supplementary Tables 1–14, the detailed landscape descriptions, the references, and a list of abbreviations. (PDF 1852 kb)
