# Supplementary figures and images for: Physical Exercise Modulates L-DOPA-Regulated Molecular Pathways in the MPTP Mouse Model of Parkinson’s Disease
Source: Mol Neurobiol. 2017 Oct 10;55(7):5639–57. doi: 10.1007/s12035-017-0775-0 (PMC5994219; doi:10.1007/s12035-017-0775-0)

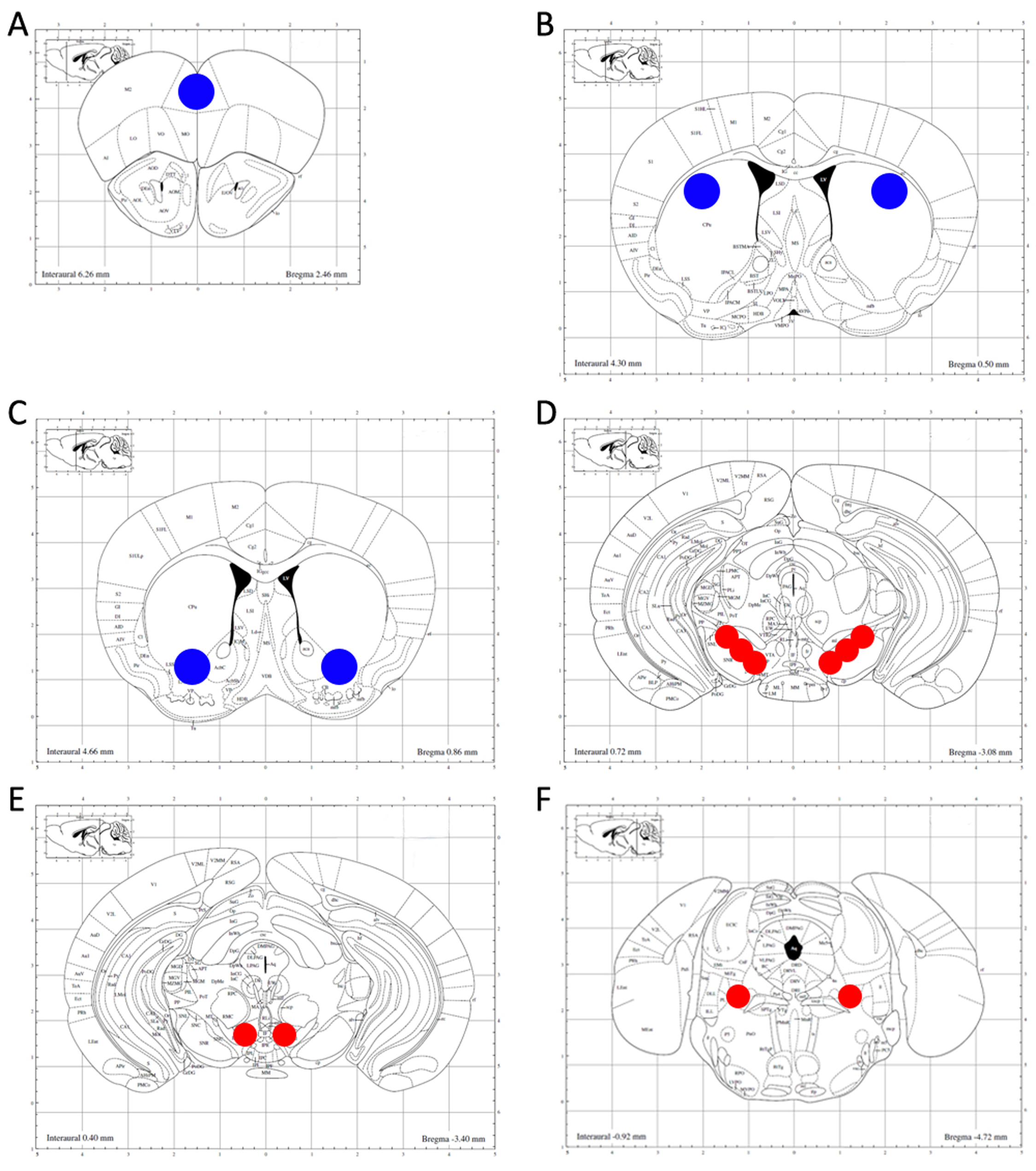

Supplement: Supplementary file 1 — Punching locations per brain area. For each brain area—PFC (A), DL (B), VM (C), SN (D), VTA (E), and PPN (F)—the punching locations are visualized in a cross section adapted from the Paxinos mouse brain atlas [35]. Punching locations with a punching needle of 0.5 mm are shown with red circles, and blue circles indicate the location with 0.75-mm punch needles. (TIFF 2554 kb) [file 12035_2017_775_MOESM1_ESM.tif]

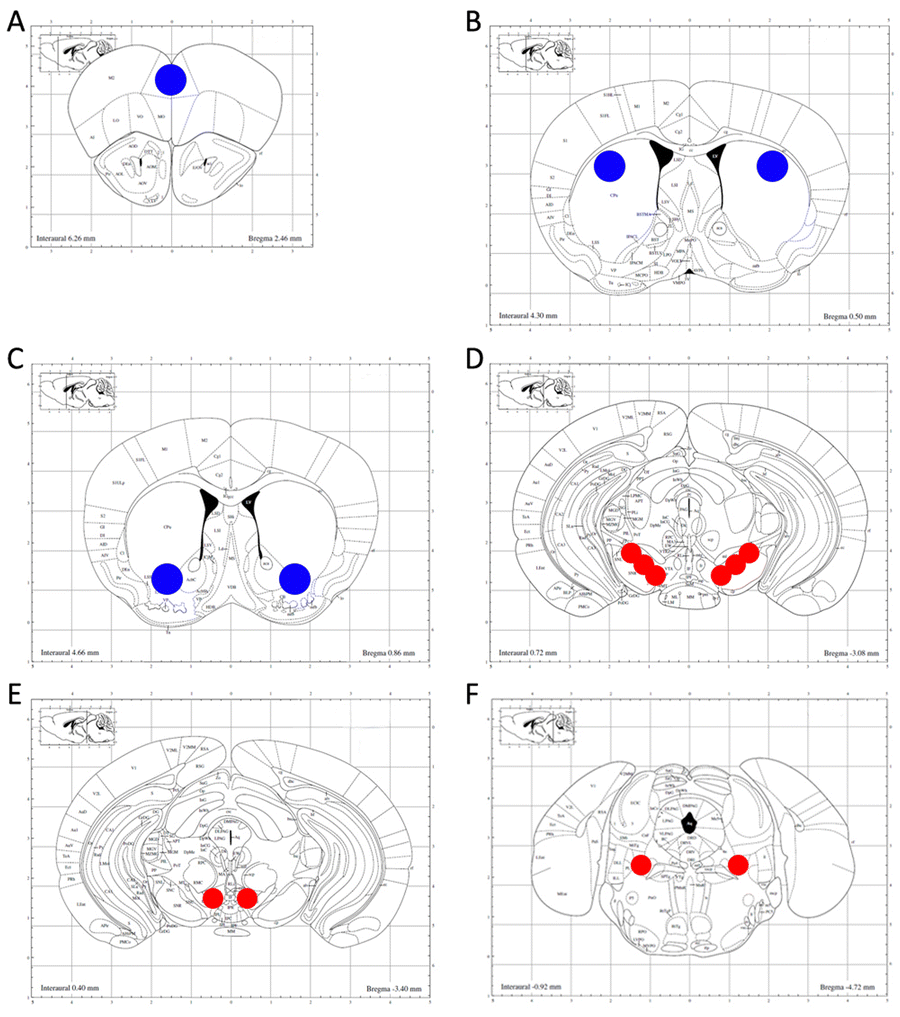

Supplement: Supplementary file 2 — High resolution image (GIF 204 kb) [file 12035_2017_775_Fig7_ESM.gif]

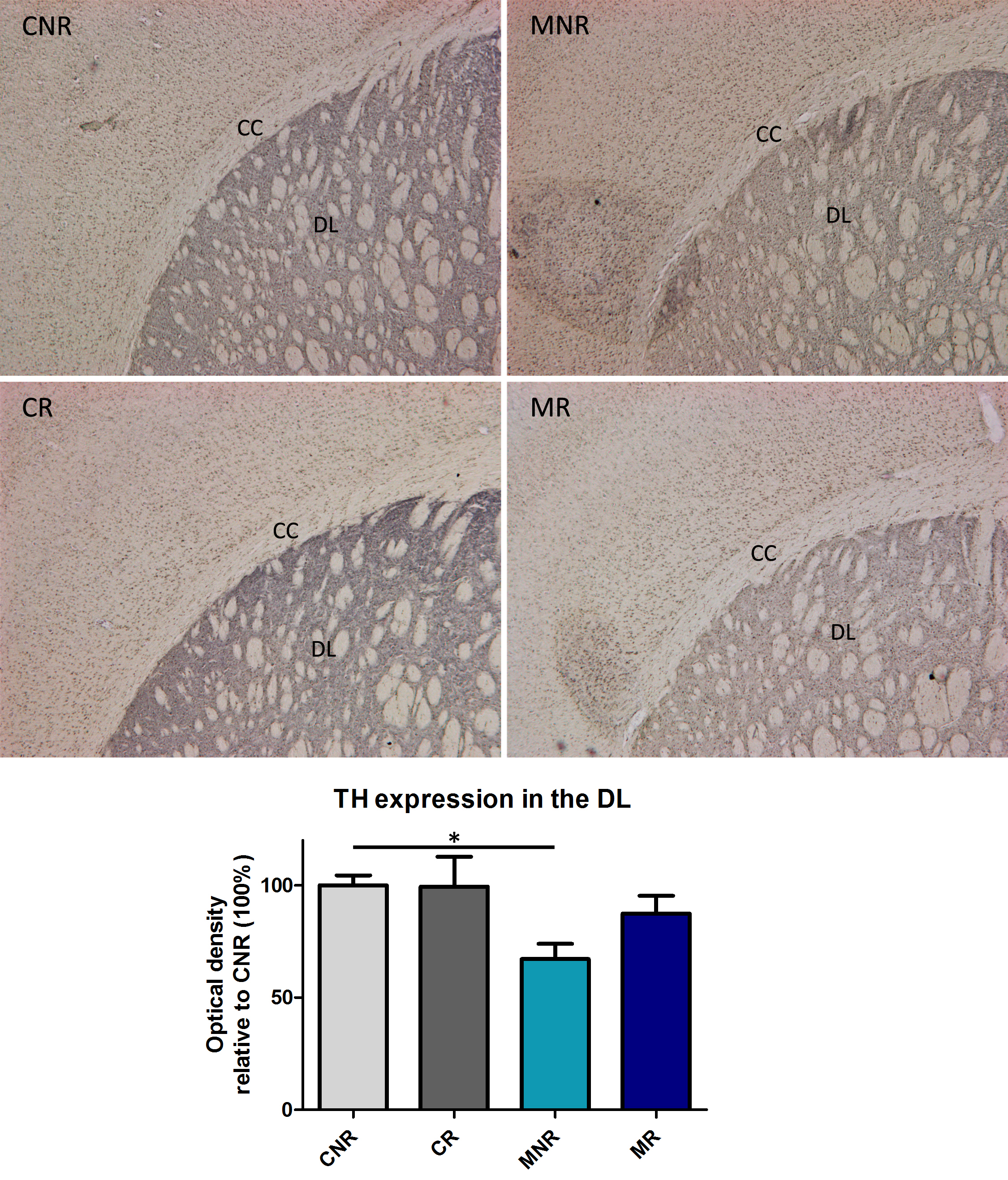

Supplement: Supplementary file 3 — Optical density of fibers in the DL. The upper panel shows a representative picture for each of the four treatment groups and the lower panel shows the optic density in the DL per treatment group. *p < 0.05, means ± SEM, n = 5 for CNR, and MR and n = 4 for CR and MNR. CC corpus callosum, DL dorsolateral striatum. (TIFF 9852 kb) [file 12035_2017_775_MOESM2_ESM.tif]

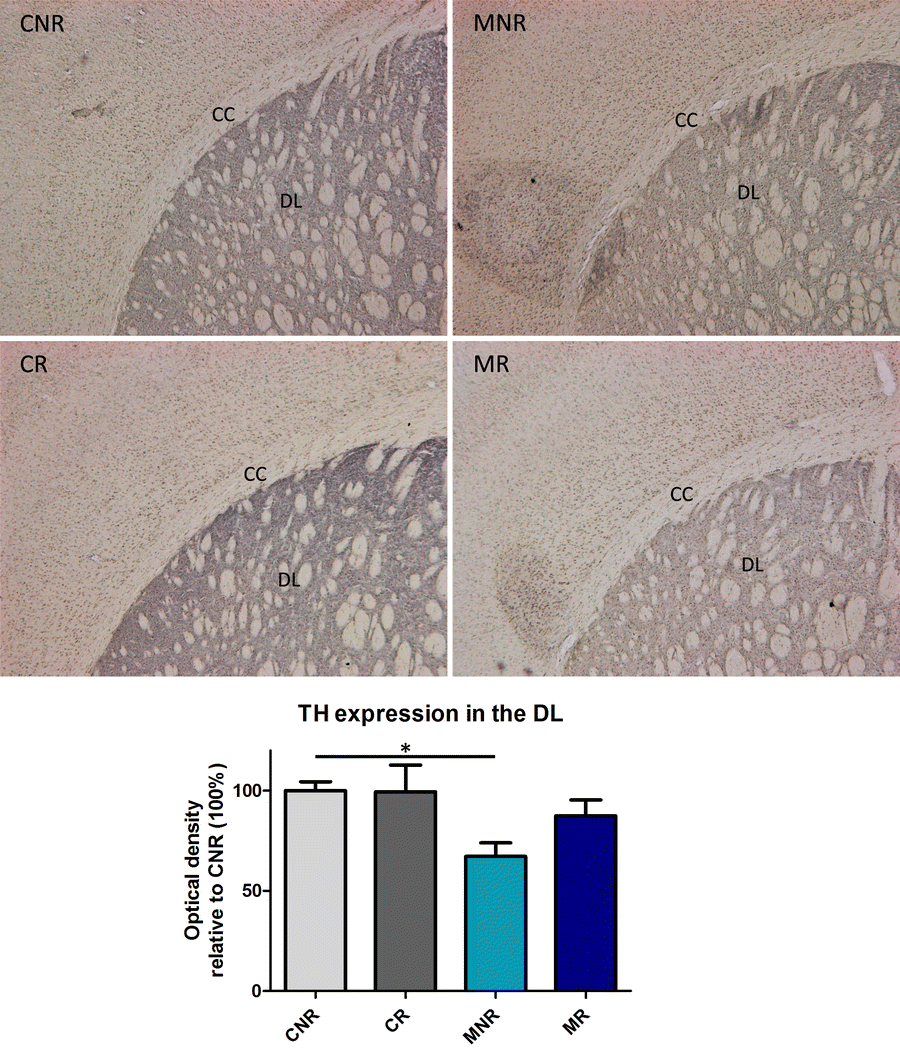

Supplement: Supplementary file 4 — High resolution image (GIF 670 kb) [file 12035_2017_775_Fig8_ESM.gif]

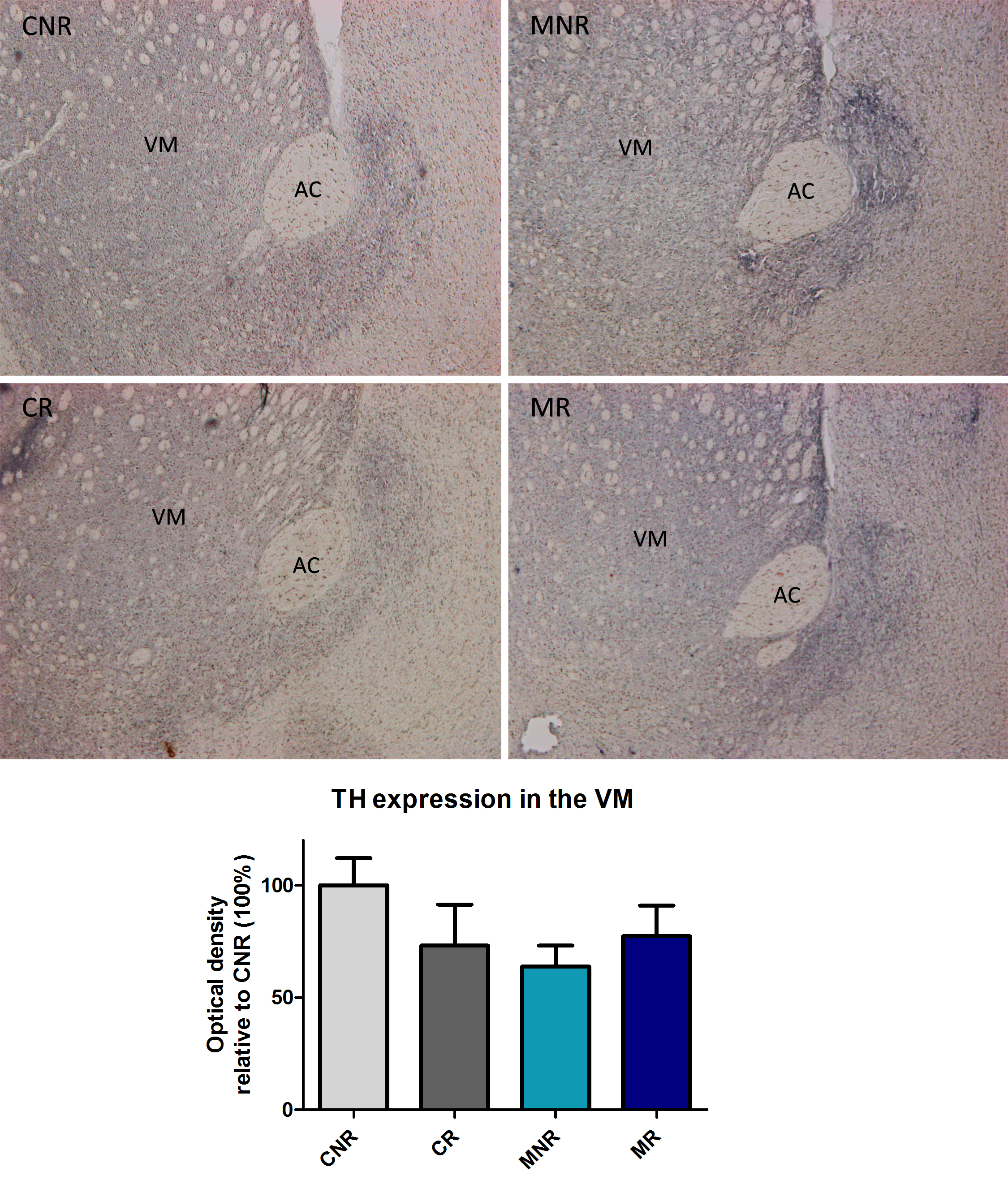

Supplement: Supplementary file 5 — Optical density of fibers in the VM. The upper panel shows a representative picture for each of the four treatment groups and the lower panel shows the optic density in the VM per treatment group. Means ± SEM, n = 5 for CNR and MR, and n = 4 for CR and MNR. AC anterior commissure, VM ventromedial striatum. (TIFF 9563 kb) [file 12035_2017_775_MOESM3_ESM.tif]

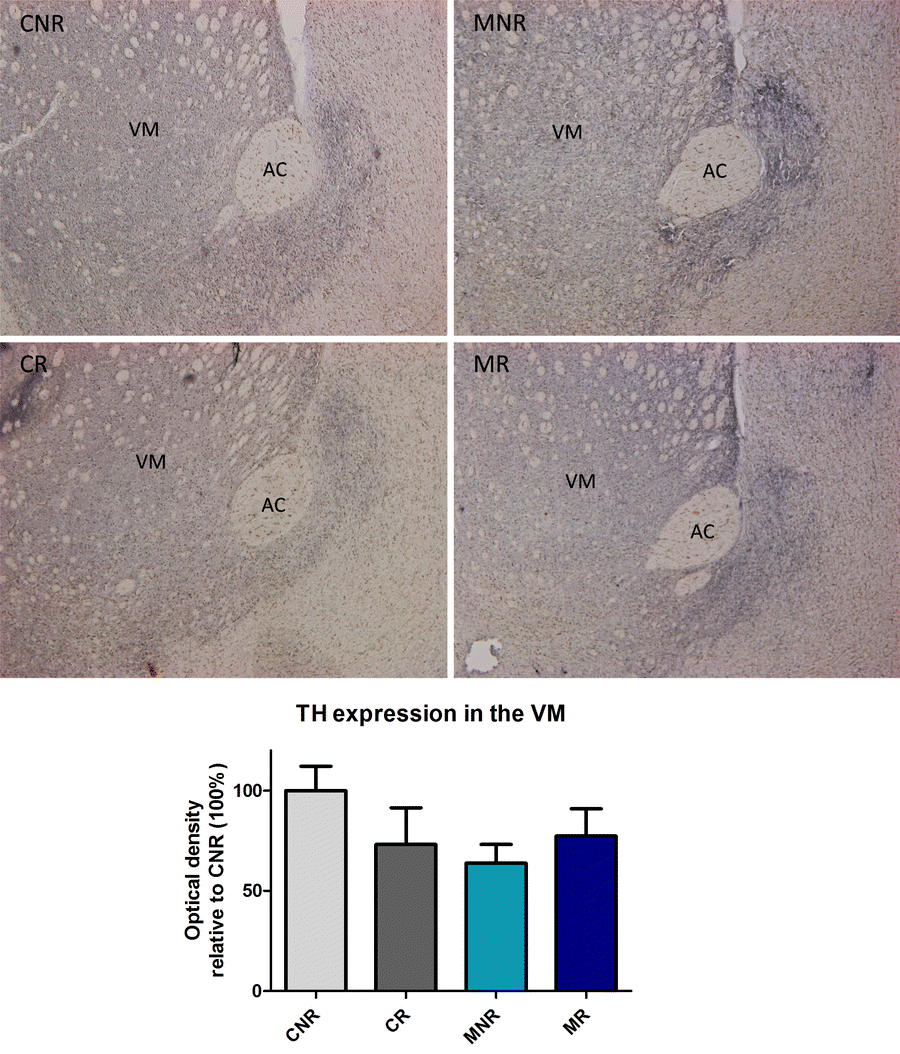

Supplement: Supplementary file 6 — High resolution image (GIF 691 kb) [file 12035_2017_775_Fig9_ESM.gif]

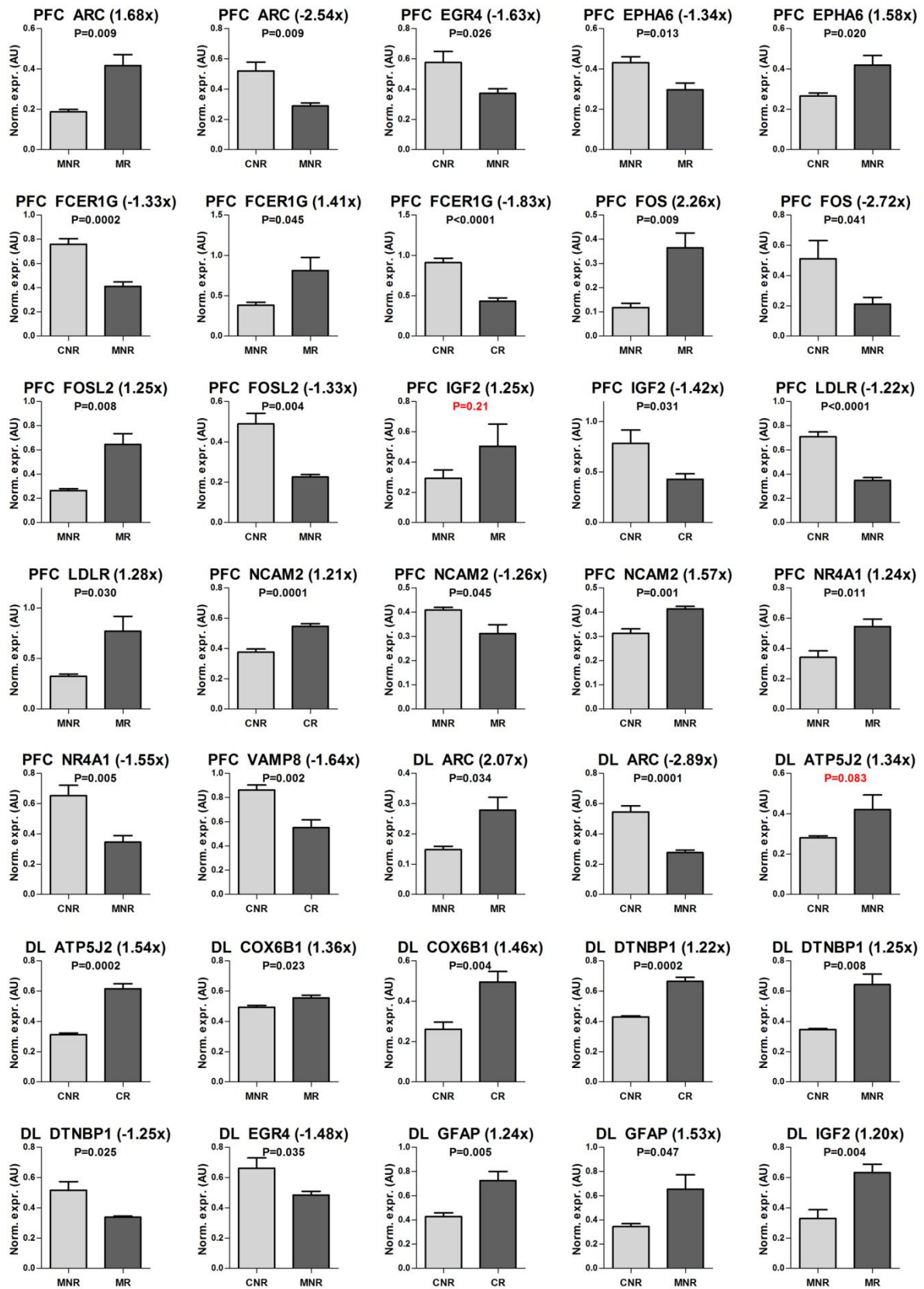

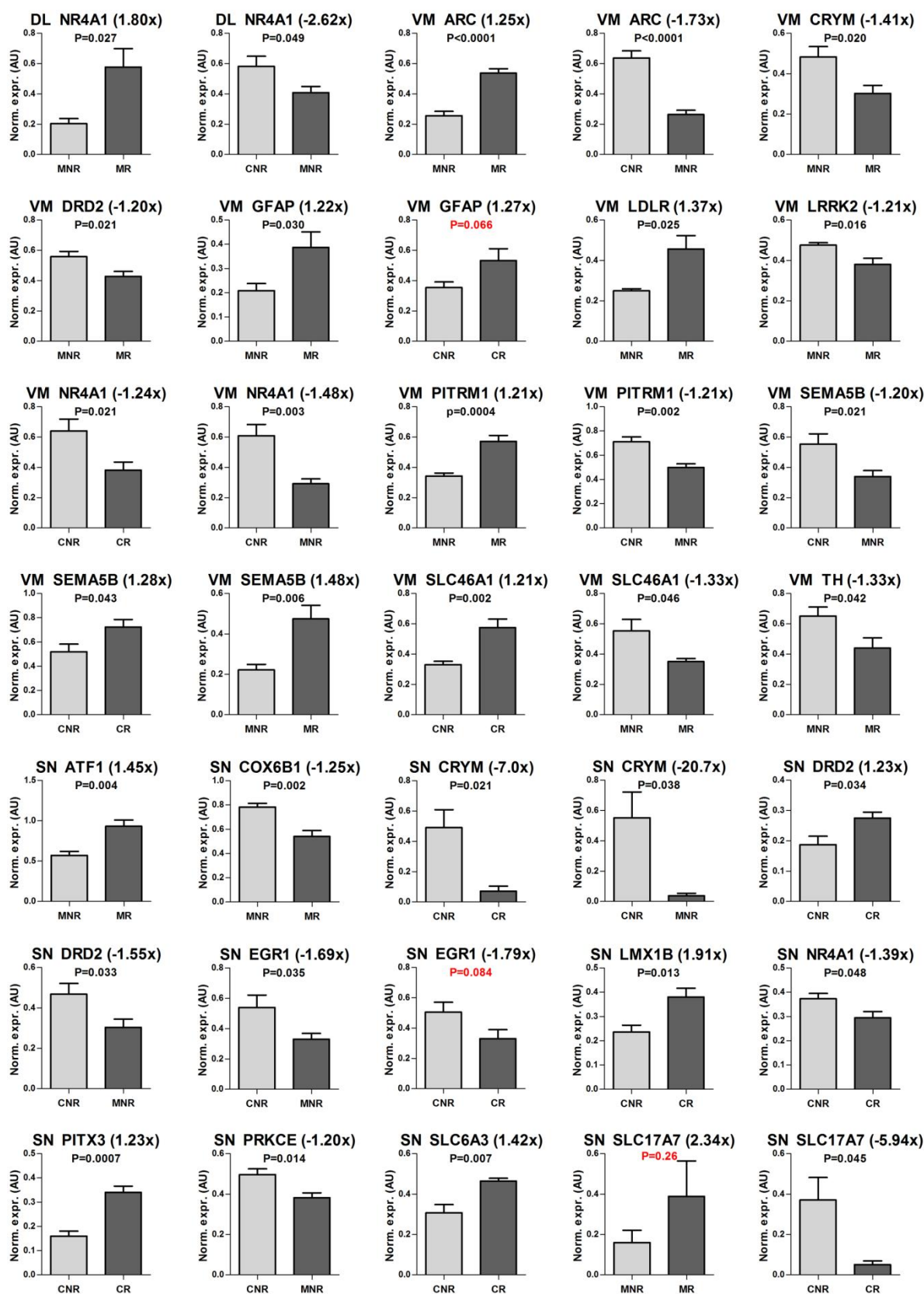

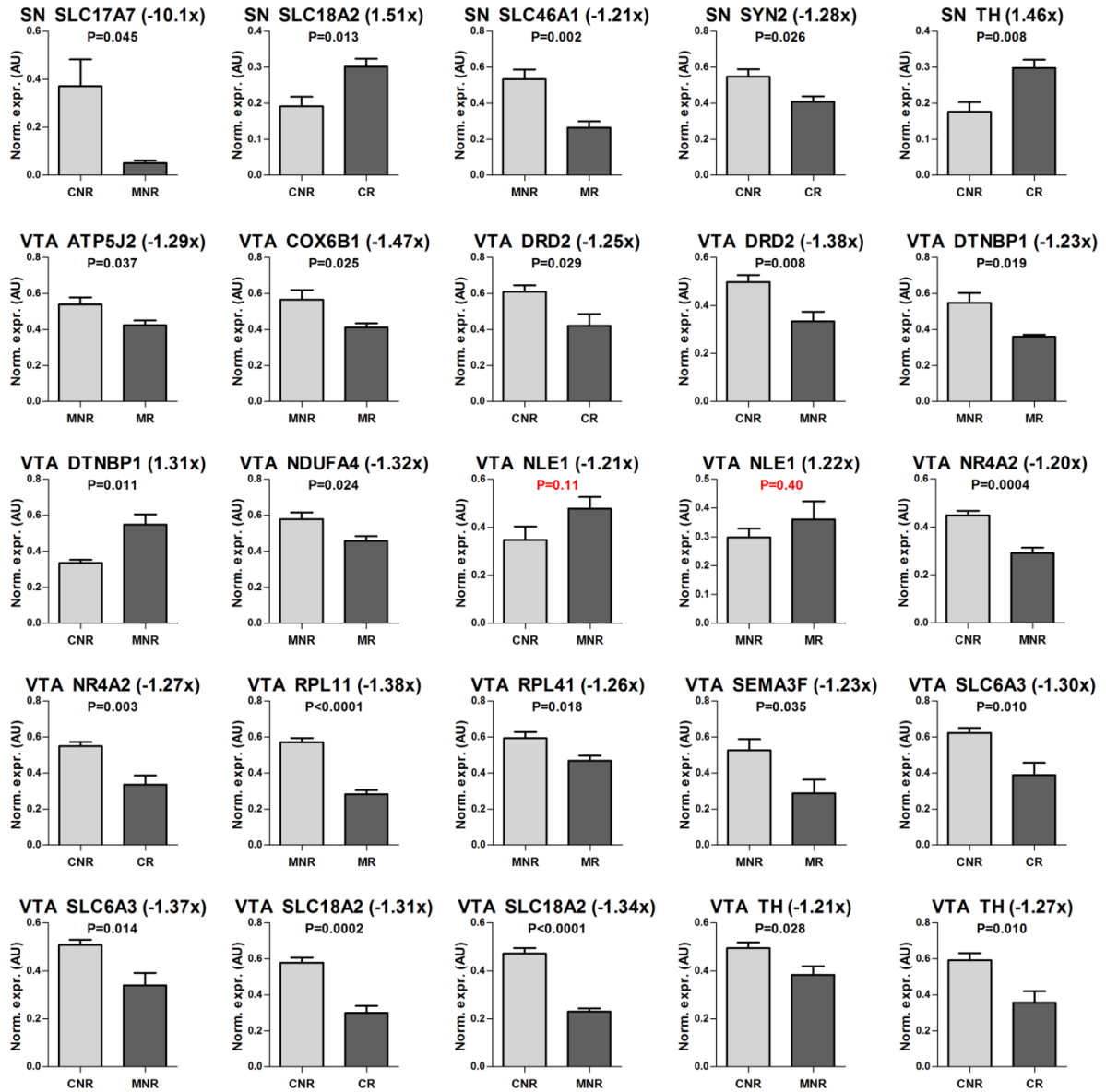

Supplement: Supplementary file 7 — Validation of the RNAseq data using qPCR. The fold change of each mRNA in the RNAseq dataset and the brain area (PFC, DL, VM, SN, VTA) is shown above each graph, with the expression levels as measured by qPCR shown underneath. Expression levels are normalized to ACTB and YWHAZ and shown in arbitrary units (AU). Mean + SEM. The p values are indicated in each graph (Student’s t test). (PDF 1160 kb) [file 12035_2017_775_MOESM4_ESM.pdf]

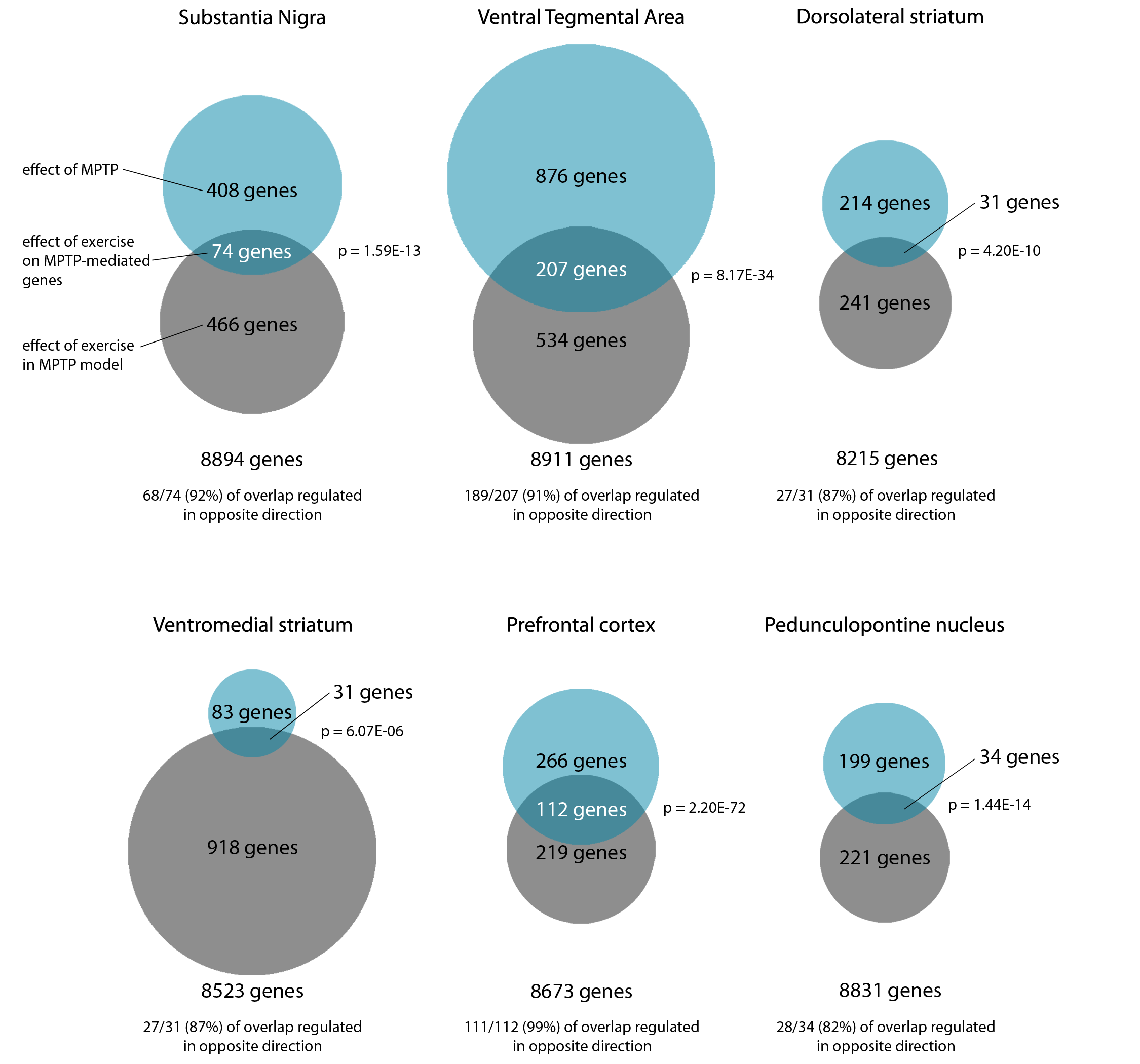

Supplement: Supplementary file 8 — The effect of physical exercise on MPTP-mediated genes. Per brain area, the number of differentially expressed genes due to MPTP alone (in blue) or due to exercise in MPTP-treated mice (in gray), and their overlap are shown. The chance of observing this overlap is calculated with the hypergeometric distribution test and shown next to the overlapping area. Below the blue and gray circles, the total number of unique genes detected by RNAseq for each brain area is shown and also the number and percentage of overlapping genes that is regulated in opposite direction by MPTP and exercise in MPTP-treated mice. (TIFF 313 kb) [file 12035_2017_775_MOESM5_ESM.tif]

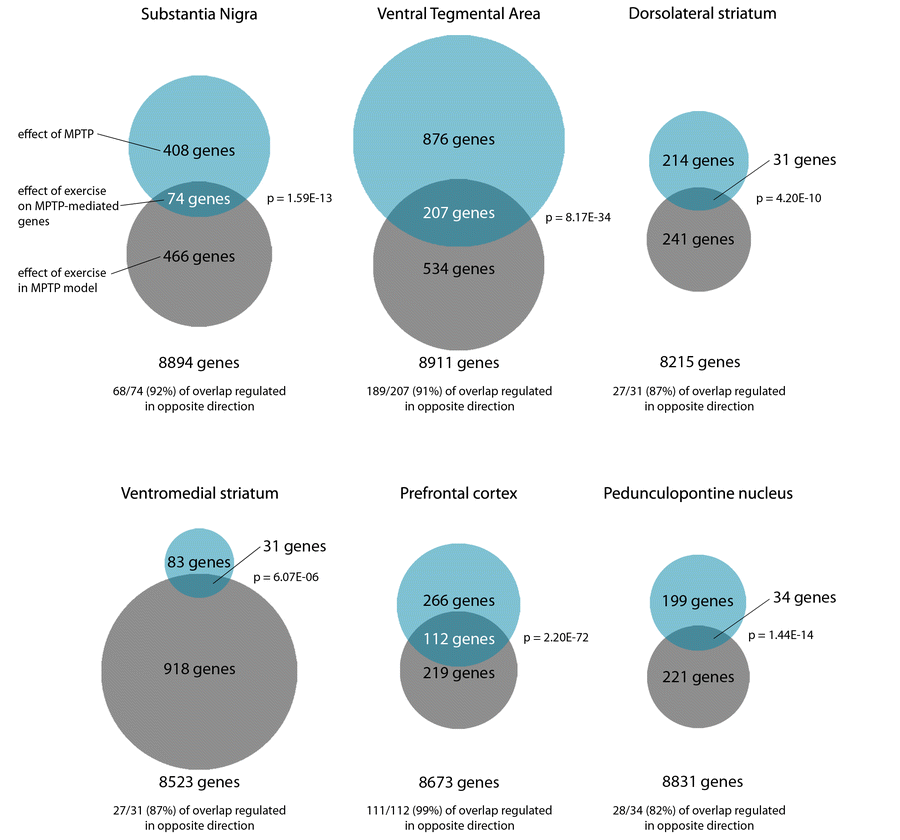

Supplement: Supplementary file 9 — High resolution image (GIF 86 kb) [file 12035_2017_775_Fig10_ESM.gif]

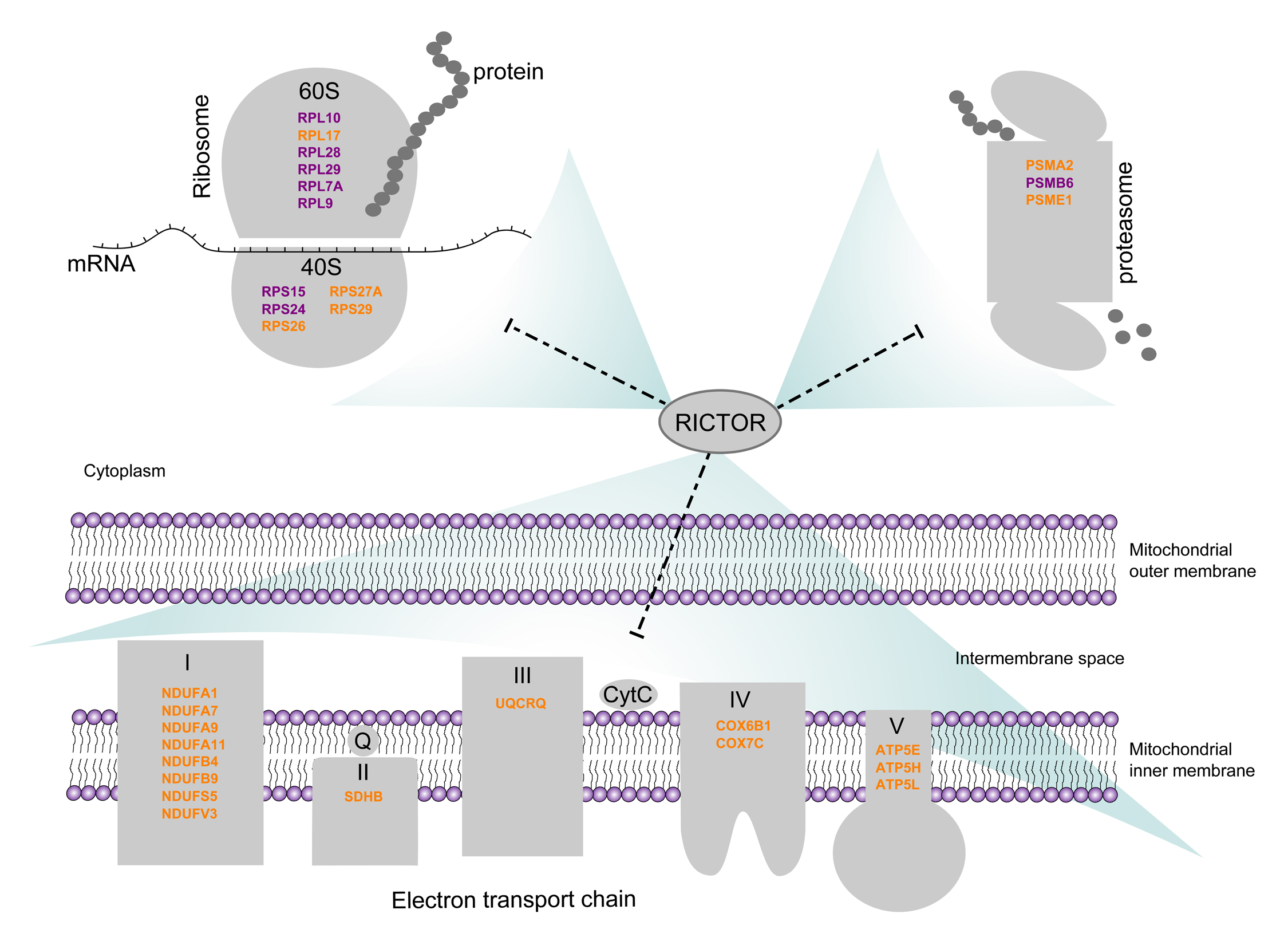

Supplement: Supplementary file 10 — mRNAs differentially expressed in the DL due to physical exercise in MPTP-treated mice and regulated by RICTOR. The expression of the purple mRNAs is decreased by both physical exercise and RICTOR. The expression of orange mRNAs is increased by physical exercise and decreased by RICTOR. (TIFF 1309 kb) [file 12035_2017_775_MOESM6_ESM.tif]

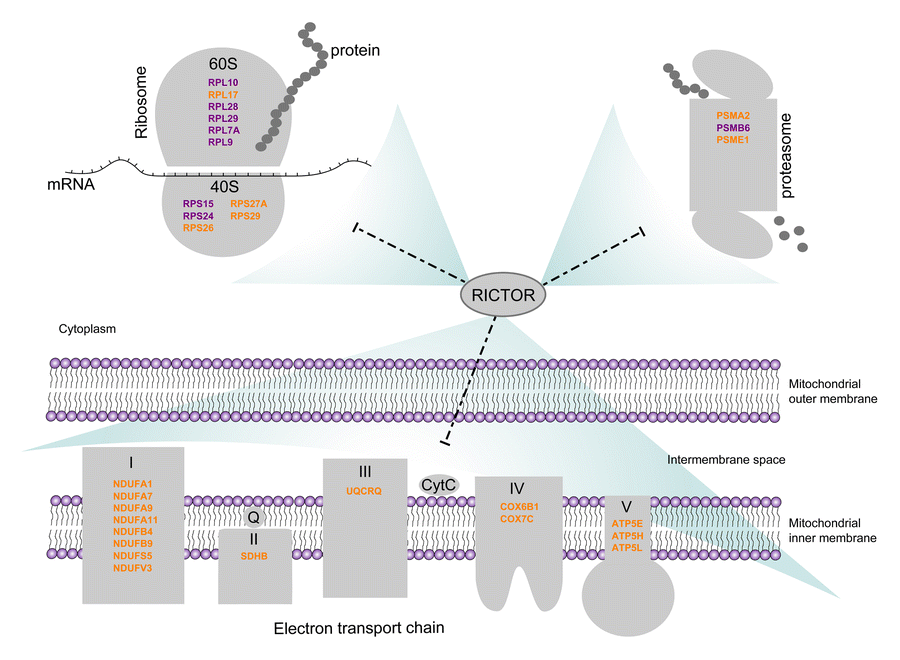

Supplement: Supplementary file 11 — High resolution image (GIF 146 kb) [file 12035_2017_775_Fig11_ESM.gif]

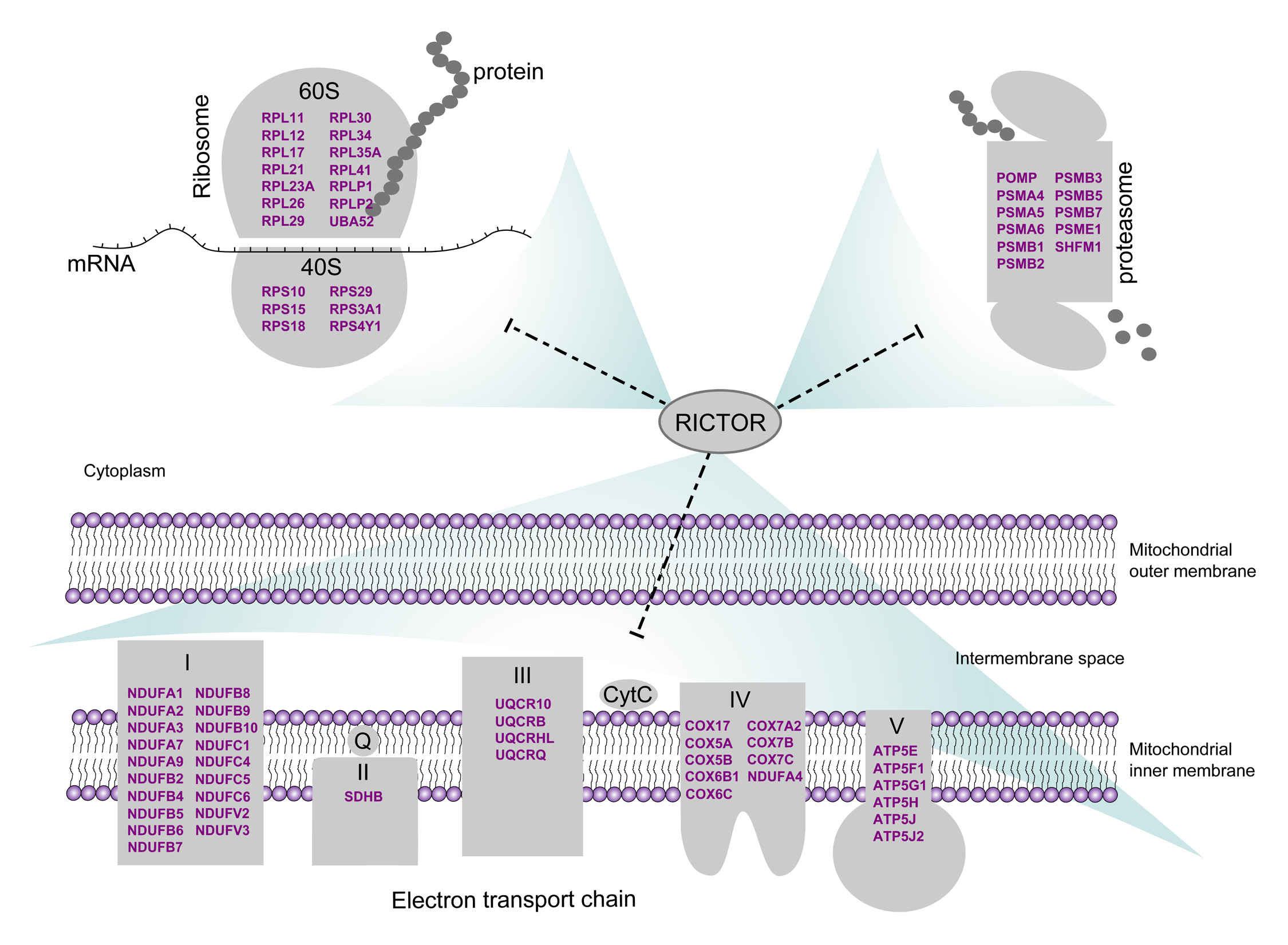

Supplement: Supplementary file 12 — mRNAs differentially expressed in the VTA due to physical exercise in MPTP-treated mice and regulated by RICTOR. The expression of the purple mRNAs is decreased by both physical exercise and RICTOR. (TIFF 1511 kb) [file 12035_2017_775_MOESM7_ESM.tif]

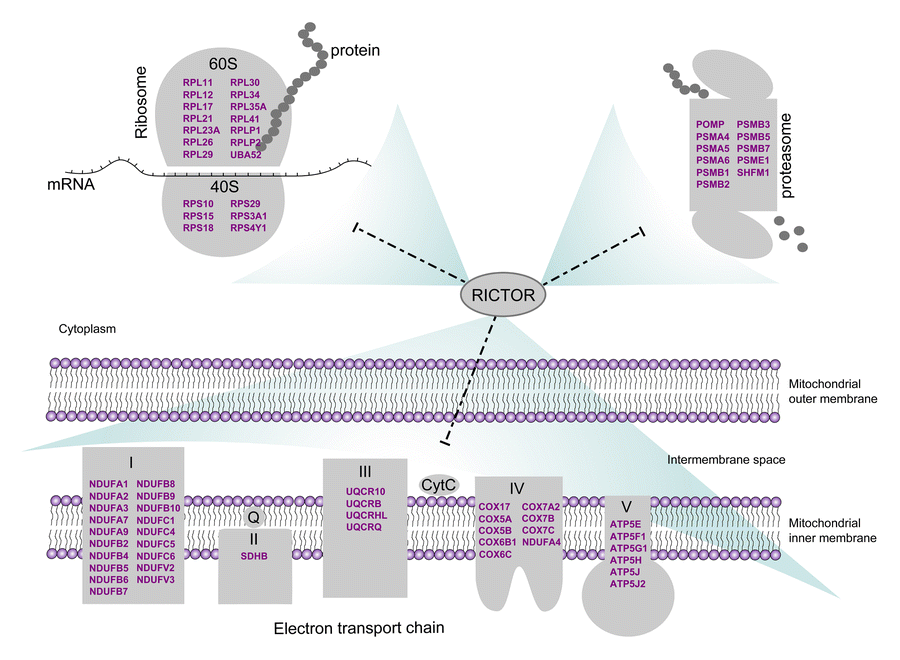

Supplement: Supplementary file 13 — High resolution image (GIF 160 kb) [file 12035_2017_775_Fig12_ESM.gif]

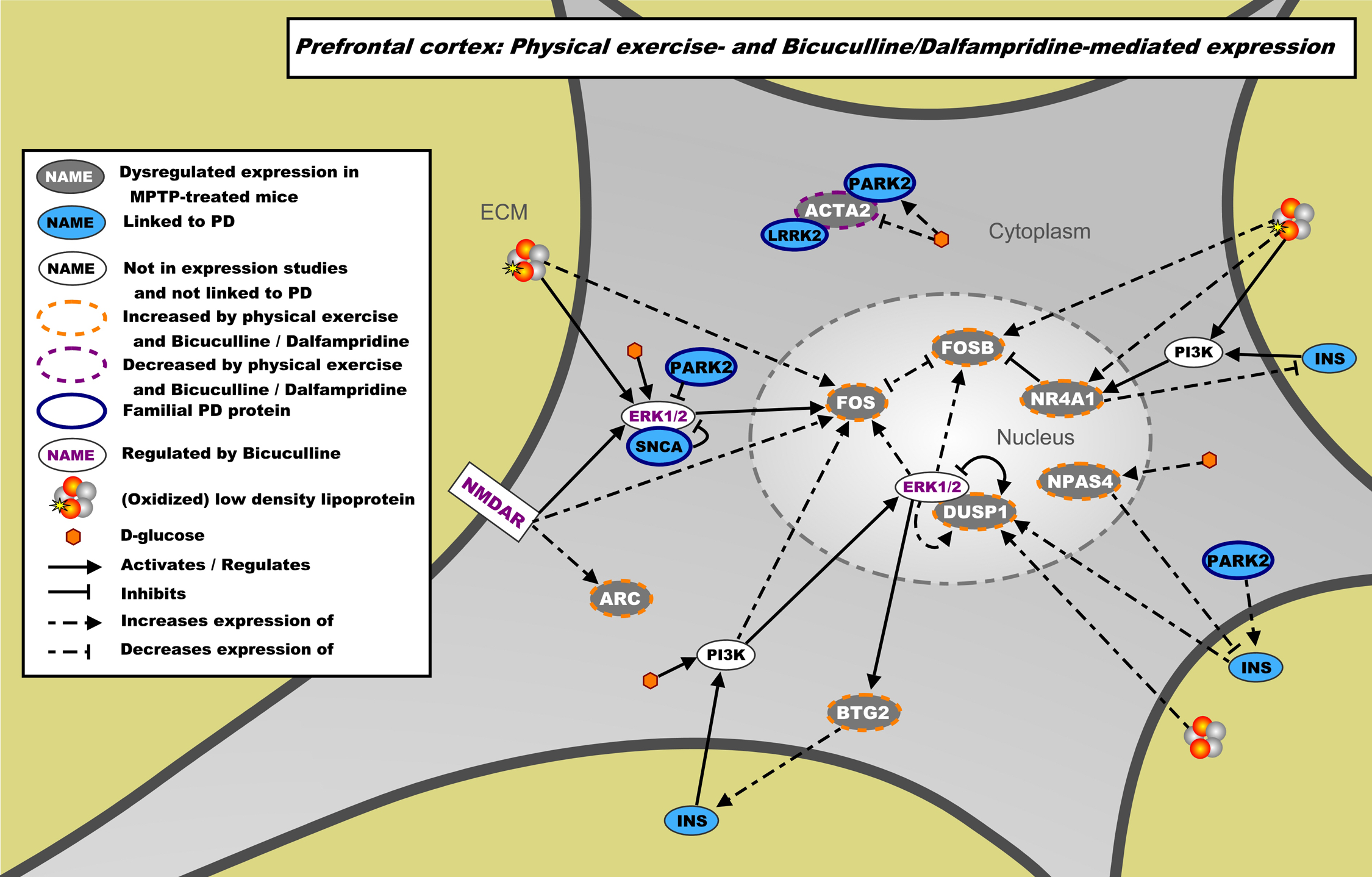

Supplement: Supplementary file 14 — mRNAs differentially expressed in the PFC due to physical exercise in MPTP-treated mice and regulated by bicuculline/dalfampridine. mRNAs differentially expressed in the PFC due to physical exercise in MPTP-treated mice are shown in gray. Blue proteins are additional genes/proteins that are associated with PD through genetic and/or expression studies, whereas white proteins have no known link with PD. The direction of effect of physical exercise (measured) and bicuculline/dalfampridine (from literature) on the expression of these mRNAs is depicted through colored borders. Bicuculline-regulated proteins are shown with purple writing for the protein name, and familial PD proteins are shown with a blue border. (TIFF 1075 kb) [file 12035_2017_775_MOESM8_ESM.tif]

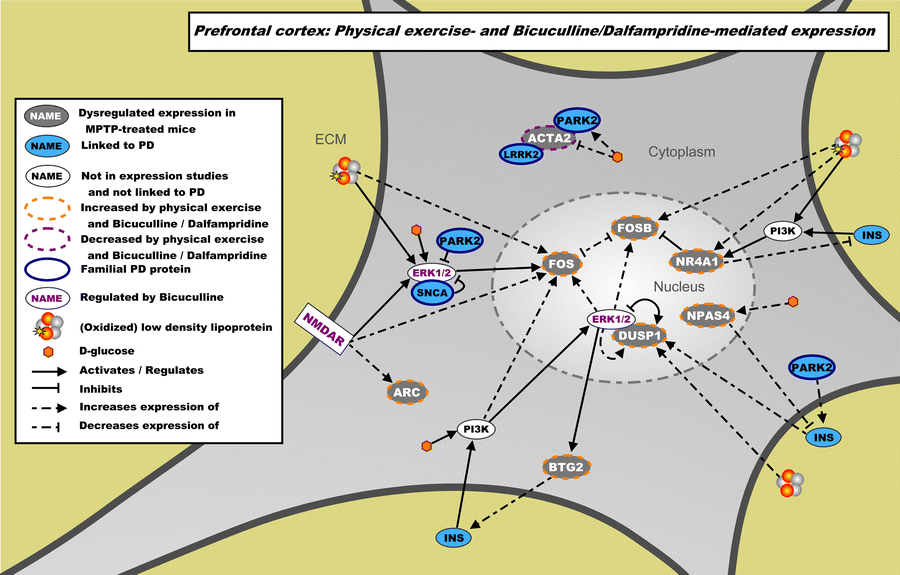

Supplement: Supplementary file 15 — High resolution image (GIF 178 kb) [file 12035_2017_775_Fig13_ESM.gif]

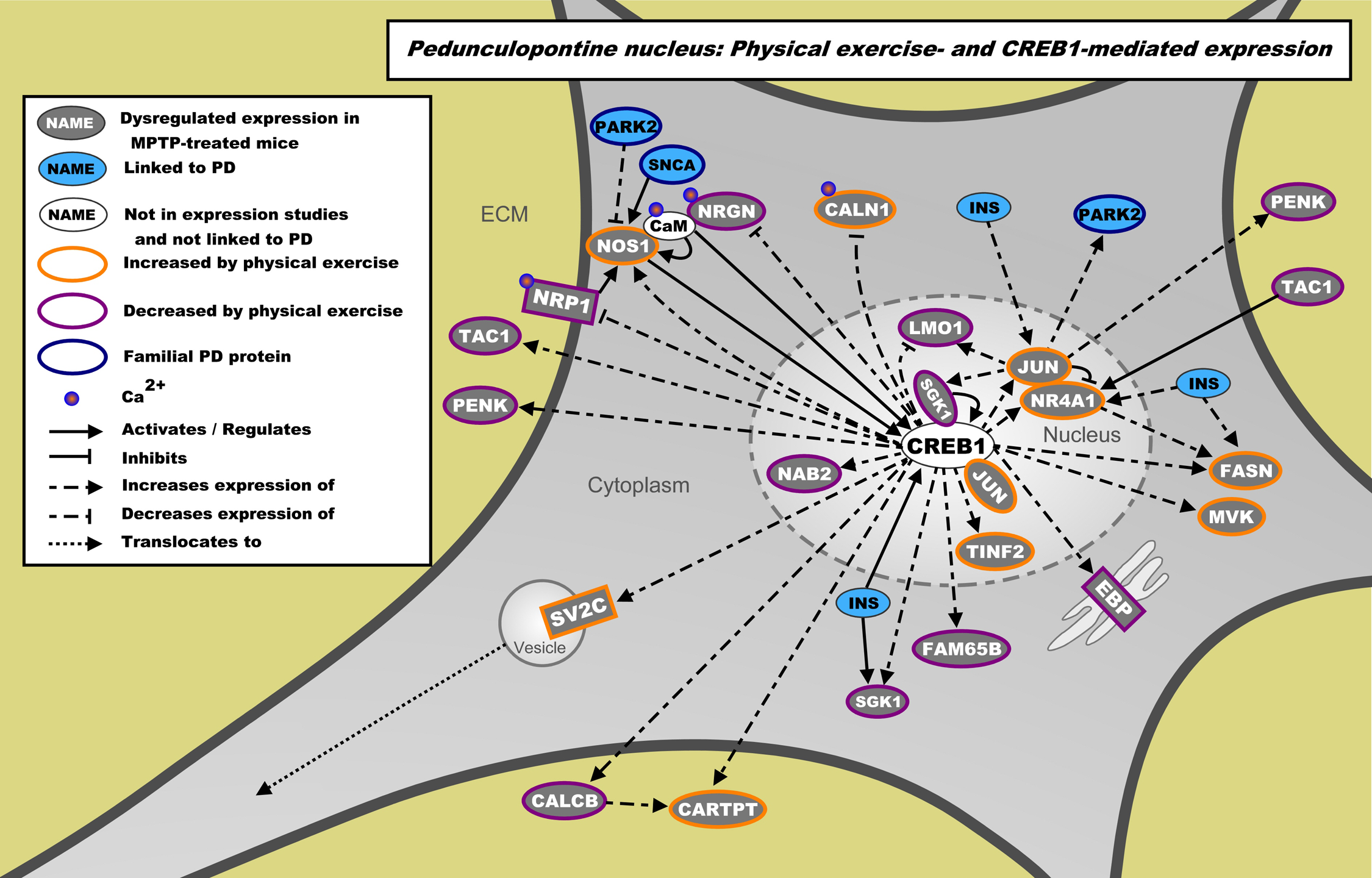

Supplement: Supplementary file 16 — mRNAs differentially expressed in the PPN due to physical exercise in MPTP-treated mice and regulated by CREB1. mRNAs differentially expressed in the PPN due to physical exercise in MPTP-treated mice are shown in gray. Blue proteins are additional genes/proteins that are associated with PD through genetic and/or expression studies, whereas white proteins have no known link with PD. The direction of effect of physical exercise (measured) on the expression of these mRNAs is depicted through orange (increase) or purple (decrease) borders. Familial PD proteins are shown with a blue border. (TIFF 1180 kb) [file 12035_2017_775_MOESM9_ESM.tif]

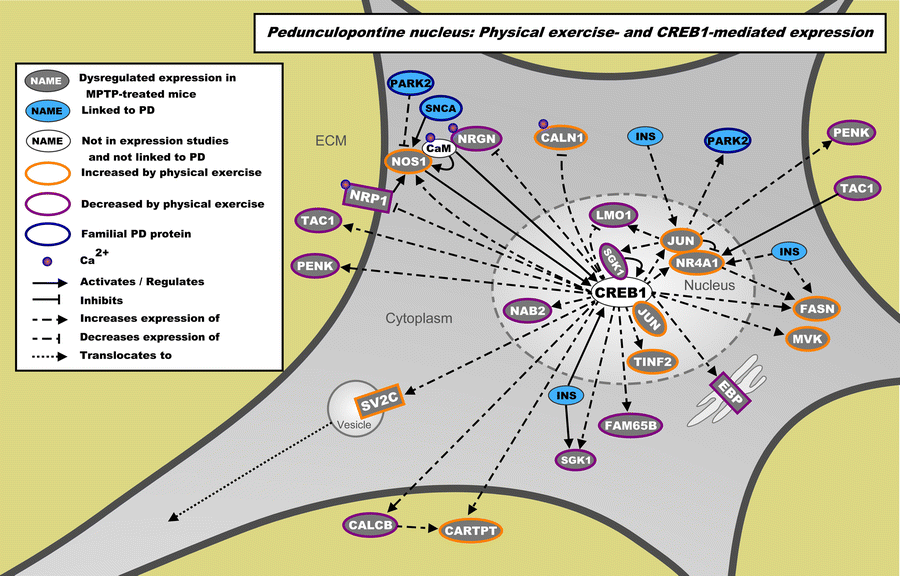

Supplement: Supplementary file 17 — High resolution image (GIF 166 kb) [file 12035_2017_775_Fig14_ESM.gif]
