# Supplementary material for: Physical Exercise Modulates L-DOPA-Regulated Molecular Pathways in the MPTP Mouse Model of Parkinson’s Disease
Source: Mol Neurobiol. 2017 Oct 10;55(7):5639–57. doi: 10.1007/s12035-017-0775-0 (PMC5994219; doi:10.1007/s12035-017-0775-0)
Supplement: Supplementary file 18 — Supplementary Tables 1–14, the detailed landscape descriptions, the references, and a list of abbreviations. (PDF 1852 kb) [file 12035_2017_775_MOESM10_ESM.pdf]

ONLINE RESOURCE

Physical exercise modulates L-DOPA-regulated molecular pathways in the MPTP mouse model of Parkinson's disease

Cornelius J.H.M. Klemann[1], Helena Xicoy[1,2],Geert Poelmans[1,3], Bas R. Bloem [4], Gerard J.M. Martens [1], Jasper E. Visser[1,4,5]

[1] Department of Molecular Animal Physiology, Donders Institute for Brain, Cognition and Behaviour, Radboud University, Nijmegen, The Netherlands

[2] Department of Cell Biology, Radboud University Medical Center, Nijmegen, The Netherlands

[3] Department of Human Genetics, Radboud University Medical Center, Nijmegen, The Netherlands

[4] Department of Neurology, Donders Institute for Brain, Cognition and Behaviour, Radboud University Medical Center, Nijmegen, The Netherlands

[5] Department of Neurology, Amphia Hospital, Breda, The Netherlands

Corresponding author: Jasper E. Visser, MD PhD
Department of Neurology
Radboud University Medical Center
P.O.Box 9101
6500 HB Nijmegen, The Netherlands
Telephone: +31 (0)24 361 66 00
Fax: +31 (0)24 354 11 22
Email: jasper.visser@radboudumc.nl

ONLINE RESOURCE CONTENTS

Supplementary Tables 1-14

Detailed description of the molecular landscapes

References

List of abbreviations

**Supplementary Table 1.** Primers used for validation of the RNAseq data by qPCR.		
Gene	FW/RV + gene location	Sequence (5′ to 3′)
ABCA1	FW3607-3626	TCCTTGGGGACAGAATTGCC
	RV3801-3779	TCTGAGAAACACTGTCCTCCTTT
ACTB	FW1055-1079	AAGATCAAGATCATTGCTCCTCCTG
	RV1228-1209	CGCAGCTCAGTAACAGTCCG
ARC	FW1086-1105	ACCACTCGACCAGTTCCTCT
	RV1267-1248	CCTGCACTTCCATACCCCTC
ATF1	FW724-745	TGGTTGTACAGACTGCATCAGG
	RV821-802	AGGAGAAGTCATCACCACGG
ATP5J2	FW224-244	CATCAACGTTCGGAAAGGCAG
	RV382-363	TTATGCTCGGCCATGCAATG
COX6B1	FW223-242	CCGCTGTGAGAAGGCAATGA
	RV325-307	GGCTGAGACCCATGACACG
CREB1	FW728-748	ACATTGCCATTACCCAGGGAG
	RV921-901	TGAGGCAGCTTGAACAACAAC
CRYM	FW793-812	GGTGCTGTATGTGGACTCCC
	RV953-934	CCACTGCCATCCCCAAAGAT
DRD2	FW891-909	ACAGGCGGAGAATGGATGC
	RV1045-1026	GCTATGTAGACCGTGGTGGG
DTNBP1	FW603-625	AAAGTAAGAGGAAGGAGCTTGAA
	RV723-704	TCGAAGAACTTCTGCCGCTC
EGR1	FW570-590	CCTGACCACAGAGTCCTTTTC
	RV684-665	GAAGCGGCCAGTATAGGTGA
EGR2	FW512-531	GTGGCGGGAGATGGCATGAT
	RV660-640	GGGTACTGTGGGTCAATGGAG
EGR4	FW271-289	TCCTGGAGGCGACTTCTTG
	RV457-437	AGACATGAGGTTGAAGAGGGC
EPHA6	FW727-747	AAATGGGTGGGATGCCATTAC
	RV855-836	GCAGCATCACGAGAGATCCA
EPOR	FW717-736	GGACACAAAGGGTGGAGGTC
	RV842-823	TCCAGAATCCGCTGAAGCTC
ETS1	FW901-920	ACTGTGTGCCCTGGGTAAAG
	RV1083-1064	TGCTCGATACCGTAGCTGAT
FCER1G	FW434-454	CTCTGTGCTTTGAAGGTTGGC
	RV601-582	GAGTCGAGGATCAGGGAAGG
FOS	FW624-644	CAGATACACTCCAAGCGGAGA
	RV779-760	CTGGGAAGCCAAGGTCATCG
FOSL2	FW456-475	CCTATCCACGCTCACATCCC
	RV593-571	GAGACAGCTGCTCATCTCTCCTT
GFAP	FW808-827	TGGCCACCAGTAACATGCAA
	RV995-976	CTCTAGGGACTCGTTCGTGC
IGF2	FW903-922	CAAACGTCATCGTCCCCTGA
	RV1074-1055	TGTGGGACGTGATGGAACTG
LDLR	FW2591-2610	CCTATTGCACTGGTTGCCCT
	RV2737-2718	ATCCTGGCTTCGGCAAATGT
LMX1A	FW1199-1218	CCCTATGGTGCTGAACCTCT
	RV1326-1306	TCAATGGGGTTTCCCACTCTG
LMX1B	FW810-829	GGGCCAAGAGGTTCTGTCAA
	RV972-953	GGAGTCGTTCCCTGGCATTT
LRRK2	FW591-611	CCAAAAACTGGGATGCAAAGC
	RV778-759	CATTGCTGCATGTAACCGCC
NCAM2	FW1849-1868	AGTACGCTCCCATGGAGTTC
	RV2043-2024	AAACTCTTGCCACTGCTTGG
NDUFA4	FW204-224	AGATGTCAGCTGGGACAGAAA
	RV388-369	GTGCGGATGGCTTCTGAAAG
NLE1	FW1074-1093	CTCTGGCTCAGACGACTTCA
	RV1265-1247	GAAGCTAGGTACTTGCCCG
NR4A1	FW1590-1609	AACATCCTGGCCTTCTCACG
	RV1691-1673	GAGCCCGTGTCGATCAGTG

NR4A2	FW1213-1232	TCCCTCCAATGAGGGTCTGT
	RV1319-1300	GCACCGTGCGCTTAAAGAAA
NR4A3	FW1322-1341	TTCTGACGGCCTCCATTGAC
	RV1471-1451	CAGCAGTGTTTGACCTGATGG
PITRM1	FW2536-2555	AATTGGTGACAGACCCCACC
	RV2652-2633	GGTCTGGATCAGCATACGGG
PITX3	FW452-470	CGTGCGGGTGTGGTTCAAG
	RV580-561	TACACCTCCTCGTAGGGTGG
PRKCE	FW2292-2311	TGTTCCAGATTCAGCGGTCC
	RV2407-2385	CAAATCCCTGTAGATCACTCCGT
PTGS2	FW1363-1382	TGGGGGAAGAAATGTGCCAA
	RV1523-1502	CAGCCATTTCCTTCTCTCCTGT
RPL11	FW351-371	ACACATTGACCTGGGCATCAA
	RV492-473	GCTGATTCTGTGTTTGGCCC
RPL41	FW245-263	CTGTGTGCTGCCATCGGTA
	RV350-330	GCAGAGGGACTGTTTTGGTTG
SEMA3F	FW1002-1021	CATCTGCCTCAACGATGACG
	RV1200-1179	AGAGCCTGAAGAGGTAAAGACA
SEMA5B	FW2240-2259	GGGAGGAGCGGTTCTGTAAT
	RV2411-2392	CAGGTCTTGAACTCCACGCC
SERPING1	FW1121-1140	TTGAAGGCCAAGGTGGGACA
	RV1299-1280	GGCATCGTCAGGTAAGTGGG
SLC17A7	FW1251-1270	CTTTTTGCGCAGTCGTCACA
	RV1433-1413	ACGTTAAACCCAGAGATGGCA
SLC18A2	FW1544-1474	GGGGTATGCTATCGGTCCCT
	RV1633-1614	TAATGGGGCAGTTGTGGTCC
SLC46A1	FW1151-1171	GTTCACAGGGTACGGATTGCT
	RV1333-1314	TTCAGAGTGGCCGGGTAGAT
SLC6A3	FW1835-1854	GGCTGGATCATTGCCACATC
	RV2003-1985	AATGGCGCAGCGTGAATTG
SYN2	FW945-964	CACCGAGAGATGCTTACGCT
	RV1084-1065	TAAGTTTGGGTGAGGGCCAC
TH	FW964-983	CACCTATGCACTCACCCGAG
	RV1121-1102	CCAGTACACCGTGGAGAGTT
VAMP8	FW342-362	TTGGAAGCCACGTCTGAACAC
	RV525-505	GAGGAGTAGGGTGGGATGGAA
YWHAZ	FW1176-1197	GCAAAAACAGCTTTCGATGAAG
	RV1345-1326	GCCGGTTAATTTTCCCCTCC

Supplementary Table 2. Enrichment analysis of MPTP-mediated mRNAs that are regulated in the opposite direction by exercise in the MPTP model (see also Supplementary Figure 5**)**. Shown are the results of the analyses in the SN (68 genes), VTA (189 genes), DL (27 genes), VM (27 genes), PFC (111 genes) and PPN (28 genes). The top 10 upstream regulators, top 5 canonical pathways and top 5 annotations of the categories 'Diseases and disorders' and 'Molecular and Cellular Functions' are displayed, as well as their respective z-score, p-value and number of genes involved (#). The z-scores are based on the effect of exercise in the MPTP-treated mice (MPTP + physical exercise vs. MPTP), which means that they are in the opposite direction due to MPTP alone (MPTP vs. Control). All p-values are calculated using the Fisher's Exact Test, and the p-values for the canonical pathways and the categories 'Diseases and disorders' and 'Molecular and Cellular Functions' are corrected for multiple testing using the Benjamini-Hochberg correction. For the category 'Molecular and Cellular Functions', only the annotations with a significant z-score (i.e. <-2 (in red) or >2 (in green)) are shown. Annotations with only 1 (target) gene were discarded.

(1) Upstream Regulators

SN				VTA				DL			
**Upstream Regulator**	**z-score**	**p-value**	**#**	**Upstream Regulator**	**z-score**	**p-value**	**#**	**Upstream Regulator**	**z-score**	**p-value**	**#**
NR3C1	0.055	4.22E-04	8	Inosine	-2.433	1.96E-06	6	Dalfampridine	2.000	3.48E-08	4
HOXA11	-	5.61E-04	2	KDM5A	2.828	5.42E-06	8	Bicuculline	1.941	7.92E-08	4
HMGA1	-	6.70E-04	4	RICTOR	3.162	2.60E-05	10	MECP2	-	6.75E-06	4
NRG2	-	7.52E-04	3	Dexamethasone phosphate	-1.000	2.65E-05	4	2-amino-5-phosphonovaleric acid	-1.960	1.45E-05	4
PTPN11	-	8.21E-04	3	Mt1		1.65E-04	3	Tacedinaline	-	1.61E-05	2
Plerixafor	-	1.35E-03	2	MYC	-2.540	1.81E-04	19	MAPK1	0.200	2.63E-05	5
GLI1	-1.000	2.15E-03	4	E. coli B4 lipopolysaccharide	-2.343	2.17E-04	9	Caffeine	-	4.41E-05	3
TFAP2A	-	2.25E-03	3	STAT4	-2.630	2.22E-04	8	pCPT-cAMP	-	4.83E-05	2
Beta-estradiol	0.841	2.65E-03	12	Interferon beta-1a	-	2.86E-04	6	Atorvastatin	0.849	5.66E-05	4
Propylthiouracil	-	2.98E-03	3	ZFHX3	-	4.32E-04	5	HNRNPAB	-	7.07E-05	2

(2) Canonical Pathways

SN				VTA				DL			
**Canonical pathway**	**z-score**	**p-value**	**#**	**Canonical pathway**	**z-score**	**p-value**	**#**	**Canonical pathway**	**z-score**	**p-value**	**#**
-				-				Nur77 Signaling in T lymphocytes	-	4.80E-02	2
								Calcium-induced T lymphocyte apoptosis	-	4.80E-02	2

(3) Diseases and Disorders

SN				VTA				DL			
**Annotation**	**z-score**	**p-value**	**#**	**Annotation**	**z-score**	**p-value**	**#**	**Annotation**	**z-score**	**p-value**	**#**
Tumorigenesis of tissue	-	3.58E-02	60	Severity of renal lesion	-	4.33E-02	2	Unstable hemoglobin disease	-	5.09E-04	2
Cancer	0.371	3.58E-02	62	Complement component C1q deficiency	-	4.33E-02	2	Epileptic seizure	-	5.93E-04	5
Epithelial cancer	-	3.58E-02	59	Degeneration of renal tubule	-	4.33E-02	2	Inflammation of organ	-0.847	1.72E-02	8
Abdominal cancer	-	3.58E-02	58	Formation of renal-cell carcinoma	-	4.33E-02	2	Vasculitis	-	3.14E-02	2
Malignant solid tumor	-1.000	3.58E-02	61	Nephromegaly	-	4.33E-02	2	Movement disorders	-	4.27E-02	6

(4) Molecular and Cellular Functions

SN				VTA				DL			
**Annotation**	**z-score**	**p-value**	**#**	**Annotation**	**z-score**	**p-value**	**#**	**Annotation**	**z-score**	**p-value**	**#**
-				Adhesion of endothelial cells	-2.049	4.33E-02	8	Differentiation of cells	2.299	3.20E-02	8
				Quantity of heavy metal	2.191	1.32E-03	5				
				Inflammation of body region	2.091	1.20E-02	19				
				Expression of RNA	-2.277	4.10E-02	32				

Supplementary Table 2. Enrichment analysis of MPTP-mediated mRNAs that are regulated in the opposite direction by exercise in the MPTP model. (continued)

(1) Upstream Regulators

VM				PFC				PPN			
**Upstream Regulator**	**z-score**	**p-value**	**#**	**Upstream Regulator**	**z-score**	**p-value**	**#**	**Upstream Regulator**	**z-score**	**p-value**	**#**
Trichostatin A	-0.458	2.46E-05	6	Dalfampridine	2.828	1.21E-12	8	Alpha-amanitin	-	9.74E-05	2
Actin	-	8.35E-05	2	Bicuculline	2.774	6.87E-12	8	CBX2	-	9.74E-05	2
Brd4	-	9.74E-05	2	GNB2	-	6.40E-07	5	POMC	-	1.33E-04	3
L-dopa	1.432	6.17E-04	5	GNB1	-	8.40E-07	5	Propylthiouracil	-	1.84E-04	3
PSEN1	-	6.51E-04	4	CREB1	2.591	9.44E-07	13	ASAH1	-	3.72E-04	2
MBTD1	-	7.40E-04	2	RNF20	-	1.11E-06	4	KMT2D	-	8.07E-04	3
APLN	-	9.93E-04	2	tacedinaline	-	1.73E-06	3	CRH	-	1.90E-03	2
GLI1	-	1.19E-03	3	morphine	0.629	2.58E-06	7	25-hydroxycholesterol	-	1.96E-03	2
ARNT2	-	1.31E-03	3	atorvastatin	2.019	3.52E-06	8	GnRH-A	-	2.63E-03	2
BRD2	-	1.33E-03	2	2-amino-5-phosphonovaleric acid	-1.942	3.61E-06	7	5-N-ethylcarboxamido adenosine	-	5.51E-03	2

(2) Canonical Pathways

VM				PFC				PPN			
**Canonical pathway**	**z-score**	**p-value**	**#**	**Canonical pathway**	**z-score**	**p-value**	**#**	**Canonical pathway**	**z-score**	**p-value**	**#**
-				-				-			

(3) Diseases and Disorders

VM				PFC				PPN			
**Annotation**	**z-score**	**p-value**	**#**	**Annotation**	**z-score**	**p-value**	**#**	**Annotation**	**z-score**	**p-value**	**#**
Toxic epidermal necrolysis	-	1.62E-02	2	Gastrointestinal adenocarcinoma	-	4.57E-06	69	Aggregation of T lymphocytes	-	1.67E-02	2
Lichen planus	-	1.62E-02	3	Abdominal carcinoma	-	4.57E-06	81	Epithelial cancer	-	1.67E-02	23
Stevens-Johnson syndrome	-	1.62E-02	2	Epilepsy	-	4.57E-06	14	Erythrocytosis	-	1.96E-02	2
Transverse myelitis	-	1.62E-02	2	Seizures	0.664	1.06E-05	15	Abdominal cancer	-	2.07E-02	22
Malaria	-	2.17E-02	2	Seizure disorder	0.744	2.43E-05	16	Digestive system cancer	-	2.56E-02	21

(4) Molecular and Cellular Functions

VM				PFC				PPN			
**Annotation**	**z-score**	**p-value**	**#**	**Annotation**	**z-score**	**p-value**	**#**	**Annotation**	**z-score**	**p-value**	**#**
-				-				-			

Supplementary Table 3. Enrichment analysis of mRNAs differentially expressed in the SN. Ingenuity annotations of genes differentially expressed by running alone (CNR vs. CR; 514 genes), MPTP alone (CNR vs. MNR; 482 genes) and by running in MPTP-treated mice (MNR vs. MR; 540 genes). The top 10 upstream regulators, top 5 canonical pathways and top 5 annotations of the categories 'Diseases and disorders' and 'Molecular and Cellular Functions' are displayed, as well as their respective z-score, p-value and number of genes involved (#). All p-values are calculated using the Fisher's Exact Test, and the p-values for the canonical pathways and the categories 'Diseases and disorders' and 'Molecular and Cellular Functions' are corrected for multiple testing using the Benjamini-Hochberg correction. For the category 'Molecular and Cellular Functions', only the annotations with a significant z-score (i.e. <-2 (in red) or >2 (in green)) are shown. Annotations with only 1 (target) gene were discarded.

**(1) Upstream Regulators**											
**Physical exercise vs. Control**				**MPTP vs. Control**				**MPTP + Physical exercise vs. MPTP**			
**Upstream Regulator**	**z-score**	**p-value**	**#**	**Upstream Regulator**	**z-score**	**p-value**	**#**	**Upstream Regulator**	**z-score**	**p-value**	**#**
CREB1	1.922	3.44E-19	49	CREB1	2.325	1.58E-14	42	L-dopa	-0.669	2.20E-07	36
HTT	-0.138	6.80E-17	53	HTT	1.618	2.75E-12	45	HNF4A	-1.320	4.09E-06	56
Beta-estradiol	-0.310	9.94E-15	85	L-dopa	-0.903	2.91E-12	44	TP53	-2.340	5.70E-06	74
L-dopa	-1.778	2.23E-14	48	BDNF	-2.313	1.06E-09	25	R5020	-2.397	8.98E-06	9
BDNF	-2.747	1.97E-12	29	ATN1	-	1.16E-09	17	ARHGDIG	0.447	3.09E-05	5
Dopamine	-0.033	2.20E-11	17	HDAC4	-	4.12E-08	14	Benzene	0.453	3.28E-05	8
Forskolin	-1.524	1.09E-10	41	FGF2	-0.194	8.65E-08	24	PEBP4	-	3.91E-05	3
Nicotine	1.300	1.11E-10	21	NR3C1	-1.329	9.38E-08	35	EIF4E	-2.863	4.29E-05	12
FGF2	-1.038	3.49E-10	28	Okadaic acid	0.067	1.28E-07	12	ACVRL1	-	5.47E-05	6
EGR1	0.724	1.48E-09	19	Cocaine	-0.443	6.80E-07	15	Bardoxolone	0.059	7.83E-05	8
**(2) Canonical Pathways**											
**Physical exercise vs. Control**				**MPTP vs. Control**				**MPTP + Physical exercise vs. MPTP**			
**Canonical pathway**	**z-score**	**p-value**	**#**	**Canonical pathway**	**z-score**	**p-value**	**#**	**Canonical pathway**	**z-score**	**p-value**	**#**
Dopamine receptor signaling	1.000	9.84E-04	11	Neuropathic pain signaling in dorsalhorn neurons	-2.714	2.87E-03	11	-			
VDR/RXR activation	-	9.84E-04	10	Cholecystokinin/gastrin-mediated signaling	-2.530	9.97E-03	10				
Neuropathic pain signaling in dorsalhorn neurons	-1.508	1.31E-03	11	Fcy receptor-mediated phagocytosis in macrophages and monocytes	-1.667	1.34E-02	9				
Axonal guidance signaling	-	5.16E-03	23	UVC-induced MAPK signaling	-2.449	1.34E-02	6				
Serotonin signaling	-	8.63E-03	7	VDR/RXR activation	-	1.34E-02	8				
**(3) Diseases and Disorders**											
**Physical exercise vs. Control**				**MPTP vs. Control**				**MPTP + Physical exercise vs. MPTP**			
**Annotation**	**z-score**	**p-value**	**#**	**Annotation**	**z-score**	**p-value**	**#**	**Annotation**	**z-score**	**p-value**	**#**
Movement disorders	-1.827	4.11E-15	88	Seizures	2.661	1.58E-10	41	Abdominal cancer	0.349	1.91E-03	395
Dyskinesia	-1.961	2.45E-13	60	Movement disorders	-0.599	1.06E-09	74	Cancer	0.794	1.96E-03	429
Neurological signs	-1.029	2.45E-13	62	Neurological signs	-0.600	1.06E-09	54	Abdominal neoplasm	0.593	2.18E-03	396
Disorder of basal ganglia	-2.621	1.64E-11	65	Dyskinesia	-	1.06E-09	52	Tumorigenesis	1.283	2.18E-03	406
Huntington's disease	-	1.47E-10	52	Huntington's disease	-	1.86E-09	49	Epithelial cancer	1.141	2.28E-03	399
**(4) Molecular and Cellular Functions**											
**Physical exercise vs. Control**				**MPTP vs. Control**				**MPTP + Physical exercise vs. MPTP**			
**Annotation**	**z-score**	**p-value**	**#**	**Annotation**	**z-score**	**p-value**	**#**	**Annotation**	**z-score**	**p-value**	**#**
Neurotransmission	-2.398	4.11E-15	51	Learning	-3.135	1.06E-09	41	-			
Cognition	-2.156	1.14E-12	49	Cognition	-3.183	1.20E-09	43				
Development of neurons	-2.001	8.80E-12	65	Development of neurons	-2.474	1.91E-07	54				
Learning	-2.159	9.41E-12	45	Synaptic depression	-2.193	8.46E-07	18				
Disorder of basal ganglia	-2.621	1.64E-11	65	Memory	-2.804	1.49E-06	26				

Supplementary Table 4. Enrichment analysis of mRNAs differentially expressed in the VTA. Ingenuity annotations of genes differentially expressed by running alone (CNR vs. CR; 1113 genes), MPTP alone (CNR vs. MNR; 1083 genes) and by running in MPTP-treated mice (MNR vs. MR; 741 genes). The top 10 upstream regulators, top 5 canonical pathways and top 5 annotations of the categories 'Diseases and disorders' and 'Molecular and Cellular Functions' are displayed, as well as their respective z-score, p-value and number of genes involved (#). All p-values are calculated using the Fisher's Exact Test, and the p-values for the canonical pathways and the categories 'Diseases and disorders' and 'Molecular and Cellular Functions' are corrected for multiple testing by the Benjamini-Hochberg correction. For the category 'Molecular and Cellular Functions', only the annotations with a significant z-score (i.e. <-2 (in red) or >2 (in green)) are shown. Annotations with only 1 (target) gene were discarded.

**(1) Upstream Regulators**											
**Physical exercise vs. Control**				**MPTP vs. Control**				**MPTP + Physical exercise vs. MPTP**			
**Upstream Regulator**	**z-score**	**p-value**	**#**	**Upstream Regulator**	**z-score**	**p-value**	**#**	**Upstream Regulator**	**z-score**	**p-value**	**#**
CREB1	1.731	6.71E-15	66	CREB1	2.414	4.16E-14	65	RICTOR	8.429	9.76E-53	75
HTT	-0.218	1.98E-12	73	MYCN	2.011	6.24E-12	38	ST1926	5.568	6.31E-17	120
ATN1	-	2.57E-12	28	L-dopa	-0.667	1.26E-11	70	KDM5A	5.099	6.75E-15	31
RICTOR	-3.479	5.41E-11	38	HTT	0.560	2.69E-11	71	HNF4A	-1.134	2.57E-14	31
Dopamine	1.365	2.22E-09	21	RICTOR	-5.088	7.94E-11	38	CD 437	5.204	4.93E-14	28
MYCN	2.397	1.61E-08	32	ATN1	-	6.43E-10	25	MYCN	-3.838	1.09E-11	52
APP	1.336	2.54E-08	72	VHL	-0.181	6.46E-10	24	5-fluorouracil	4.811	1.10E-10	19
SOD1	-	2.94E-08	27	HNF4A	1.708	2.35E-09	143	HTT	1	7.77E-10	37
L-dopa	-0.791	1.66E-07	59	Tretinoin	2.455	1.32E-07	103	interferon beta-1a		2.58E-09	12
EN1	-1.461	3.57E-07	7	HMGA1	-0.469	3.16E-07	22	sirolimus	4.980	6.00E-09	31
**(2) Canonical Pathways**											
**Physical exercise vs. Control**				**MPTP vs. Control**				**MPTP + Physical exercise vs. MPTP**			
**Canonical pathway**	**z-score**	**p-value**	**#**	**Canonical pathway**	**z-score**	**p-value**	**#**	**Canonical pathway**	**z-score**	**p-value**	**#**
EIF2 signaling	0.832	1.30E-07	31	mTOR signaling	-	9.34E-05	27	Oxidative phosphorylation	-	4.54E-26	38
mTOR signaling	-	1.25E-04	26	EIF2 signaling	3.464	9.34E-05	26	Mitochondrial dysfunction	-	2.07E-21	41
Dopamine receptor signaling	-	1.92E-03	15	Regulation of eIF4 and p70S6K signaling	-	2.37E-02	18	EIF2 signaling	-	5.83E-18	38
Regulation of eIF4 and p70S6K Signaling	-	4.48E-03	19	Oxidative phosphorylation	-	3.56E-02	15	Regulation of eIF4 and p70S6K signaling	-	1.09E-02	15
Mitochondrial dysfunction	-	9.32E-03	21								
**(3) Diseases and Disorders**											
**Physical exercise vs. Control**				**MPTP vs. Control**				**MPTP + Physical exercise vs. MPTP**			
**Annotation**	**z-score**	**p-value**	**#**	**Annotation**	**z-score**	**p-value**	**#**	**Annotation**	**z-score**	**p-value**	**#**
Seizure disorder	-2.654	1.44E-12	77	Dyskinesia	1.091	4.56E-08	85	Mitochondrial disorder	-	4.45E-09	25
Movement disorders	-0.639	1.72E-10	131	Movement Disorders	-0.814	4.56E-08	127	Mitochondrial respiratory chain deficiency	-	4.45E-09	17
Seizure	-2.543	9.74E-10	62	Disorder of basal ganglia	1.477	1.73E-07	98	Mitochondrial complex I deficiency	-	2.22E-06	11
Epilepsy	-1.890	1.46E-09	51	Neurological signs	0.928	1.73E-07	86	Neurological signs	-	5.47E-04	55
Neurological signs	0.926	1.85E-08	86	Neuromuscular disease	1.131	8.60E-07	106	Dyskinesia	-	6.75E-04	52
**(4) Molecular and Cellular Functions**											
**Physical exercise vs. Control**				**MPTP vs. Control**				**MPTP + Physical exercise vs. MPTP**			
**Annotation**	**z-score**	**p-value**	**#**	**Annotation**	**z-score**	**p-value**	**#**	**Annotation**	**z-score**	**p-value**	**#**
Development of central nervous system	2.819	1.32E-05	77	Size of body	5.539	5.45E-04	86	Cell death of osteosarcoma cells	5.099	3.10E-10	26
Size of body	4.217	5.07E-05	62	Release of neurotransmitter	-2.233	1.03E-03	27	Cell death of cancer cells	4.669	4.02E-02	32
Release of neurotransmitter	-2.522	1.66E-04	73	Morbidity or mortality	-6.049	2.09E-03	210				
Morbidity or mortality	-2.150	2.16E-03	88	Organismal death	-6.217	3.28E-03	206				
Organismaldeath	-2.261	2.67E-03	645	Secretion of neurotransmitter	-2.164	3.28E-03	22				

Supplementary Table 5. Enrichment analysis of mRNAs differentially expressed in the DL. Ingenuity annotations of genes differentially expressed by running alone (CNR vs. CR; 607 genes), MPTP alone (CNR vs. MNR; 245 genes) and by running in MPTP-treated mice (MNR vs. MR; 272 genes). The top 10 upstream regulators, top 5 canonical pathways and top 5 annotations of the categories 'Diseases and disorders' and 'Molecular and Cellular Functions' are displayed, as well as their respective z-score, p-value and number of genes involved (#). All p-values are calculated using the Fisher's Exact Test, and the p-values for the canonical pathways and the categories 'Diseases and disorders' and 'Molecular and Cellular Functions' are corrected for multiple testing by the Benjamini-Hochberg correction. For the category 'Molecular and Cellular Functions', only the annotations with a significant z-score (i.e. <-2 (in red) or >2 (in green)) are shown. Annotations with only 1 (target) gene were discarded.

(1) Upstream Regulators

Physical exercise vs. Control				MPTP vs. Control				MPTP + Physical exercise vs. MPTP			
**Upstream Regulator**	**z-score**	**p-value**	**#**	**Upstream Regulator**	**z-score**	**p-value**	**#**	**Upstream Regulator**	**z-score**	**p-value**	**#**
RICTOR	-7.230	1.72E-42	60	Dalfampridine	-3.317	6.20E-15	11	RICTOR	-2.191	6.91E-22	31
MYCN	2.809	2.42E-18	35	Bicuculline	-3.233	5.49E-14	11	HNF4A	-	4.46E-10	54
HNF4A	-1.463	1.91E-12	96	RICTOR	-4.472	1.52E-12	20	Sirolimus	-1.171	1.36E-07	20
Guanidinopropionic acid	3.308	9.81E-09	11	CREM	-2.844	2.36E-09	13	5-fluorouracil	-0.954	1.93E-07	14
MAP4K4	-3.873	4.69E-08	15	Cocaine	-3.472	6.60E-09	13	IGF1R	2.813	2.04E-07	14
INSR	4.094	5.61E-08	27	Kainic acid	-1.318	1.53E-08	12	MYCN	-0.898	6.61E-07	14
PSEN1	-1.035	6.17E-08	28	PSEN1	-1.755	4.24E-08	18	HTT	-	2.20E-06	24
MAPT	-	8.06E-08	22	MYCN	1.136	7.82E-07	13	INSR	2.157	1.48E-05	16
IGF1R	2.853	2.02E-07	20	CREB1	-2.521	1.16E-06	19	Guanidinopropionic acid	1.633	1.56E-05	6
RRP1B	-	4.25E-07	13	2-amino-5-phosphonovaleric acid	2.559	1.34E-06	10	CD 437	-0.302	2.48E-05	11

(2) Canonical Pathways

Physical exercise vs. Control				MPTP vs. Control				MPTP + Physical exercise vs. MPTP			
**Canonical pathway**	**z-score**	**p-value**	**#**	**Canonical pathway**	**z-score**	**p-value**	**#**	**Canonical pathway**	**z-score**	**p-value**	**#**
Oxidative phosphorylation	-	3.46E-20	30	Oxidative phosphorylation	-	1.00E-04	10	Oxidative phosphorylation	-	1.91E-08	14
Mitochondrial dysfunction	-	1.93E-15	31	EIF2 signaling	2.333	4.78E-04	11	Mitochondrial dysfunction	-	3.91E-06	14
EIF2 signaling	3.771	1.95E-09	24	Mitochondrial dysfunction	-	2.06E-03	10	EIF2 signaling	-0.816	3.32E-05	13
								mTOR signaling	-	7.31E-03	10
								Regulation of eIF4 and p70S6K signaling	-	2.48E-02	8

(3) Diseases and Disorders

Physical exercise vs. Control				MPTP vs. Control				MPTP + Physical exercise vs. MPTP			
**Annotation**	**z-score**	**p-value**	**#**	**Annotation**	**z-score**	**p-value**	**#**	**Annotation**	**z-score**	**p-value**	**#**
Mitochondrial disorder	-	1.84E-02	14	Epileptic seizure	-	2.07E-07	16	Cell death of osteosarcoma cells	-	1.05E-02	10
Mitochondrial respiratory chain deficiency	-	1.84E-02	9	Epilepsy	-	3.29E-04	17	Mitochondrial respiratory chain deficiency	-	1.31E-02	7
Diamond-Blackfananemia	-	1.84E-02	5	Congenital anemia	-	3.29E-04	8	Mitochondrial complex I deficiency	-	2.49E-02	5
Congenital anemia	-	2.65E-02	9	Seizures	-	3.09E-03	18	Mitochondrial disorder	-	3.12E-02	9
				Congenital aplastic anemia	-	3.09E-03	6	Unstable hemoglobin disease	-	4.75E-02	2

(4) Molecular and Cellular Functions

Physical exercise vs. Control				MPTP vs. Control				MPTP + Physical exercise vs. MPTP			
**Annotation**	**z-score**	**p-value**	**#**	**Annotation**	**z-score**	**p-value**	**#**	**Annotation**	**z-score**	**p-value**	**#**
-				-				-			

Supplementary Table 6. Enrichment analysis of mRNAs differentially expressed in the VM. Ingenuity annotations of genes differentially expressed by running alone (CNR vs. CR; 304 genes), MPTP alone (CNR vs. MNR; 114 genes) and by running in MPTP-treated mice (MNR vs. MR; 949 genes). The top 10 upstream regulators, top 5 canonical pathways and top 5 annotations of the categories 'Diseases and disorders' and 'Molecular and Cellular Functions' are displayed, as well as their respective z-score, p-value and number of genes involved (#). All p-values are calculated using the Fisher's Exact Test, and the p-values for the canonical pathways and the categories 'Diseases and disorders' and 'Molecular and Cellular Functions' are corrected for multiple testing by the Benjamini-Hochberg correction. For the category 'Molecular and Cellular Functions', only the annotations with a significant z-score (i.e. <-2 (in red) or >2 (in green)) are shown. Annotations with only 1 (target) gene were discarded.

**(1) Upstream Regulators**											
**Physical exercise vs. Control**				**MPTP vs. Control**				**MPTP + Physical exercise vs. MPTP**			
**Upstream Regulator**	**z-score**	**p-value**	**#**	**Upstream Regulator**	**z-score**	**p-value**	**#**	**Upstream Regulator**	**z-score**	**p-value**	**#**
L-dopa	-1.460	7.09E-06	23	CREB1	-1.066	1.31E-07	14	L-dopa	-8.463	2.28E-45	118
Alpha-amanitin	-	1.83E-05	4	Dalfampridine	-2.236	3.25E-07	5	HTT	1.820	4.23E-23	87
APP	1.963	3.38E-05	25	Bicuculline	-2.177	7.92E-07	5	CREB1	-0.960	3.27E-22	74
EPHB2	-	1.35E-04	3	Trichostatin A	0.864	1.58E-06	13	ATN1	-	3.69E-16	31
CREB1	-0.117	2.38E-04	17	Alpha-amanitin	-	3.22E-05	3	Beta-estradiol	1.885	1.26E-15	135
ADRB	-1.480	2.65E-04	8	2-amino-5-phosphonovaleric acid	0.896	4.79E-05	6	BDNF	2.971	3.25E-15	45
MYOD1	1.450	4.08E-04	9	Forskolin	-1.422	1.95E-04	11	Dopamine	0.076	9.48E-15	26
HIF1A	-1.009	4.70E-04	13	G2535	-	2.03E-04	2	MAPT	-0.896	1.05E-13	39
KLF1	-	6.25E-04	4	N-lauroyl-L-phenylalanine	-	2.03E-04	2	APP	0.170	7.12E-12	74
SIGMAR1	-	8.43E-04	2	Lactacystin	-0.342	2.51E-04	6	REST	-2.148	2.02E-10	23
**(2) Canonical Pathways**											
**Physical exercise vs. Control**				**MPTP vs. Control**				**MPTP + Physical exercise vs. MPTP**			
**Canonical pathway**	**z-score**	**p-value**	**#**	**Canonical pathway**	**z-score**	**p-value**	**#**	**Canonical pathway**	**z-score**	**p-value**	**#**
-				-				Gai Signaling	-2.000	6.37E-08	24
								Breast cancer regulation by Stathmin1	-	1.05E-07	31
								cAMP-mediated signaling	-0.365	1.10E-07	32
								Role of NFAT in cardiac hypertrophy	2.558	6.48E-05	25
								G-Protein Coupled Receptor Signaling	-	9.00E-05	23
**(3) Diseases and Disorders**											
**Physical exercise vs. Control**				**MPTP vs. Control**				**MPTP + Physical exercise vs. MPTP**			
**Annotation**	**z-score**	**p-value**	**#**	**Annotation**	**z-score**	**p-value**	**#**	**Annotation**	**z-score**	**p-value**	**#**
-				Epilepsy	-	6.30E-05	13	Movement disorders	-2.334	1.57E-28	169
				Epileptic seizure	-	2.52E-03	8	Disorder of basal ganglia	0.415	1.49E-24	129
				Seizures	1.172	3.16E-03	12	Seizure disorder	-2.791	4.66E-23	93
				Unstable hemoglobin disease	-	7.81E-03	2	Seizures	-2.841	3.00E-22	81
				Alphathalassemia	-	1.40E-02	2	Neuromuscular disease	0.314	9.11E-22	135
**(4) Molecular and Cellular Functions**											
**Physical exercise vs. Control**				**MPTP vs. Control**				**MPTP + Physical exercise vs. MPTP**			
**Annotation**	**z-score**	**p-value**	**#**	**Annotation**	**z-score**	**p-value**	**#**	**Annotation**	**z-score**	**p-value**	**#**
-				-				Microtubule dynamics	2.824	2.94E-21	164
								Organization of cytoskeleton	2.640	5.54E-21	106
								Organization of cytoplasm	2.640	9.30E-21	103
								Learning	2.055	1.44E-14	72
								Quantity of neurons	2.680	1.89E-10	62

Supplementary Table 7. Enrichment analysis of mRNAs differentially expressed in the PFC. Ingenuity annotations of genes differentially expressed by running alone (CNR vs. CR; 367 genes), MPTP alone (CNR vs. MNR; 378 genes) and by running in MPTP-treated mice (MNR vs. MR; 331 genes). The top 10 upstream regulators, top 5 canonical pathways and top 5 annotations of the categories 'Diseases and disorders' and 'Molecular and Cellular Functions' are displayed, as well as their respective z-score, p-value and number of genes involved (#).All p-values are calculated using the Fisher's Exact Test, and the p-values for the canonical pathways and the categories 'Diseases and disorders' and 'Molecular and Cellular Functions' are corrected for multiple testing by the Benjamini-Hochberg correction. For the category 'Molecular and Cellular Functions', only the annotations with a significant z-score (i.e. <-2 (in red) or >2 (in green)) are shown. Annotations with only 1 (target) gene were discarded.

(1) Upstream Regulators

Physical exercise vs. Control				MPTP vs. Control				MPTP + Physical exercise vs. MPTP			
**Upstream Regulator**	**z-score**	**p-value**	**#**	**Upstream Regulator**	**z-score**	**p-value**	**#**	**Upstream Regulator**	**z-score**	**p-value**	**#**
Dexamethasone	-2.765	9.42E-12	58	Dalfampridine	-3.464	2.06E-14	12	Cycloheximide	1.398	6.15E-10	23
MYC	-1.471	1.65E-10	42	Bicuculline	-3.384	2.28E-13	12	Dexamethasone	0.679	1.43E-09	54
HRAS	-0.616	1.53E-09	29	CREB1	-3.969	1.15E-08	28	Bicuculline	2.946	1.67E-09	9
KRAS	2.829	1.71E-08	21	Pyridaben	3.000	1.29E-07	9	Dalfampridine	2.828	6.90E-09	8
TGFB1	-1.697	3.10E-08	51	Maneb	-2.256	4.26E-07	13	HRAS	0.425	1.22E-08	27
RICTOR	3.769	3.11E-08	18	6,7-dinitroquinoxaline-2,3-dione	3.000	4.32E-07	9	HTT	2.391	1.91E-08	30
Methylprednisolone	-0.652	3.33E-08	26	GnRH-A	-1.732	7.54E-07	4	TGFB1	0.708	2.33E-08	50
Tretinoin	-2.546	5.18E-08	46	Kainic acid	-2.946	1.38E-06	9	F2	1.657	5.06E-08	17
BMP7	-1.307	1.32E-07	12	Atipamezole	-2.901	1.79E-06	12	Atorvastatin	2.339	8.39E-08	15
SMARCA4	-2.892	1.43E-07	26	2-amino-3-phosphonopropionic acid	-	3.43E-06	3	N-lauroyl-L-phenylalanine	0	1.49E-07	4

(2) Canonical Pathways

Physical exercise vs. Control				MPTP vs. Control				MPTP + Physical exercise vs. MPTP			
**Canonical pathway**	**z-score**	**p-value**	**#**	**Canonical pathway**	**z-score**	**p-value**	**#**	**Canonical pathway**	**z-score**	**p-value**	**#**
-				-				RAR Activation	-	4.59E-02	11

(3) Diseases and Disorders

Physical exercise vs. Control				MPTP vs. Control				MPTP + Physical exercise vs. MPTP			
**Annotation**	**z-score**	**p-value**	**#**	**Annotation**	**z-score**	**p-value**	**#**	**Annotation**	**z-score**	**p-value**	**#**
Advanced malignant tumor	-1.854	2.87E-04	41	Epileptic seizures	-	6.87E-08	20	Cancer	0.101	9.84E-04	280
Metastasis	-1.854	4.77E-03	33	Epilepsy	0.625	1.46E-07	27	Malignant solid tumor	0.377	9.84E-04	276
Hypersensitive reaction	-2.214	4.77E-03	22	Seizures	-0.575	7.08E-05	27	Epilepsy	-	9.84E-04	21
Quantity of phagocytes	-0.418	4.77E-03	22	Seizure disorder	-0.587	1.87E-04	29	Hypersensitive reaction	1.858	9.84E-04	19
Infarction	0.444	4.77E-03	20	Alphathalassemia	-	3.02E-03	3	Tumorigenesis of tissue	0.158	1.81E-03	264

(4) Molecular and Cellular Functions

Physical exercise vs. Control				MPTP vs. Control				MPTP + Physical exercise vs. MPTP			
**Annotation**	**z-score**	**p-value**	**#**	**Annotation**	**z-score**	**p-value**	**#**	**Annotation**	**z-score**	**p-value**	**#**
Proliferation of cells	-3.071	1.17E-03	118	-				Morbidity or mortality	-2.177	1.17E-02	79
Morbidity or mortality	5.342	4.77E-03	81					Organismal death	-2.299	1.19E-02	78
Vasculogenesis	-2.127	4.77E-03	34					Development of abdomen	2.170	1.34E-02	26
Organismal death	5.577	4.77E-03	79					Development of epithelial tissue	2.009	2.32E-02	23
Migration of endothelial cells	-2.625	4.77E-03	19					Neuronal cell death	-2.067	2.33E-02	29

Supplementary Table 8. Enrichment analysis of mRNAs differentially expressed in the PPN. Ingenuity annotations of genes differentially expressed by running alone (CNR vs. CR; 501 genes), MPTP alone (CNR vs. MNR; 233 genes) and by running in MPTP-treated mice (MNR vs. MR; 255 genes). The top 10 upstream regulators, top 5 canonical pathways and top 5 annotations of the categories 'Diseases and disorders' and 'Molecular and Cellular Functions' are displayed, as well as their respective z-score, p-value and number of genes involved (#). All p-values are calculated using the Fisher's Exact Test, and the p-values for the canonical pathways and the categories 'Diseases and disorders' and 'Molecular and Cellular Functions' are corrected for multiple testing by the Benjamini-Hochberg correction. For the category 'Molecular and Cellular Functions', only the annotations with a significant z-score (i.e. <-2 (in red) or >2 (in green)) are shown. Annotations with only 1 (target) gene were discarded.

(1) Upstream Regulators

Physical exercise vs. Control				MPTP vs. Control				MPTP + Physical exercise vs. MPTP			
**Upstream Regulator**	**z-score**	**p-value**	**#**	**Upstream Regulator**	**z-score**	**p-value**	**#**	**Upstream Regulator**	**z-score**	**p-value**	**#**
L-dopa	-1.820	2.50E-17	53	L-dopa	-0.525	1.75E-10	27	CREB1	0.510	2.20E-06	19
HTT	1.858	5.48E-16	52	Amphetamine	-1.249	4.49E-10	11	Haloperidol	-0.166	2.61E-06	8
CREB1	-0.435	1.05E-14	43	GDNF	-0.049	5.10E-10	10	HU-210	-	1.00E-05	3
Beta-estradiol	-2.207	3.66E-10	75	Alpha-amanitin	1.387	1.67E-09	6	PRKAA2	0.194	1.53E-05	8
BDNF	-2.024	4.23E-10	26	K+	-2.023	2.30E-09	8	NGF	0.671	1.73E-05	10
ATN1	-	1.90E-09	17	BMP2	-2.019	3.48E-09	14	Clozapine	0.186	3.49E-05	6
REST	0.693	2.63E-08	15	Quinolinic acid	-0.538	6.36E-09	8	Mek	1.793	4.39E-05	9
NGF	-1.675	3.44E-08	18	Pargyline	-	7.97E-09	5	Risperidone	-	6.44E-05	4
ADCYAP1	-1.070	7.53E-08	20	HTT	0.623	8.79E-09	25	Cadmium	0.816	9.67E-05	6
Amphetamine	-2.756	1.60E-07	12	Beta-estradiol	-1.262	9.11E-09	42	HSD17B13	-	1.02E-04	2

(2) Canonical Pathways

Physical exercise vs. Control				MPTP vs. Control				MPTP + Physical exercise vs. MPTP			
**Canonical pathway**	**z-score**	**p-value**	**#**	**Canonical pathway**	**z-score**	**p-value**	**#**	**Canonical pathway**	**z-score**	**p-value**	**#**
Axonal guidance signaling	-	3.92E-02	23	Dopamine receptor signaling	-1.000	8.13E-04	8	-			
				Dopamine-DARPP32 Feedback in cAMP signaling	-0.816	7.05E-03	9				
				Glutamate receptor signaling	-	3.29E-02	5				
				Breast cancer regulation by stathmin1	-	4.45E-02	8				

(3) Diseases and Disorders

Physical exercise vs. Control				MPTP vs. Control				MPTP + Physical exercise vs. MPTP			
**Annotation**	**z-score**	**p-value**	**#**	**Annotation**	**z-score**	**p-value**	**#**	**Annotation**	**z-score**	**p-value**	**#**
Neurological signs	1.091	6.74E-08	54	Purkinje cell degeneration	-	1.51E-07	8	Methemoglobinemia	-	4.23E-02	3
Movement disorders	1.806	1.64E-07	73	Epileptic seizure	-	1.05E-05	14				
Dyskinesia	-	2.45E-07	50	Epilepsy	-	5.03E-05	18				
Disorder of basal ganglia	-	2.98E-07	58	Neurological signs	0.600	1.98E-04	27				
Huntington's disease	-	4.07E-07	47	Seizures	-0.798	3.30E-04	19				

(4) Molecular and Cellular Functions

Physical exercise vs. Control				MPTP vs. Control				MPTP + Physical exercise vs. MPTP			
**Annotation**	**z-score**	**p-value**	**#**	**Annotation**	**z-score**	**p-value**	**#**	**Annotation**	**z-score**	**p-value**	**#**
Development of neurons	-2.394	6.74E-08	60	Cognition	-3.400	1.58E-04	22	-			
Formation of cellular protrusions	-2.172	2.70E-06	60	Learning	-2.940	3.30E-04	20				
Microtubule dynamics	-2.272	2.59E-05	70	Transport of molecule	-3.275	1.03E-02	42				
Coordination	-3.490	3.96E-05	21	Release of neurotransmitter	-2.333	1.86E-02	9				
Organismal death	4.315	1.32E-03	111	Differentiation of cells	-2.082	3.48E-02	49				

**Supplementary Table 9.** Differentially expressed mRNAs in the SN due to physical exercise in MPTP-treated mice, *and* regulated by L-DOPA. Proteins encoded by the genes are **bold** when located in the molecular landscape (**Figure 4**) and single-underlined when associated to PD via 'corroborating evidence', i.e. when the gene/protein is genetically associated to PD or differentially expressed in the SN of PD patients compared to controls.			
Gene	Effect on mRNA expression by		Corroborating evidence
	Physical Exercise	L-DOPA	
**ACKR1**	-1.24	Increased	-
**ACTA2**	-1.27	Decreased	-
**ALYREF**	1.26	Increased	-
**ARRB2**	-1.23	Decreased	-
C14ORF166	1.21	Decreased	-
CASKIN2	-1.20	Increased	-
**CDC42EP2**	-1.23	Decreased	CDC42EP2 mRNA is increased in the SN of PD patients compared to controls[2].
**CDC42EP3**	1.29	Increased	-
**CIART**	-1.34	Increased	-
CLN6	-1.31	Decreased	-
**DBP**	-1.20	Increased	-
DGKB	1.31	Increased	DGKB mRNA is decreased in the SN of PD patients compared to controls[2].
DPP7	-1.20	Decreased	-
**DUSP6**	-1.22	Increased	-
**EDNRB**	1.21	Decreased	-
FCRLS	-1.26	Decreased	-
**FKBP5**	-1.29	Increased	-
**FTL**	-1.24	Decreased	FTL mRNA is decreased in the SN of PD patients compared to controls[3]. FTL protein is increased in the SN of PD patients compared to controls[4, 5].
GDF1	-1.21	Increased	GDF1 mRNA is decreased in the SN of PD patients compared to controls[6].
**GPR39**	-1.26	Increased	-
**HSPB6**	-1.24	Decreased	HSPB6 protein is increased in the SN of PD patients compared to controls[4].
HVCN1	-1.22	Decreased	-
**IER3**	-1.22	Increased	-
INO80E	-1.38	Increased	-
**MFSD2A**	-1.34	Increased	-
MRPL33	1.36	Increased	-
**PDGFA**	-1.22	Decreased	-
**PER1**	-1.24	Increased	-
**PPP1R1B**	-1.21	Increased	PPP1R1B protein is decreased by 66% in the SNpr, and 79% in the SNpc of PD patients compared to controls[7].
**PVALB**	-1.21	Decreased	PVALB mRNA is increased in the SN of PD patients compared to controls[8]. PVALB protein is increased in a subgroup of PD DA SN neurons[9].
REEP3	1.21	Decreased	-
**ROMO1**	-1.29	Increased	-
**SHC1**	-1.24	Decreased	-
**SLC2A1**	-1.22	Increased	-
**TAC1**	-1.31	Increased	TAC1 mRNA is decreased in the SN of PD patients compared to controls[8].
**TP53**	-1.24	Increased	Ser15-phosphorylated TP53 is increased in the PD SN (p<0.001)[10].

Supplementary Table 10. Differentially expressed mRNAs in the VM due to physical exercise in MPTP-treated mice, *and* regulated by L-DOPA. Proteins encoded by the genes are **bold** when located in the molecular landscape (**Figure 5**) and single-underlined when associated to PD via 'corroborating evidence', i.e. when the gene/protein is genetically associated to PD or differentially expressed in the striatum of PD patients compared to controls.

Gene	Effect on mRNA expression by		Corroborating evidence
	Physical Exercise	L-DOPA	
ACY1	-1.2	Increased	-
**AMIGO2**	-1.46	Increased	-
**APBA2**	1.28	Decreased	-
**ARC**	1.25	Increased	ARC mRNA is decreased in the striatum of PD patients compared to controls[8].
**ARPP21**	-1.2	Increased	ARPP21 is decreased in the striatum of PD patients compared to controls[11].
C14orf37	1.28	Decreased	-
C18orf21	-1.47	Increased	-
C4A/C4B	-1.32	Decreased	-
**CACNA2D3**	-1.23	Increased	(VDCC) CACNA2D3 mRNA is decreased in the striatum of PD patients compared to controls[12].
**CAMK1G**	-1.49	Increased	CAMK1G mRNA is increased in the striatum of PD patients compared to controls[13].
**CBR3**	-1.37	Increased	CBR3 protein is increased in the striatum of PD patients compared to controls[8].
**CCND1**	1.2	Decreased	-
**CDC42EP3**	-1.29	Increased	-
**CHAT**	-1.58	Increased	-
**CHRNA4**	1.92	Decreased	PD patients have a higher frequency of intron3+182 Del 22 bp (p=0.015)[14].
**CLIC6**	-1.27	Increased	CLIC6 mRNA is increased in the striatum of PD patients compared to controls[8].
CLN6	1.31	Decreased	-
CNIH3	2.8	Decreased	-
**CNR1**	1.49	Decreased	The presence of two CNR1 alleles with >16 AAT trinucleotide repeats in PD patients, is associated with a reduced prevalence of depression (p=0.003)[15]. CNR1 mRNA is increased in the striatum of PD patients compared to controls[13].
CRTAC1	1.79	Decreased	-
**CRYM**	-1.41	Increased	CRYM protein is increased in the striatum of PD patients compared to controls[8].
**CTNNBIP1**	1.31	Decreased	-
**CYLD**	-1.24	Increased	-
DDIT4L	-1.27	Increased	-
DGKB	-1.2	Increased	-
**DGKI**	-1.27	Increased	-
DLGAP1	1.73	Decreased	-
**DLK1**	-1.54	Increased	-
**DOC2B**	-1.3	Increased	-
**DUSP6**	1.24	Increased	-
**ECEL1**	-1.41	Increased	-
ERLIN1	-1.24	Increased	-
**FAAH**	2.15	Decreased	The synonymous rs324419 SNP and also the haplotype of rs324419 and rs2295633 (both SNPs are located in the FAAH gene) are both associated with musculoskeletal pain in PD patients p=0.006 and p=0.012 respectively[16].
FAM126A	-1.25	Decreased	-
FAM184b	-1.28	Increased	-
FAM81A	2.1	Decreased	-
FDPS	1.47	Increased	-
FNDC9	-1.3	Increased	-
FOXP1	-1.21	Increased	-
**GABRA3**	1.43	Decreased	GABRA3 mRNA is increased in the striatum of PD patients compared to

			controls[8].
**<u>GABRG2</u>**	1.24	Decreased	GABRG2 mRNA is increased in the striatum of PD patients compared to controls[13].
**<u>GFAP</u>**	1.22	Decreased	GFAP mRNA and protein is increased in the striatum of PD patients compared to controls[8].
**GNAS**	1.55	Decreased	-
GNG7	-1.22	Increased	-
<u>GOLIM4</u>	-1.26	Decreased	GOLIM4 mRNA is increased in the striatum of PD patients compared to controls[8].
**<u>GPD2</u>**	1.24	Decreased	GPD2 mRNA is increased in the striatum of PD patients compared to controls[8].
**GUCY1A3**	-1.33	Increased	-
HAPLN4	3.14	Decreased	-
**HBEGF**	-1.24	Increased	-
**HLA-A**	-1.24	Decreased	-
**HTR1B**	-1.32	Increased	-
IDO1	-1.35	Increased	-
**KCNA5**	-1.28	Increased	-
**<u>KCNA6</u>**	1.65	Decreased	KCNA6 mRNA is increased in the striatum of PD patients compared to controls[8].
**KCNC4**	4.15	Decreased	-
**KCNE5**	-1.32	Decreased	-
**KLF6**	1.59	Decreased	-
**LMNA**	1.23	Decreased	-
LPPR1	-1.28	Increased	-
**LRRTM3**	-1.26	Increased	-
LYPD1	-1.22	Increased	-
LZTS3	-1.2	Increased	-
**MAPK11**	2.9	Decreased	-
**<u>MBP</u>**	1.22	Increased	MBP mRNA is decreased in the striatum of PD patients compared to controls[12]. MBP protein is decreased in the striatum of PD patients compared to controls[8].
**<u>MEF2C</u>**	1.29	Decreased	MEF2C mRNA is increased in the striatum of PD patients compared to controls[8].
MPP6	-1.33	Increased	-
<u>MSMO1</u>	1.24	Increased	MSMO1 mRNA is increased in the striatum of PD patients compared to controls[13].
MTURN	1.26	Decreased	-
**MYO1B**	-1.29	Increased	-
NAV1	1.31	Decreased	-
**<u>NPTX2</u>**	1.27	Increased	NPTX2 mRNA is decreased in the striatum of PD patients compared to controls[12].
**NUAK1**	1.24	Decreased	-
PARM1	1.53	Decreased	-
**<u>PDYN</u>**	-1.49	Increased	PDYN mRNA is increased in the striatum of PD patients compared to controls[8].
PDZD2	-1.32	Increased	-
**<u>PENK</u>**	-1.2	Increased	PENK mRNA is increased in the striatum of PD patients compared to controls[8].
**<u>PGM2L1</u>**	1.37	Decreased	PGM2L1 mRNA is increased in the striatum of PD patients compared to controls[13].
PLEKHA2	1.35	Decreased	-
PPM1L	1.68	Decreased	-
**<u>PPP1R1B</u>**	-1.2	Increased	PPP1R1B protein is decreased by 45% in the striatum of PD patients compared to controls[7].
**PPP1R2**	-1.24	Increased	-
PRICKLE1	1.55	Decreased	-
**PTPRD**	1.26	Decreased	-
**PVALB**	4.27	Decreased	-
**RAP1GAP**	-1.2	Increased	-
RAPGEFL1	1.24	Decreased	-

**REM2**	-1.23	Increased	-
**RIMS1**	1.52	Decreased	-
ROBO2	1.22	Decreased	-
SATB1	2.73	Decreased	-
SCN4B	-1.2	Increased	SCN4B protein is increased in the striatum of PD patients compared to controls[8].
SCUBE3	-1.25	Increased	-
**SH3GL2**	1.3	Decreased	SH3GL2 mRNA is increased in the striatum of PD patients compared to controls[13].
SLC10A4	-1.56	Increased	-
**SLC24A2**	1.61	Decreased	-
SLC2A6	1.68	Decreased	-
SLC6A7	1.94	Decreased	-
SMUG1	1.21	Increased	-
**SNCB**	1.84	Decreased	SNCB mRNA is decreased in the striatum of PD patients compared to controls[17].
SOBP	1.83	Decreased	-
**STRN**	-1.27	Increased	-
**STXBP1**	1.2	Decreased	STXBP1 mRNA is decreased in the striatum of PD patients compared to controls[17].
**SYN1**	1.3	Decreased	SYN1 mRNA is decreased in the striatum of PD patients compared to controls[17]. SYN1 protein is increased in the striatum of PD patients compared to controls[8].
SYNDIG1L	-1.27	Increased	-
**TAC1**	-1.23	Increased	TAC1 mRNA is increased[8, 13], or decreased[11] in the striatum of PD patients compared to controls.
**TCF4**	1.25	Decreased	-
TCIRG1	1.34	Decreased	-
**TH**	-1.33	Increased	TH mRNA and protein is decreased in the terminals of nigro-striatal DA neurons of PD patients[18, 19].
**TLE1**	-1.21	Increased	-
TMEM141	1.55	Decreased	-
**TRPC3**	-1.49	Increased	-
**UNC13C**	-1.37	Increased	-
**VAMP1**	1.59	Decreased	(SNARE)
**VAT1L**	-1.32	Increased	VAT1L mRNA is increased in the striatum of PD patients compared to controls[13]. VAT1L protein is increased in the striatum of PD patients compared to controls[8].
WDR17	-1.23	Increased	WDR17 mRNA is increased in the striatum of PD patients compared to controls[13].
**WNT7B**	2.27	Decreased	-
ZCCHC12	-1.23	Increased	-
**ZEB2**	1.62	Decreased	-

Supplementary Table 11. Differentially expressed mRNAs in the DL due to physical exercise in MPTP-treated mice, *and* regulated by RICTOR. If the proteins that are encoded by these mRNAs are a subunit of a bigger protein complex, then the name of the protein complex is shown in the column 'part of'.

Gene	Effect on mRNA expression by		Part of[20]:
	Physical Exercise	RICTOR	
ATP5E	1.39	Decreased	Mitochondrial complex V
ATP5H	1.28	Decreased	Mitochondrial complex V
ATP5L	1.54	Decreased	Mitochondrial complex V
COX6B1	1.36	Decreased	Mitochondrial complex IV
COX7C	1.21	Decreased	Mitochondrial complex IV
NDUFA1	1.33	Decreased	Mitochondrial complex I
NDUFA11	1.24	Decreased	Mitochondrial complex I
NDUFA7	1.25	Decreased	Mitochondrial complex I
NDUFA9	1.23	Decreased	Mitochondrial complex I
NDUFB4	1.56	Decreased	Mitochondrial complex I
NDUFB9	1.22	Decreased	Mitochondrial complex I
NDUFS5	1.36	Decreased	Mitochondrial complex I
NDUFV3	1.22	Decreased	Mitochondrial complex I
PSMA2	1.23	Decreased	20S proteasome subunit α
PSMB6	-1.3	Decreased	20S proteasome subunit β
PSME1	1.28	Decreased	Proteasome activator complex
RPL10	-1.34	Decreased	60S ribosome subunit
RPL17	1.26	Decreased	60S ribosome subunit
RPL28	-1.61	Decreased	60S ribosome subunit
RPL29	-1.37	Decreased	60S ribosome subunit
RPL7A	-1.21	Decreased	60S ribosome subunit
RPL9	-1.34	Decreased	60S ribosome subunit
RPS15	-1.22	Decreased	40S ribosome subunit
RPS24	-1.25	Decreased	40S ribosome subunit
RPS26	1.23	Decreased	40S ribosome subunit
RPS27A	1.31	Decreased	40S ribosome subunit
RPS29	1.79	Decreased	40S ribosome subunit
SDHB	1.24	Decreased	Mitochondrial complex II
SGK1	-1.46	Decreased	-
UQCRQ	1.2	Decreased	Mitochondrial complex III
VCAM1	1.25	Increased	-

**Supplementary Table 12.** Differentially expressed mRNAs in the VTA due to physical exercise in MPTP-treated mice, *and* regulated by RICTOR. If the proteins that are encoded by these mRNAs are a subunit of a bigger protein complex, then the name of the protein complex is shown in the column 'part of'.			
Gene	Effect on mRNA expression by		Part of[20]:
	Physical Exercise	RICTOR	
ATP5E	-1.64	Decreased	Mitochondrial complex V
ATP5F1	-1.29	Decreased	Mitochondrial complex V
ATP5G1	-1.42	Decreased	Mitochondrial complex V
ATP5G2	-1.28	Decreased	Mitochondrial complex V
ATP5H	-1.25	Decreased	Mitochondrial complex V
ATP5J	-1.25	Decreased	Mitochondrial complex V
ATP5J2	-1.29	Decreased	Mitochondrial complex V
COX17	-1.35	Decreased	Mitochondrial complex IV
COX5A	-1.28	Decreased	Mitochondrial complex IV
COX5B	-1.33	Decreased	Mitochondrial complex IV
COX6B1	-1.47	Decreased	Mitochondrial complex IV
COX6C	-1.45	Decreased	Mitochondrial complex IV
COX7A2	-1.22	Decreased	Mitochondrial complex IV
COX7B	-1.25	Decreased	Mitochondrial complex IV
COX7C	-1.25	Decreased	Mitochondrial complex IV
MRPL13	-1.3	Decreased	39S ribosome subunit (mitochondrial)
NDUFA1	-1.58	Decreased	Mitochondrial complex I
NDUFA2	-1.24	Decreased	Mitochondrial complex I
NDUFA3	-1.33	Decreased	Mitochondrial complex I
NDUFA4	-1.32	Decreased	Mitochondrial complex IV
NDUFA7	-1.37	Decreased	Mitochondrial complex I
NDUFA9	-1.22	Decreased	Mitochondrial complex I
NDUFB10	-1.22	Decreased	Mitochondrial complex I
NDUFB2	-1.32	Decreased	Mitochondrial complex I
NDUFB4	-1.22	Decreased	Mitochondrial complex I
NDUFB5	-1.36	Decreased	Mitochondrial complex I
NDUFB6	-1.25	Decreased	Mitochondrial complex I
NDUFB7	-1.22	Decreased	Mitochondrial complex I
NDUFB8	-1.23	Decreased	Mitochondrial complex I
NDUFB9	-1.28	Decreased	Mitochondrial complex I
NDUFC1	1.23	Decreased	Mitochondrial complex I
NDUFS4	-1.26	Decreased	Mitochondrial complex I
NDUFS5	-1.31	Decreased	Mitochondrial complex I
NDUFS6	-1.42	Decreased	Mitochondrial complex I
NDUFV2	-1.21	Decreased	Mitochondrial complex I
NDUFV3	-1.22	Decreased	Mitochondrial complex I
POMP	-1.37	Decreased	Proteasome
PPA2	-1.24	Decreased	-
PSMA4	-1.24	Decreased	20S proteasome subunit α
PSMA5	-1.32	Decreased	20S proteasome subunit α
PSMA6	-1.3	Decreased	20S proteasome subunit α
PSMB1	-1.27	Decreased	20S proteasome subunit β
PSMB2	-1.31	Decreased	20S proteasome subunit β
PSMB3	-1.35	Decreased	20S proteasome subunit β
PSMB5	-1.23	Decreased	20S proteasome subunit β
PSMB7	-1.24	Decreased	20S proteasome subunit β
PSME1	-1.2	Decreased	Proteasome activator complex
RPL11	-1.38	Decreased	60S ribosome subunit
RPL12	-1.35	Decreased	60S ribosome subunit
RPL17	-1.5	Decreased	60S ribosome subunit
RPL21	-1.2	Decreased	60S ribosome subunit
RPL23A	-1.3	Decreased	60S ribosome subunit
RPL26	-1.41	Decreased	60S ribosome subunit
RPL29	-1.29	Decreased	60S ribosome subunit

RPL30	-1.43	Decreased	60S ribosome subunit
RPL34	-1.44	Decreased	60S ribosome subunit
RPL35A	-1.35	Decreased	60S ribosome subunit
RPL38	-1.69	Decreased	60S ribosome subunit
RPL41	-1.26	Decreased	60S ribosome subunit
RPLP1	-1.25	Decreased	60S ribosome subunit
RPLP2	-1.52	Decreased	60S ribosome subunit
RPS10	-1.2	Decreased	40S ribosome subunit
RPS15	-1.32	Decreased	40S ribosome subunit
RPS18	-1.25	Decreased	40S ribosome subunit
RPS29	-1.49	Decreased	40S ribosome subunit
RPS3A1	-1.2	Decreased	40S ribosome subunit
RPS4Y1	-1.36	Decreased	40S ribosome subunit
SDHB	-1.22	Decreased	Mitochondrial complex II
SGK1	-1.62	Decreased	-
SHFM1	-1.43	Decreased	26S proteasome
UBA52	-1.33	Decreased	60S ribosome subunit
UQCR10	-1.21	Decreased	Mitochondrial complex III
UQCRB	-1.23	Decreased	Mitochondrial complex III
UQCRHL	-1.36	Decreased	Mitochondrial complex III
UQCRQ	-1.25	Decreased	Mitochondrial complex III

**Supplementary Table 13.** Differentially expressed mRNAs in the PFC due to physical exercise in MPTP-treated mice, *and* regulated by Bicuculline and Dalfampridine. For each gene/mRNA their association to epilepsy and/or seizures is shown in the column 'Association to epilepsy / seizures'.			
Gene	Effect on mRNA expression by		Association to epilepsy / seizures
	Physical Exercise	Bicuculline / Dalfampridine	
ACTA2	-1.58	Decreased [1]	The R179H mutation in ACTA2 results in neonatal stroke and progressive leukoencephalopathy[21].
ARC	1.68	Increased	Immediate early gene, increased in epilepsy (6.2x)[22].
BTG2	1.57	Increased	-
DUSP1	1.38	Increased	Increased in epilepsy (3.4x)[22].
FOS	2.26	Increased	Immediate early gene, increased in epilepsy (4.2x)[22].
FOSB	1.24	Increased	Immediate early gene, increased in epilepsy (3.0x)[22].
GADD45G	1.23	Increased	Increased in epilepsy (2.0x)[22].
NPAS4	1.85	Increased	Npas4 inhibits seizures in pilocarpine-induced epileptic rats[23].
NR4A1	1.24	Increased	Immediate early gene[24], increased in epilepsy (4.7x)[22].

[1] Only regulated by Bicuculline

**Supplementary Table 14.** Differentially expressed mRNAs in the PPN due to physical exercise in MPTP-treated mice, *and* regulated by CREB1. For each gene/mRNA the cellular process in which they exert an effect is described in the column 'Involved in'.			
Gene	Effect on mRNA expression by		Involved in[20]:
	Physical Exercise	CREB1	
CALCB	-2.10	Increased	Vascular remodeling
CALN1	1.23	Decreased	Calcium signaling
CARTPT	1.47	Increased	Neuropeptide signaling
EBP	-1.24	Increased	Cholesterol/lipid signaling
FAM65B	-1.27	Increased	Cytoskeleton rearrangement
FASN	1.21	Increased	Cholesterol/lipid signaling
JUN	1.22	Regulated	Immediate early gene; Increased in epilepsy (1.6x)[22].
LMO1	-1.49	Decreased	Transcription regulator
MVK	1.27	Increased	Cholesterol/lipid signaling
NAB2	-1.27	Increased	Transcription regulator
NOS1	1.52	Regulated	Nitric oxide signaling
NR4A1	1.27	Increased	Immediate early gene[24]; Increased in epilepsy (4.7x)[22].
NRGN	-1.23	Decreased	Calcium signaling
NRP1	-1.22	Decreased	Vascular remodeling; Calcium signaling
PENK	-1.26	Increased	Neuropeptide signaling
SGK1	-1.54	Increased	Vascular remodeling
SV2C	1.35	Increased	Increased in epilepsy (2.1x)[22].
TAC1	-1.36	Increased	Neuropeptide signaling; Vascular remodeling; Increased in epilepsy (1.8x)[22].
TINF2	1.32	Increased	Telomere regulation

DESCRIPTION OF THE MOLECULAR LANDSCAPES

TABLE OF CONTENTS

INTRODUCTION 22

1. Molecular landscape of the SN **23**

1.1 G-coupled receptor signaling and cytoskeleton regulation 23

1.2 The regulation of cell survival 23

1.3 Circadian clock proteins 24

1.4 Regulation by physical exercise vs. L-DOPA 25

2. Molecular landscape of the VM **25**

2.1 DA- and interneuron-mediated MSN activation 25

2.2 Main signaling cascades 27

2.3 Endogenous cannabinoid signaling 28

2.4 Neuropeptides 29

2.5 Regulation by physical exercise vs L-DOPA 29

3. Molecular landscape of the DL **30**

3.1 Regulation by physical exercise vs. RICTOR 30

4. Molecular landscape of the VTA **31**

4.1 Regulation by physical exercise vs. RICTOR 31

5. Molecular landscape of the PFC **31**

5.1 Regulation by physical exercise vs. Bicuculline/Dalfampridine 32

6. Molecular landscape of the PPN **32**

6.1 Regulation by physical exercise vs. CREB1 33

REFERENCES **34**

INTRODUCTION

Ingenuity analysis of the mRNA sequencing (RNAseq) data of the MPTP-treated mice *with* physical exercise compared to MPTP-treated mice *without* physical exercise revealed the top regulator in each of the PD-related brain areas. In the SN and the VM, L-DOPA is the top regulator, whereas RICTOR is the top regulator in the DL and VTA, Bicuculline/Dalfampridine are the top regulators in the PFC, and CREB1 is the top regulator in the PPN. For each brain area a molecular landscape was built that represents the interactions between the proteins encoded by the mRNAs regulated by the top regulator and physical exercise. **Figure 4** and **Figure 5** show the molecular landscapes of the SN and VM, respectively, and the landscapes for the DL, VTA, PFC and PPN are shown in **Supplementary Figure 6-9**. Below, a description of the interactions that are represented in the molecular landscape figures is given.

All names of proteins derived from the mRNAs that were differentially expressed in the RNAseq due to physical exercise are shown in **bold** and proteins that are associated with PD (via genetic evidence, or differentially expressed in PD patients compared to controls) are (also) single-underlined. For a complete overview of all proteins in the landscapes (and the corroborating evidence for their associations with PD), see **Supplementary Tables 9-14**. In the molecular landscape descriptions below, the gene name abbreviations refer to both the gene and the protein. Furthermore, the terms 'activates' and 'inhibits' indicate effects on protein function (e.g. by (de)phosphorylation) by another protein, while 'increases the expression of' or 'decreases the expression of' denote effects on the abundance of a protein (directly or indirectly) induced by another protein.

Not all proteins encoded by mRNAs from the RNAseq were placed in the landscape, either because there were no connections with other landscape proteins or due to lack of annotation, or both. However, this does not necessarily mean that they are not involved in physical exercise-mediated effects. Interactions in the landscape that are (for practical reasons) not shown in the figures are indicated with '(not shown)' in the text.

Overall, in order to interpret protein-protein interactions in the landscape, two generalizations have been made. First, when a knock-out of protein A in a cell or animal model *increases* the expression of protein B, we assume that endogenous expression of protein A leads to the opposite effect and *decreases* the expression of protein B. Second, we assumed that all identified protein interactions (in any organism and/or cell type) can be extrapolated to the interactions in human (DA) neurons, even when the specific interactions have not been studied in these specific substrates.

1. Molecular landscape of the SN

In **Figure 4** the physical exercise-mediated changes in expression in the SN are shown in combination with the L-DOPA-mediated expression of the same proteins. In **Supplementary Table 9** an overview of the regulatory effects of physical exercise and L-DOPA in the SN is given. First, in the description below, the interactions and pathways of these proteins in the landscape are discussed (independent of their regulation by physical exercise or L-DOPA). And, secondly, the last paragraph of this section discusses the differential effects of physical exercise and L-DOPA on these pathways.

The central themes in the landscapes that represent the protein interactions associated with the changes in expression due to physical exercise in the SN are G-coupled receptor signaling, the regulation of cell survival (including ERK1/2 signaling, ROS regulation, glucose uptake and signaling and CREB1 signaling), as well as circadian clock proteins. Here we describe concisely the proteins involved in each theme, and their interactions.

1.1 G-coupled receptor signaling and cytoskeleton regulation

ARRB2 mediates the signaling by G-protein coupled receptors and is e.g. activated by the G-coupled receptor **GRP39**[25]. **ARRB2** binds phosphorylated AKT1 (total and Ser473-phosphorylated AKT1 are lower in the PD brain, but are increased in glia cells in the SN of PD patients[26]) and facilitates the inactivation of AKT1[27], and binds to the cytoskeleton protein **ACTA2**[28], to the familial PD[29-33] proteins VPS35[28] and PARK2[34], to the nuclear export adapter **ALYREF**[28] (binds also to AKT1[35]), and to the small GTPase CDC42[36] that is known to control actin polymerization and thereby e.g. affects cell morphology and endocytosis. **ACTA2** also binds the familial PD[37-41] proteins LRRK2[42] and PARK2[43], and binds **TP53**[44]. **TP53**, in turn, increases **ACTA2** expression[45, 46]. LRRK2 and **CDC42EP3** bind[47], while **CDC42EP3** and **CDC42EP2** both bind CDC42[48, 49], thereby regulating assembly of actin filaments[20]. Furthermore, both **CDC42EP2** and **CDC42EP3** both bind to the familial PD[50-54] protein SNCA[55].

1.2 The regulation of cell survival

The ERK1/2 pathway is activated by a wide array of stimuli, including growth factors, cytokines and ligands for G protein-coupled receptors, and regulates neuron proliferation, survival and apoptosis.

In the molecular landscape represented here, ERK1/2 is activated by **PPP1R1B**[56], **ACKR1**[57], **EDNRB**[58] (also binds to PARK2[59]), **IER3**[60] and **SHC1**[61, 62]. ERK1/2 also binds to and is regulated by **TP53**[63-65], and binds to and is inhibited by **DUSP6**[66-69]. Moreover, ERK1/2 expression is increased by **GPR39**[70]. These proteins are also known to affect pathways involved in cell death, i.e. **IER3**, **TP53** and **SHC1** activate CASP3[71-74] and thereby induce apoptosis. CASP3 expression is increased in the SN of PD patients[75, 76]. Moreover, **IER3** inhibits the activation and expression of AKT1[77], and **TP53** binds to **ALYREF**[78] and binds to[79, 80] and decreases the expression of PARK7[81], whereas PARK7 activates **TP53**[81]. Further,

TP53 increases the expression of **DUSP6**[82], **FKBP5**[83, 84], **PDGFA**[85] and **IER3**[86-88]. **SHC1** is also important for the cellular response to oxidative stress[20] and functions downstream of **TP53** to induce apoptosis[20]. While reactive oxygen species (ROS) are necessary for normal cell functioning[89, 90], high levels are toxic and induce cell death[89, 91, 92]. The mitochondrial protein **ROMO1** increases the production of ROS[93]. The ferritin light chain protein **FTL**, on the other hand, reduces the formation of ROS by regulating iron homeostasis[94] and also the heat shock protein **HSPB6** decreases the production of ROS[95]. **HSPB6** binds SNCA[96], increases calcium levels[97], activates AKT1[95], and inhibits CASP3 signaling[95]. Cytoplasmic calcium levels are decreased by **PVALB**[98], that is increased in a subgroup of PD DA SN neurons[9].

ERK1/2 activation, in turn, increases the expression of the glucose transporter **SLC2A1**[99]. Glucose influx is necessary for maintenance of cellular energy levels and cell survival, and PD patients show increased metabolic rates of glucose in the SN[100]. The glucose transporter **SLC2A1** regulates the influx of glucose into the cell and inhibits the activation of CASP3[101]. **SLC2A1** expression is also increased by AKT1[102-104] and the familial PD[105-107] protein PINK1[108], but decreased by **TP53**[109-111]. Further, the growth factor complex PDGF (composed of **PDGFA** and PDGFB) activates **SLC2A1**[112] thus increases the uptake of glucose[113]. The neuropeptide **TAC1** inhibits glucose uptake[114, 115] and thereby also increases extracellular (blood) glucose levels[115, 116]. Moreover, glucose activates ERK1/2[117, 118] and CDC42[119, 120], increases the expression of the anti-apoptotic proteins PARK2[121], PINK1[121] and **SHC1**[122] and decreases the expression of **TP53**[123, 124] and **ACTA2**[125]. Thus, the maintenance of sufficiently high cellular glucose levels prevents ROS formation and activation of apoptotic signaling cascades.

Lastly, ERK1/2 also decreases the expression of **FKBP5**[126] (part of the steroid receptor complex[20] and binds AKT1[127]) and activates **SHC1**[128], **IER3**[129] and CREB1[130-132]. CREB1 is a transcription factor that binds to cAMP response elements on the DNA and thereby regulates gene transcription of e.g. TH (not shown)[133, 134]. The earlier mentioned **PPP1R1B** (**DARPP-32**) integrates the signals in response to extracellular DA and glutamate[135, 136] and activates CREB1 (in response to DRD2 activation)[137, 138], increases the expression of LRRK2[139], SNCA[139] and CREB1[140] and increases mobilization of calcium[141]. CREB1 is also activated by glucose[142], calcium[143] and AKT1[144-147], binds **TP53**[148-150] and binds to the nuclear exporter adapter **ALYREF**[151] that transports spliced mRNAs from the nucleus to the cytoplasm. CREB1 increases the expression of **MFSD2A**[152] and **IER3**[152] and regulates the expression of the circadian clock proteins **PER1** and **CIART** (see below).

1.3 Circadian clock proteins

Multiple studies have reported a dysregulation of the circadian clock in PD patients and e.g. sleep-wake disturbances and increased daytime sleepiness are widely studied[153-159]. Also degeneration of DA neurons in the SN of MPTP-treated mice cause circadian rhythm irregularities[160]. In the SN

landscape, the proteins **CIART** (not shown), **DBP** and **PER1** are involved in circadian clock regulation. CREB1 increases the expression of **PER1**[152, 161, 162] and is necessary for expression of **CIART**[162]. Calcium increases the expression of both **PER1**[163] and **DBP**[163], and **TP53** (a regulator of the circadian clock[164]) increases the expression of **DBP**[46], whereas **DBP** and **PER1** increases each other's expression[165]. Thus, multiple proteins involved in regulation of the circadian rhythm are regulated in the SN landscape, suggesting that physical exercise might affect the (dysregulated) circadian clock function in PD.

1.4 Regulation by physical exercise vs. L-DOPA

Physical exercise and L-DOPA have an opposite effect on the regulation of proteins involved in glucose homeostasis (**SLC2A1**), ERK1/2 activation (**ACKR1**, **EDNRB**, **GPR39**, **IER3**, **TP53**), the DARPP-32 pathway (**PPP1R1B**) and circadian clock-regulating proteins (**PER1**, **DBP**, **CIART**). Namely, physical exercise downregulates the expression of **SLC2A1**, **PPP1R1B** and clock proteins, while (chronic) L-DOPA administration results in increased **SLC2A1** expression and (over)activation of the neuron by L-DOPA-mediated activation of ERK1/2[56, 166], **PPP1R1B**[167] and CREB1[168] and the subsequent *activation* of clock proteins. Further, in addition to the proteins differentially expressed due to physical exercise, L-DOPA activates[166] and increases the expression of CASP3[166, 169].

2. Molecular landscape of the VM

In **Figure 5** the physical exercise-mediated changes in expression in the VM are shown, in combination with the L-DOPA-mediated expression of these same proteins. **Supplementary Table 10** gives an overview of the regulatory effects of physical exercise and L-DOPA. The central themes in the landscapes that represent the protein interactions associated with the changes in expression due to physical exercise in the VM are (interneuron mediated) DA release, cannabinoid signaling, neuropeptide signaling, calcium mobilization and subsequent activation of **PPP1R1B**, ERK1/2, CREB1 and **CCND1** signaling. Here we describe concisely the proteins involved in each theme, and their interactions. First, in the description below, the interactions and pathways of these proteins in the landscape are discussed (independent of their regulation by physical exercise or L-DOPA). Secondly, the last paragraph of this section discusses the differential effects of physical exercise and L-DOPA on these pathways.

2.1 DA- and interneuron-mediated MSN activation

Medium spiny neurons (MSNs) are GABAergic inhibitory cells and represent the majority of cells present in the striatum. There are two primary MSNs subtypes, the DRD1 and DRD2 expressing MSNs, representing the direct and indirect pathway respectively. MSNs of the direct pathway project

to the globus pallidus internal segment (GPi) and substantia nigra pars reticularis (SNr), whereas the indirect pathway MSNs project to the external segment of the globus pallidus (GPe), to the subthalamic nucleus and subsequently to the GPi and SNr. Outputs of the direct and indirect pathway, respectively, cause an excitation and inhibition of the upper motor neurons in the cortex[170, 171].

DA release in the striatum is not only dependent on SN neuron activation, but also depends on the activity of cholinergic interneurons. Cholinergic interneurons integrate synaptic signaling in the striatum and mediate DA-dependent striatal plasticity of MSNs[172-175]. Through the activation of both muscarinic and nicotinic cholinergic receptors on DA terminals and the co-release of glutamate, activated cholinergic interneurons trigger striatal DA release[176-183]. Of note, in PD and in dystonia, a reduced release of DA in the striatum leads to an increased acetylcholine (ACh) release by interneurons[184], whereas DA-dependent pauses in the tonic firing of cholinergic interneurons are hypothesized to function as a learning mechanism in reward- and motor-related learning[185-187]. Further, the differentially expressed potassium channels (**KCNA5**, <u>**KCNA6**</u>, **KCNC4**, **KCNE5**) may regulate DA release and presynaptic <u>DRD2</u> function[188], or may regulate the firing patterns of cholinergic interneurons via hyperpolarization-activated potassium currents[189, 190] or may be involved in depolarization of MSNs after activation of muscarinic ACh receptors[191-194].

Thus, the interplay between striatal ACh and DA release, by respectively cholinergic interneurons and DA neurons, is important for learning and plasticity of MSNs. Another factor involved in memory and learning is the transcription factor <u>**MEF2C**</u>, that suppresses the number of excitatory synapses on neurons[20]. <u>**MEF2C**</u> increases the expression of **KCNA5**[195], and <u>**MEF2C**</u> itself is activated by **MAPK11**[196, 197] and <u>**MEF2C**</u> expression is increased by **HLA-A**[198] and <u>BDNF</u>[199, 200] (not shown; BDNF is associated with (cognitive impairment in) PD[201-203]).

In the VM landscape, the choline O-acetyltransferase **CHAT**, necessary for ACh synthesis in cholinergic synapses, is decreased by physical exercise. Moreover, activation of the neuronal acetylcholine receptor subunit <u>**CHRNA4**</u> by ACh leads to opening of an ion channel, influx of calcium and depolarization that facilitates activation of the **SNARE** complex and neurotransmitter (e.g. DA) release[204, 205]. Depolarization through calcium influx is also mediated by the voltage-dependent calcium channel (<u>**VDCC**</u>)[20], that binds to **RIMS1**[206, 207] and **UNC13C**[208] and is inhibited by **REM2**[209]. The **SNARE** complex binds <u>**STXBP1**</u>[210, 211] and **RIMS1**[206] and **SNARE** assembly is inhibited by calmodulin (CaM)[212]. <u>**STXBP1**</u> regulates synaptic vesicle docking and fusion and binds also to CaM[213], **DOC2B**[214] (**DOC2B** expression is increased by CaM[215]), **APBA2**[216, 217] and familial PD protein <u>SNCA</u>[218].

Cytoplasmic vesicles are recycled and are again filled with DA for release into the synaptic cleft. Removal of <u>**SYN1**</u> from the cytoplasmic side of cytoplasmic vesicles mobilizes the vesicles for

neurotransmitter release[20]. **SYN1** binds **SH3GL2**[219], LRRK2[220], PARK2[221], SNCA[222] and CaM[213, 223]. SNCA decreases the expression of **SYN1**[224], whereas CaM binds the synaptic vesicle membrane protein **VAT1L**[213] and induces the release of **SYN1** from cytoplasmic vesicles[20]. **SH3GL2** regulates synaptic vesicle endocytosis and binds in addition to **SYN1** also to DRD2[225], PARK2[226], LRRK2[227] and CaM[213]. DA for release into the synaptic cleft is either synthesized in the DA neuron, or is taken up by the DA transporter SLC6A3 (that is associated with PD[228-230]) from the extracellular matrix[231]. TH is the rate-limiting enzyme in DA synthesis and binds SNCA[232]. TH is activated by calcium[233, 234] and inhibited by SNCA[232, 235] and DRD2[236]. Further, the TH expression is increased by the neuropeptide **TAC1**[237] and the SNCA-homologue **SNCB**[238]. **SNCB** also binds to SNCA[238], AKT1[239] and inhibits CASP3 activation[240]. Cytoplasmic DA is transported into the cytoplasmic vesicles by the DA transporter SLC18A2[241] (that is associated with PD[242, 243]) so it can be released. The protein **MYO1B** is a motor protein involved in among others vesicular transport and binds to LRRK2[42]. **CDC42EP3** is another protein involved in cytoskeleton regulation and binds to SNCA[55] and LRRK2[47].

In summary, DA release in the striatum is regulated by controlling reuptake, storage and synthesis of DA and thereby controlling the availability of DA ready for release. However, it should be noted that the proteins involved in vesicle fusion and release of neurotransmitter are not only involved in DA release in the synaptic cleft, but may also mediate e.g. ACh, GABA or glutamate release.

DA released in the synaptic cleft activates the DRD1 and DRD2 receptors present on MSNs[244]. The PD-associated DRD2[245, 246] binds to DRD1[247] and also to the chloride ion channel **CLIC6**[248]. In turn, DA receptor activation results in increased cytosolic calcium concentration[249, 250]. Cytoplasmic calcium levels are also increased by **TAC1**[251-253], **VDCC**[254] (binds to **AMIGO2**[255]), **TRPC3**[256-258] and decreased by **SLC24A2**[20, 259], **HTR1B**[260] and **PVALB**[98, 261, 262]. Calcium increases the expression of **ARC**[263] and **CCND1**[264, 265] and activates **FAAH**[266], **PPP1R1B**[267], ERK1/2[268, 269] and CaM[270, 271]. CaM is a calcium sensor and transduces the calcium signal and thereby increases the expression of **LRRTM3**[215], binds and decreases the expression of **TCF4**[272, 273] (**TCF4** also binds to **TLE1**[274] and PARK2[275]), binds **TRPC3**[276], **CCND1**[277], **STRN**[278], **MYO1B**[279, 280], **MAPK11**[281] (binds also to **MBP**[282]), **ARPP21**[283], **MBP**[284, 285] (**MBP** expression is increased by BDNF (not shown)[286]) and **RAP1GAP**[213]. Moreover, CaM activates **CAMK1G**[287] that in turn activates CREB1[20, 287]. Calcium mobilization therefore increases the main signaling cascades in the VM landscape; **PPP1R1B**, ERK1/2, CREB1 and **CCND1** signaling.

2.2 Main signaling cascades

Increased phosphorylation (and thus activation) of **PPP1R1B** and ERK1/2 in striatal neurons after chronic L-DOPA treatment is associated with L-DOPA-induced dyskinesias[56, 288, 289]. **PPP1R1B** is also known as dopamine- and cAMP-regulated neuronal phosphoprotein (DARPP-32) and activates ERK1/2[56, 290] and CREB1[137, 140], inhibits CASP3[140, 291] and regulates AKT1 activation[138, 292].

ERK1/2 are protein kinases that are signal transducers for e.g. growth factors, cytokines and G protein-coupled receptors. In addition to activation by **PPP1R1B**, ERK1/2 are activated by **HBEGF**[293-295] (regulates the survival of midbrain dopaminergic neurons[296]), **DLK1** [297, 298], **GNAS**[299-301], **DGKI**[302], **HTR1B**[303] and the endogenous cannabinoid receptor **CNR1**[304-307] (see also below), and are inhibited by **DUSP6**[67, 69, 308, 309] and **RAP1GAP**[310]. ERK1/2 increase the expression of neuropeptide **TAC1**[311] and activate CREB1[131, 132].

The transcription factor CREB1 is, in addition to **CAMK1G**, **PPP1R1B** and ERK1/2 (see above), also activated by glucose[142, 312] (that decreases the expression of **CBR3**[313]), potassium[314], AKT1[144-146, 315] and **GNAS**[316]. Subsequently, CREB1 regulates the expression of **GABRG2**[22], decreases the expression of **DLK1**[317] (also decreased by **KLF6**[318]), **PGM2L1**[152], **PTPRD**[152], **MYO1B**[152] and **CRYM**[152] and increases the expression of **ARC**[162], **NPTX2**[152], **ECEL1**[152], BDNF (not shown)[143, 152, 319], **KCNC4**[152], **HLA-A**[152] (not shown, **HLA-A** binds **MBP**[320] and **GNAS**[321], and its expression is decreased by ERK1/2 (not shown)[322]) and of the neuropeptides **TAC1**[22, 152], **PENK**[152, 323] and **PDYN**[324, 325].

All three, **PPP1R1B**, ERK1/2 and CREB1, increase the expression of the cell cycle regulator **CCND1** [152, 292, 326-328]. **CCND1** may be involved in synaptic plasticity and learning[329] and its expression is increased by BDNF[330], **WNT7B**[331], AKT1 [332-334], glucose[335], calcium[264, 265] and **TCF4**[336, 337] and decreased by **RAP1GAP**[310], PARK2[338], **ZEB2**[339] (**ZEB2** inhibits AKT1)[340] and **CTNNBIP1**[336]. Nuclear translocation of **CCND1** is activated by AKT1[341] and calcium[264] and inhibited by **KLF6**[342]. **CCND1** decreases the expression of **STXBP1**[343] and increases the expression of **CTNNBIP1**[344]. Further, **CCND1** binds **KLF6**[342], **LMNA**[345] (AKT1 activates **LMNA** [346]) and **MAPK11**[347].

Thus, **CCND1** is regulated by all the main signaling cascades in the landscape (**PPP1R1B**, ERK1/2, CREB1) and may affect synaptic plasticity.

2.3 Endogenous cannabinoid signaling

Following DA depletion, the endocannabinoid system in the basal ganglia rearranges to restore homeostasis[348, 349]. The cannabinoid receptor **CNR1** is abundant within the basal ganglia[350], can repress the release of glutamate and GABA (e.g. by cortical synapses, not shown)[351, 352] and interacts with DA transmission in the striatum[353, 354], i.e. **CNR1** forms heterodimers with DA receptors[354] and **CNR1** activation increases DRD2 expression[355]. Further, indirect pathway signaling (via DRD2-positive MSNs) is rescued by endocannabinoid signaling and improves motor dysfunction in PD models[356, 357]. Paradoxically, both **CNR1** agonist and antagonists alleviate L-DOPA-induced dyskinesia in PD models[358]. This contradiction may be explained by the differential coupling of **CNR1** to G proteins, either due to interaction with DA receptors[353], or by the functional selectivity of the different agonists and antagonists for G proteins and **CNR1** subpopulations[358]. Moreover, DRD2 activation modulates coupling of different G proteins to **CNR1**[359] (not shown). **CNR1** expression is increased by

DA[360], glucose[361] and BDNF[362] (and **CNR1** increases BDNF expression[355, 363]). **CNR1** binds and decreases the expression of **RAP1GAP**[364] and binds and inhibits **VDCC**[208, 365, 366]. Further, **CNR1** decreases the expression of the neuropeptides **PDYN**[367] and **PENK**[367], regulates the activation of ERK1/2[304-306, 368, 369] and increases DA release[370, 371].

The two most abundant endocannabinoids, 2-arachidonoylglycerol (2-AG) and anandamide, bind and activate **CNR1**[372-374] and 2-AG also increases the expression of **CNR1**[375]. 2-AG and anandamide inhibit **FAAH** (not shown)[376], whereas **FAAH** in turn increases the hydrolysis and breakdown of both endocannabinoids[366, 377]. **GABRG2** and **GABRA3** form a GABA receptor complex[378, 379] and are activated by 2-AG[379]. Further, DA decreases the expression of **GABRG2**[360].

In summary, the endocannabinoid system interacts with and compensates for defects in the DA system and may therefore have a therapeutic potential in the treatment of PD.

2.4 Neuropeptides

PD and L-DOPA-induced dyskinesia are associated with abnormal expression of striatal precursor peptides, i.e. **PENK**, **PDYN** and **TAC1** derived peptides are increased in the globus pallidus and their production is DA-state dependent[380-382]. Neuropeptides are small peptides used for communication between neurons. As shown above, CREB1 increases the expression of the neuropeptides **PENK**, **PDYN** and **TAC1**. **PENK** decreases the excretion of K^+ (potassium)[383], whereas K^+ increases the expression of **PENK**[384]. **PENK** expression is increased by DA[385, 386] and BDNF[387] and decreased by DRD2[388]. **PDYN** decreases degeneration of DA neurons in the rat midbrain[389]. **PDYN** expression is increased by glucose[390], DRD1[391] and **TAC1**[392]. **TAC1** expression is increased by BDNF[393, 394] (not shown), DA[360], DRD2[388] and ERK1/2[311]. In turn, **TAC1** activates ERK1/2[395, 396] (not shown), increases plasma glucose levels (by decreasing its uptake in cells)[114-116], increases intracellular calcium[251-253], binds CaM[397] (not shown) and increases the expression of TH[237] and **GFAP**[398] (not shown; **GFAP** is a marker for astrocytes and its expression is increased by BDNF[399] (not shown) and decreased by glucose[313] (not shown)). The mitochondrial **GPD2** binds PARK2[43], and when activated, increases the release of ROS from the mitochondria[400]. Calcium binds and activates **GPD2**[20], whereas glucose inhibits **GPD2** activation[401] and may therefore regulate ROS levels in the cell.

In summary, the regulation of neuropeptides is DA-state dependent and may affect neuron communication and functioning in the striatum and globus pallidus.

2.5 Regulation by physical exercise vs L-DOPA

The main signaling themes of the landscape encompass ACh interneuron functioning, potassium signaling, vesicle release, cannabinoid and neuropeptide signaling, calcium mobilization and subsequent activation of **PPP1R1B**, ERK1/2, CREB1 and **CCND1** signaling. Strikingly, physical exercise

and L-DOPA have opposite effects on the regulation of almost all proteins in this landscape, i.e. physical exercise increases the expression of vesicle release proteins (**STXBP1**, **RIMS1**, **SNARE**, **APBA2**), **CCND1** and proteins involved in cannabinoid signaling (**FAAH**, **CNR1**) and decreases the expression of **PPP1R1B**, neuropeptides (**PDYN**, **PENK**, **TAC1**) or proteins involved in ERK1/2 regulation (**HTR1B**, **DGKI**, **HBEGF**, **DLK1**, **RAP1GAP**). L-DOPA exerts an opposite effect on the expression of all these proteins compared to the effect of physical exercise and also activates **PPP1R1B**[167], CREB1[168], ERK1/2[56, 166], DRD1 and DRD2[402], and activates and increases the expression of CASP3 [166, 169]. Of note, L-DOPA disrupts the crosstalk of an **CNR1**-DRD2 complex[403] and an **CNR1** agonist reduces L-DOPA-induced motor dysfunction in a PD rat model[404, 405]. Further, L-DOPA-induced dyskinesia is associated with increased neuropeptide levels in the striatum and globus pallidus[381, 382, 406-408]. Furthermore, ablation of striatal cholinergic interneurons attenuates L-DOPA-induced dyskinesia in mice[409, 410]. Of interest, whereas physical exercise decreases the ACh synthase **CHAT** and increase the expression of the ACh receptor subunit **CHRNA4**, L-DOPA causes the exact opposite.

In summary, physical exercise has been shown to have an opposite effect on the regulation of proteins that are regulated by L-DOPA and associated with L-DOPA-induced dyskinesia. Physical exercise may therefore have therapeutic value in attenuating the side effects of chronic L-DOPA use.

3. Molecular landscape of the DL

In **Supplementary Figure 6** the physical exercise-mediated changes in expression in the DL are shown in combination with the RICTOR-mediated expression of the same proteins.

The proteins regulated by both physical exercise and RICTOR are part of complex I-V of the electron transport chain in the mitochondria, the 40S and 60S ribosomal subunits, or the proteasome. **Supplementary Table 11** shows the localization of each of the proteins in these complexes and also provides an overview of the regulatory effects of physical exercise and RICTOR.

3.1 Regulation by physical exercise vs. RICTOR

RICTOR decreases the expression of all proteins in the landscape, and thereby regulates cellular energy levels, protein translation and degradation. In contrast to RICTOR, physical exercise increases the expression of all proteins in the landscape located in the electron transport chain, and also increases the expression of the proteins that are part of the ribosome or proteasome.

4. Molecular landscape of the VTA

In **Supplementary Figure 7** the physical exercise-mediated changes in expression in the VTA are shown in combination with the RICTOR-mediated expression of the same proteins.

The proteins regulated by both physical exercise and RICTOR are part of complex I-V of the electron transport chain in the mitochondria, the 40S and 60S ribosomal subunits, or the proteasome. **Supplementary Table 12** shows the localization of each of the proteins in these complexes and also provides an overview of the regulatory effects of physical exercise and RICTOR.

4.1 Regulation by physical exercise vs. RICTOR

Physical exercise and RICTOR both decrease the expression of all mRNAs in the landscape of the VTA. Therefore, physical exercise and RICTOR exert the same direction of effect on the functioning of the electron transport chain, the ribosome and the proteasome. This is in contrast to the opposite regulation of mRNAs in the ribosome, proteasome and especially the electron transport chain by physical exercise and RICTOR in the DL (see above).

5. Molecular landscape of the PFC

In **Supplementary Figure 8** the physical exercise-mediated changes in expression in the PFC are shown in combination with the Bicuculline/Dalfampridine-mediated expression of the same proteins. The drugs Bicuculline and Dalfampridine induce epileptic seizures, and eight out of nine of the mRNAs regulated by physical exercise and these convulsants are associated with epilepsy or seizures (**Supplementary Table 13**).

The main theme in the PFC landscape is immediate early gene activation, namely three out of nine proteins in the landscape – **FOS**, **FOSB** and **NR4A1** – are encoded by immediate early genes. **FOS** and **FOSB** decrease each other's expression[411] and **NR4A1** inhibits activation of **FOSB**[412]. The activation of these immediate early genes is regulated by insulin and low density lipoprotein (LDL). Impaired insulin homeostasis is associated with PD[413] and oxidized LDL is increased in the plasma of (L-DOPA-treated) PD patients[414]. Oxidized LDL regulates the expression of **FOS**[415, 416], and increases the expression of **FOSB**[417] and **NR4A1**[417, 418] and both insulin and LDL increase the expression of **DUSP1**[419-421] and activate the PI3K complex[337, 422] in the cytoplasm. The PI3K complex activates **NR4A1**[423] and ERK1/2[424-426] and increases the expression of **FOS**[411, 427, 428]. Oxidized LDL activates the ERK1/2 pathway[429] that is also activated by insulin (via PI3K activation)[422, 425, 430-432]. Further, ERK1/2 is also regulated by the familial PD proteins, i.e. both SNCA and PARK2 inhibit ERK1/2 activation[433, 434] and SNCA also binds to ERK1/2[433]. Furthermore, ERK1/2 is activated by the NMDA receptor (NMDAR)[435,

[436] that also increases the expression of **FOS**[411] and **ARC**[437] and is involved in **DUSP1** regulation[438] (not shown). The phosphatase **DUSP1** binds to ERK1/2[439] and inhibits ERK1/2 activation[440, 441]. In turn ERK1/2 activates **DUSP1**[442, 443] and **FOS** [444, 445], regulates **BTG2**[446], and also increases the expression of **FOS**[447, 448], **FOSB**[449] and regulates the expression of **DUSP1**[450]. Insulin expression/secretion is regulated by **PARK2**[451], increased by **BTG2**[452] and decreased by **NPAS4**[453] and **NR4A1**[454]. The actin protein **ACTA2** binds to the familial PD proteins LRRK2[42] and PARK2[43]. Insulin increases the level of intracellular glucose, which is mediated by PI3K[113, 455, 456]. In turn, glucose decreases the expression of **ACTA2**[125], increases the expression of PARK2[121] and **NPAS4**[453], activates PI3K[457, 458] and activates ERK1/2[117, 118].

Thus, immediate early gene activation and the epilepsy-related signaling are the main pathways in the PFC regulated by both physical exercise and Bicuculline/Dalfampridine. Further, (oxidized) LDL, insulin, glucose and the ERK1/2 and PI3K pathways seem important for the regulation of these proteins.

5.1 Regulation by physical exercise vs. Bicuculline/Dalfampridine

Physical exercise and Bicuculline/Dalfampridine exert the same direction of effect on all mRNAs in the PFC landscape, i.e. they decrease the expression of ACTA2 and increase the expression of the other eight proteins. Bicuculline/Dalfampridine treatment and physical exercise may therefore (partially) have the same effects on the molecular pathways in the PFC. The mRNAs coding for NMDAR and ERK1/2 did not show a differential expression due to physical exercise, but are both regulated by Bicuculline[459, 460].

6. Molecular landscape of the PPN

In **Supplementary Figure 9** the physical exercise-mediated changes in expression in the PPN are shown in combination with the CREB1-mediated expression of the same proteins. These proteins show very limited interactions in the landscape, but a few themes could be distinguished such as vascular remodeling / angiogenesis (**CALCB**, **NRP1**, **SGK1**, **TAC1**), neuropeptide signaling (**CARTPT**, **PENK**, **TAC1**), lipid metabolism (**EBP**, **FASN**, **MVK**), epilepsy/immediate early response (**JUN**, **NR4A1**, **SV2C**, **TAC1**) and calcium singaling (**CALN1**, **NRGN**, **NRP1**, Calmodulin (CaM)) (see also **Supplementary Table 14**).

Central in the landscape is the transcription factor CREB1 that regulates the expression of **JUN**[461, 462] and **NOS1**[463], increases the expression of **NR4A1**[152, 464], **FASN**[465], **MVK**[162], **EBP**[152], **TINF2**[152], **FAM65B**[152], **SGK1**[152], **CARTPT**[152], **CALCB**[466], **SVC2C**[152], **NAB2**[162], **PENK**[152] and **TAC1**[152] and decreases the expression

of **CALN1**[152] (binds calcium[20]), **LMO1**[152], **NRGN**[152] and **NRP1**[152]. Further, CREB1 binds to **JUN**[467], **SGK1**[468] and is activated by **SGK1**[468], **NOS1**[469] and CaM[411]. The proteins of these CREB1-regulated mRNAs show only a small number of interactions with each other. The immediate early response proteins **JUN** and **NR4A1** bind[470] and **JUN** inhibits **NR4A1** activation[470]. Further, **JUN** regulates the expression of **LMO1**[471], increases the expression of **PENK**[472] and **SGK1**[473] and mediates alternative splicing of PARK2[474]. **NR4A1** regulates the expression of **FASN**[475] and is activated by **TAC1**[476]. Further, **CALCB** increases the expression of **CARTPT**[477]. Furthermore, **NOS1** binds to the calcium binding CaM[478, 479] that is binding to the calcium binding **NRGN**[480, 481]. **NOS1** is activated by CaM[479, 482], **NRP1**[483] (binds calcium[20]) and SNCA[484], and **NOS1** expression is decreased by PARK2[485]. Lastly, insulin increases the expression of **JUN**[486], **NR4A1**[475] and **FASN**[487] and activates **SGK1**[488] and **CREB1**[489] and may therefore be a modulating factor of the landscape.

6.1 Regulation by physical exercise vs. CREB1

Physical exercise increases the expression of mRNAs related to epilepsy/immediate early response, and decreases the mRNAs involved in neuropeptide signaling. However, whereas some of these proteins are increased by CREB1, others are decreased by CREB1 and no clear directional effect of CREB1 functioning due to physical exercise could be discerned.

REFERENCES

1. Paxinos G, Franklin KBJ. The Mouse Brain in Stereotaxic Coordinates. San Diego, CA: Academic Press; 2001.
2. Durrenberger PF, Grunblatt E, Fernando FS, Monoranu CM, Evans J, Riederer P, et al. Inflammatory Pathways in Parkinson's Disease; A BNE Microarray Study. Parkinson's disease. 2012;2012:214714.
3. Simunovic F, Yi M, Wang Y, Stephens R, Sonntag KC. Evidence for gender-specific transcriptional profiles of nigral dopamine neurons in Parkinson disease. PloS one. 2010;5(1):e8856.
4. Jin J, Hulette C, Wang Y, Zhang T, Pan C, Wadhwa R, et al. Proteomic identification of a stress protein, mortalin/mthsp70/GRP75: relevance to Parkinson disease. Molecular & cellular proteomics : MCP. 2006;5(7):1193-204.
5. Licker V, Cote M, Lobrinus JA, Rodrigo N, Kovari E, Hochstrasser DF, et al. Proteomic profiling of the substantia nigra demonstrates CNDP2 overexpression in Parkinson's disease. Journal of proteomics. 2012;75(15):4656-67.
6. Cantuti-Castelvetri I, Keller-McGandy C, Bouzou B, Asteris G, Clark TW, Frosch MP, et al. Effects of gender on nigral gene expression and parkinson disease. Neurobiology of disease. 2007;26(3):606-14.
7. Cash R, Raisman R, Ploska A, Agid Y. Dopamine D-1 receptor and cyclic AMP-dependent phosphorylation in Parkinson's disease. Journal of neurochemistry. 1987;49(4):1075-83.
8. Riley BE, Gardai SJ, Emig-Agius D, Bessarabova M, Ivliev AE, Schule B, et al. Systems-based analyses of brain regions functionally impacted in Parkinson's disease reveals underlying causal mechanisms. PloS one. 2014;9(8):e102909.
9. Soos J, Engelhardt JI, Siklos L, Havas L, Majtenyi K. The expression of PARP, NF-kappa B and parvalbumin is increased in Parkinson disease. Neuroreport. 2004;15(11):1715-8.
10. Nair VD, McNaught KS, Gonzalez-Maeso J, Sealfon SC, Olanow CW. p53 mediates nontranscriptional cell death in dopaminergic cells in response to proteasome inhibition. The Journal of biological chemistry. 2006;281(51):39550-60.
11. Zhang Y, James M, Middleton FA, Davis RL. Transcriptional analysis of multiple brain regions in Parkinson's disease supports the involvement of specific protein processing, energy metabolism, and signaling pathways, and suggests novel disease mechanisms. American journal of medical genetics Part B, Neuropsychiatric genetics : the official publication of the International Society of Psychiatric Genetics. 2005;137b(1):5-16.
12. Vogt IR, Lees AJ, Evert BO, Klockgether T, Bonin M, Wullner U. Transcriptional changes in multiple system atrophy and Parkinson's disease putamen. Experimental neurology. 2006;199(2):465-78.
13. Botta-Orfila T, Tolosa E, Gelpi E, Sanchez-Pla A, Marti MJ, Valldeoriola F, et al. Microarray expression analysis in idiopathic and LRRK2-associated Parkinson's disease. Neurobiology of disease. 2012;45(1):462-8.
14. Zhang LM, Chen B, Feng XL, Dong XM, Wang WZ. [Polymorphisms of neural nicotinic cholinergic receptor alpha 4 gene of Chinese]. Zhonghua yi xue yi chuan xue za zhi = Zhonghua yixue yichuanxue zazhi = Chinese journal of medical genetics. 2006;23(1):55-8.
15. Barrero FJ, Ampuero I, Morales B, Vives F, de Dios Luna Del Castillo J, Hoenicka J, et al. Depression in Parkinson's disease is related to a genetic polymorphism of the cannabinoid receptor gene (CNR1). The pharmacogenomics journal. 2005;5(2):135-41.
16. Greenbaum L, Tegeder I, Barhum Y, Melamed E, Roditi Y, Djaldetti R. Contribution of genetic variants to pain susceptibility in Parkinson disease. European journal of pain (London, England). 2012;16(9):1243-50.
17. Miller RM, Kiser GL, Kaysser-Kranich TM, Lockner RJ, Palaniappan C, Federoff HJ. Robust dysregulation of gene expression in substantia nigra and striatum in Parkinson's disease. Neurobiology of disease. 2006;21(2):305-13.
18. Nagatsu T, Sawada M. Biochemistry of postmortem brains in Parkinson's disease: historical overview and future prospects. Journal of neural transmission Supplementum. 2007(72):113-20.
19. Nakashima A, Ota A, Kaneko YS, Mori K, Nagasaki H, Nagatsu T. A possible pathophysiological role of tyrosine hydroxylase in Parkinson's disease suggested by postmortem brain biochemistry: a contribution for the special 70th birthday symposium in honor of Prof. Peter Riederer. Journal of neural transmission (Vienna, Austria : 1996). 2013;120(1):49-54.
20. UniProt. UniProt: a hub for protein information. Nucleic acids research. 2015;43(Database issue):D204-12.

21. Moosa AN, Traboulsi EI, Reid J, Prieto L, Moran R, Friedman NR. Neonatal stroke and progressive leukoencephalopathy in a child with an ACTA2 mutation. Journal of child neurology. 2013;28(4):531-4.
22. Beaumont TL, Yao B, Shah A, Kapatos G, Loeb JA. Layer-specific CREB target gene induction in human neocortical epilepsy. The Journal of neuroscience : the official journal of the Society for Neuroscience. 2012;32(41):14389-401.
23. Wang D, Ren M, Guo J, Yang G, Long X, Hu R, et al. The inhibitory effects of Npas4 on seizures in pilocarpine-induced epileptic rats. PloS one. 2014;9(12):e115801.
24. Maxwell MA, Muscat GE. The NR4A subgroup: immediate early response genes with pleiotropic physiological roles. Nuclear receptor signaling. 2006;4:e002.
25. Holst B, Egerod KL, Schild E, Vickers SP, Cheetham S, Gerlach LO, et al. GPR39 signaling is stimulated by zinc ions but not by obestatin. Endocrinology. 2007;148(1):13-20.
26. Timmons S, Coakley MF, Moloney AM, C ON. Akt signal transduction dysfunction in Parkinson's disease. Neuroscience letters. 2009;467(1):30-5.
27. Del'guidice T, Beaulieu JM. Messing up with traffic: different effects of antipsychotic agents on glutamate receptor complexes in vivo. Molecular pharmacology. 2008;73(5):1339-42.
28. Xiao K, McClatchy DB, Shukla AK, Zhao Y, Chen M, Shenoy SK, et al. Functional specialization of beta-arrestin interactions revealed by proteomic analysis. Proceedings of the National Academy of Sciences of the United States of America. 2007;104(29):12011-6.
29. Vilarino-Guell C, Wider C, Ross OA, Dachsel JC, Kachergus JM, Lincoln SJ, et al. VPS35 mutations in Parkinson disease. American journal of human genetics. 2011;89(1):162-7.
30. Zimprich A, Benet-Pages A, Struhal W, Graf E, Eck SH, Offman MN, et al. A mutation in VPS35, encoding a subunit of the retromer complex, causes late-onset Parkinson disease. American journal of human genetics. 2011;89(1):168-75.
31. Kitada T, Asakawa S, Hattori N, Matsumine H, Yamamura Y, Minoshima S, et al. Mutations in the parkin gene cause autosomal recessive juvenile parkinsonism. Nature. 1998;392(6676):605-8.
32. Lucking CB, Abbas N, Durr A, Bonifati V, Bonnet AM, de Broucker T, et al. Homozygous deletions in parkin gene in European and North African families with autosomal recessive juvenile parkinsonism. The European Consortium on Genetic Susceptibility in Parkinson's Disease and the French Parkinson's Disease Genetics Study Group. Lancet (London, England). 1998;352(9137):1355-6.
33. Hattori N, Kitada T, Matsumine H, Asakawa S, Yamamura Y, Yoshino H, et al. Molecular genetic analysis of a novel Parkin gene in Japanese families with autosomal recessive juvenile parkinsonism: evidence for variable homozygous deletions in the Parkin gene in affected individuals. Annals of neurology. 1998;44(6):935-41.
34. Ahmed MR, Zhan X, Song X, Kook S, Gurevich VV, Gurevich EV. Ubiquitin ligase parkin promotes Mdm2-arrestin interaction but inhibits arrestin ubiquitination. Biochemistry. 2011;50(18):3749-63.
35. Okada M, Jang SW, Ye K. Akt phosphorylation and nuclear phosphoinositide association mediate mRNA export and cell proliferation activities by ALY. Proceedings of the National Academy of Sciences of the United States of America. 2008;105(25):8649-54.
36. Bandyopadhyay S, Chiang CY, Srivastava J, Gersten M, White S, Bell R, et al. A human MAP kinase interactome. Nature methods. 2010;7(10):801-5.
37. Paisan-Ruiz C, Jain S, Evans EW, Gilks WP, Simon J, van der Brug M, et al. Cloning of the gene containing mutations that cause PARK8-linked Parkinson's disease. Neuron. 2004;44(4):595-600.
38. Zimprich A, Biskup S, Leitner P, Lichtner P, Farrer M, Lincoln S, et al. Mutations in LRRK2 cause autosomal-dominant parkinsonism with pleomorphic pathology. Neuron. 2004;44(4):601-7.
39. Nichols WC, Pankratz N, Hernandez D, Paisan-Ruiz C, Jain S, Halter CA, et al. Genetic screening for a single common LRRK2 mutation in familial Parkinson's disease. Lancet (London, England). 2005;365(9457):410-2.
40. Di Fonzo A, Rohe CF, Ferreira J, Chien HF, Vacca L, Stocchi F, et al. A frequent LRRK2 gene mutation associated with autosomal dominant Parkinson's disease. Lancet (London, England). 2005;365(9457):412-5.
41. Gilks WP, Abou-Sleiman PM, Gandhi S, Jain S, Singleton A, Lees AJ, et al. A common LRRK2 mutation in idiopathic Parkinson's disease. Lancet (London, England). 2005;365(9457):415-6.
42. Meixner A, Boldt K, Van Troys M, Askenazi M, Gloeckner CJ, Bauer M, et al. A QUICK screen for Lrrk2 interaction partners--leucine-rich repeat kinase 2 is involved in actin cytoskeleton dynamics. Molecular & cellular proteomics : MCP. 2011;10(1):M110.001172.
43. Zanon A, Rakovic A, Blankenburg H, Doncheva NT, Schwienbacher C, Serafin A, et al. Profiling of Parkin-binding partners using tandem affinity purification. PloS one. 2013;8(11):e78648.

44. Fogeron ML, Muller H, Schade S, Dreher F, Lehmann V, Kuhnel A, et al. LGALS3BP regulates centriole biogenesis and centrosome hypertrophy in cancer cells. Nature communications. 2013;4:1531.
45. Comer KA, Dennis PA, Armstrong L, Catino JJ, Kastan MB, Kumar CC. Human smooth muscle alpha-actin gene is a transcriptional target of the p53 tumor suppressor protein. Oncogene. 1998;16(10):1299-308.
46. Fontemaggi G, Kela I, Amariglio N, Rechavi G, Krishnamurthy J, Strano S, et al. Identification of direct p73 target genes combining DNA microarray and chromatin immunoprecipitation analyses. The Journal of biological chemistry. 2002;277(45):43359-68.
47. Reyniers L, Del Giudice MG, Civiero L, Belluzzi E, Lobbestael E, Beilina A, et al. Differential protein-protein interactions of LRRK1 and LRRK2 indicate roles in distinct cellular signaling pathways. Journal of neurochemistry. 2014;131(2):239-50.
48. Joberty G, Perlungher RR, Macara IG. The Borgs, a new family of Cdc42 and TC10 GTPase-interacting proteins. Molecular and cellular biology. 1999;19(10):6585-97.
49. Hirsch DS, Pirone DM, Burbelo PD. A new family of Cdc42 effector proteins, CEPs, function in fibroblast and epithelial cell shape changes. The Journal of biological chemistry. 2001;276(2):875-83.
50. Polymeropoulos MH, Lavedan C, Leroy E, Ide SE, Dehejia A, Dutra A, et al. Mutation in the alpha-synuclein gene identified in families with Parkinson's disease. Science (New York, NY). 1997;276(5321):2045-7.
51. Kruger R, Kuhn W, Muller T, Woitalla D, Graeber M, Kosel S, et al. Ala30Pro mutation in the gene encoding alpha-synuclein in Parkinson's disease. Nature genetics. 1998;18(2):106-8.
52. Singleton AB, Farrer M, Johnson J, Singleton A, Hague S, Kachergus J, et al. alpha-Synuclein locus triplication causes Parkinson's disease. Science (New York, NY). 2003;302(5646):841.
53. Chartier-Harlin MC, Kachergus J, Roumier C, Mouroux V, Douay X, Lincoln S, et al. Alpha-synuclein locus duplication as a cause of familial Parkinson's disease. Lancet (London, England). 2004;364(9440):1167-9.
54. Ibanez P, Bonnet AM, Debarges B, Lohmann E, Tison F, Pollak P, et al. Causal relation between alpha-synuclein gene duplication and familial Parkinson's disease. Lancet (London, England). 2004;364(9440):1169-71.
55. Schnack C, Danzer KM, Hengerer B, Gillardon F. Protein array analysis of oligomerization-induced changes in alpha-synuclein protein-protein interactions points to an interference with Cdc42 effector proteins. Neuroscience. 2008;154(4):1450-7.
56. Santini E, Valjent E, Usiello A, Carta M, Borgkvist A, Girault JA, et al. Critical involvement of cAMP/DARPP-32 and extracellular signal-regulated protein kinase signaling in L-DOPA-induced dyskinesia. The Journal of neuroscience : the official journal of the Society for Neuroscience. 2007;27(26):6995-7005.
57. Al-Alwan LA, Chang Y, Rousseau S, Martin JG, Eidelman DH, Hamid Q. CXCL1 inhibits airway smooth muscle cell migration through the decoy receptor Duffy antigen receptor for chemokines. Journal of immunology (Baltimore, Md : 1950). 2014;193(3):1416-26.
58. Spinella F, Garrafa E, Di Castro V, Rosano L, Nicotra MR, Caruso A, et al. Endothelin-1 stimulates lymphatic endothelial cells and lymphatic vessels to grow and invade. Cancer research. 2009;69(6):2669-76.
59. Imai Y, Soda M, Inoue H, Hattori N, Mizuno Y, Takahashi R. An unfolded putative transmembrane polypeptide, which can lead to endoplasmic reticulum stress, is a substrate of Parkin. Cell. 2001;105(7):891-902.
60. Letourneux C, Rocher G, Porteu F. B56-containing PP2A dephosphorylate ERK and their activity is controlled by the early gene IEX-1 and ERK. The EMBO journal. 2006;25(4):727-38.
61. Poy MN, Ruch RJ, Fernstrom MA, Okabayashi Y, Najjar SM. Shc and CEACAM1 interact to regulate the mitogenic action of insulin. The Journal of biological chemistry. 2002;277(2):1076-84.
62. Cheng J, Watkins SC, Walker WH. Testosterone activates mitogen-activated protein kinase via Src kinase and the epidermal growth factor receptor in sertoli cells. Endocrinology. 2007;148(5):2066-74.
63. Persons DL, Yazlovitskaya EM, Pelling JC. Effect of extracellular signal-regulated kinase on p53 accumulation in response to cisplatin. The Journal of biological chemistry. 2000;275(46):35778-85.
64. Lin J, Tang H, Jin X, Jia G, Hsieh JT. p53 regulates Stat3 phosphorylation and DNA binding activity in human prostate cancer cells expressing constitutively active Stat3. Oncogene. 2002;21(19):3082-8.
65. Nair VD, Yuen T, Olanow CW, Sealfon SC. Early single cell bifurcation of pro- and antiapoptotic states during oxidative stress. The Journal of biological chemistry. 2004;279(26):27494-501.

66. Falco A, Festa M, Basile A, Rosati A, Pascale M, Florenzano F, et al. BAG3 controls angiogenesis through regulation of ERK phosphorylation. Oncogene. 2012;31(50):5153-61.
67. Arora D, Kothe S, van den Eijnden M, Hooft van Huijsduijnen R, Heidel F, Fischer T, et al. Expression of protein-tyrosine phosphatases in Acute Myeloid Leukemia cells: FLT3 ITD sustains high levels of DUSP6 expression. Cell communication and signaling : CCS. 2012;10(1):19.
68. VanArsdall JE, Nairne JS, Pandeirada JN, Blunt JR. Adaptive memory: animacy processing produces mnemonic advantages. Experimental psychology. 2013;60(3):172-8.
69. Bagnyukova TV, Restifo D, Beeharry N, Gabitova L, Li T, Serebriiskii IG, et al. DUSP6 regulates drug sensitivity by modulating DNA damage response. British journal of cancer. 2013;109(4):1063-71.
70. Petersen PS, Jin C, Madsen AN, Rasmussen M, Kuhre R, Egerod KL, et al. Deficiency of the GPR39 receptor is associated with obesity and altered adipocyte metabolism. FASEB journal : official publication of the Federation of American Societies for Experimental Biology. 2011;25(11):3803-14.
71. Schilling D, Pittelkow MR, Kumar R. IEX-1, an immediate early gene, increases the rate of apoptosis in keratinocytes. Oncogene. 2001;20(55):7992-7.
72. Henry H, Thomas A, Shen Y, White E. Regulation of the mitochondrial checkpoint in p53-mediated apoptosis confers resistance to cell death. Oncogene. 2002;21(5):748-60.
73. Murayama Y, Miyagawa J, Oritani K, Yoshida H, Yamamoto K, Kishida O, et al. CD9-mediated activation of the p46 Shc isoform leads to apoptosis in cancer cells. Journal of cell science. 2004;117(Pt 15):3379-88.
74. Sun X, Wang Y, Zhang J, Tu J, Wang XJ, Su XD, et al. Tunneling-nanotube direction determination in neurons and astrocytes. Cell death & disease. 2012;3:e438.
75. Tatton NA. Increased caspase 3 and Bax immunoreactivity accompany nuclear GAPDH translocation and neuronal apoptosis in Parkinson's disease. Experimental neurology. 2000;166(1):29-43.
76. Mogi M, Togari A, Kondo T, Mizuno Y, Komure O, Kuno S, et al. Caspase activities and tumor necrosis factor receptor R1 (p55) level are elevated in the substantia nigra from parkinsonian brain. Journal of neural transmission (Vienna, Austria : 1996). 2000;107(3):335-41.
77. Osawa Y, Nagaki M, Banno Y, Brenner DA, Nozawa Y, Moriwaki H, et al. Expression of the NF-kappa B target gene X-ray-inducible immediate early response factor-1 short enhances TNF-alpha-induced hepatocyte apoptosis by inhibiting Akt activation. Journal of immunology (Baltimore, Md : 1950). 2003;170(8):4053-60.
78. Horikawa I, Fujita K, Jenkins LM, Hiyoshi Y, Mondal AM, Vojtesek B, et al. Autophagic degradation of the inhibitory p53 isoform Delta133p53alpha as a regulatory mechanism for p53-mediated senescence. Nature communications. 2014;5:4706.
79. Fan J, Ren H, Jia N, Fei E, Zhou T, Jiang P, et al. DJ-1 decreases Bax expression through repressing p53 transcriptional activity. The Journal of biological chemistry. 2008;283(7):4022-30.
80. Giaime E, Sunyach C, Druon C, Scarzello S, Robert G, Grosso S, et al. Loss of function of DJ-1 triggered by Parkinson's disease-associated mutation is due to proteolytic resistance to caspase-6. Cell death and differentiation. 2010;17(1):158-69.
81. Vasseur S, Afzal S, Tomasini R, Guillaumond F, Tardivel-Lacombe J, Mak TW, et al. Consequences of DJ-1 upregulation following p53 loss and cell transformation. Oncogene. 2012;31(5):664-70.
82. Zhang H, Chi Y, Gao K, Zhang X, Yao J. p53 protein-mediated up-regulation of MAP kinase phosphatase 3 (MKP-3) contributes to the establishment of the cellular senescent phenotype through dephosphorylation of extracellular signal-regulated kinase 1/2 (ERK1/2). The Journal of biological chemistry. 2015;290(2):1129-40.
83. Murphy SH, Suzuki K, Downes M, Welch GL, De Jesus P, Miraglia LJ, et al. Tumor suppressor protein (p)53, is a regulator of NF-kappaB repression by the glucocorticoid receptor. Proceedings of the National Academy of Sciences of the United States of America. 2011;108(41):17117-22.
84. Burnum KE, Hirota Y, Baker ES, Yoshie M, Ibrahim YM, Monroe ME, et al. Uterine deletion of Trp53 compromises antioxidant responses in the mouse decidua. Endocrinology. 2012;153(9):4568-79.
85. Boiko AD, Porteous S, Razorenova OV, Krivokrysenko VI, Williams BR, Gudkov AV. A systematic search for downstream mediators of tumor suppressor function of p53 reveals a major role of BTG2 in suppression of Ras-induced transformation. Genes & development. 2006;20(2):236-52.
86. Schafer H, Trauzold A, Sebens T, Deppert W, Folsch UR, Schmidt WE. The proliferation-associated early response gene p22/PRG1 is a novel p53 target gene. Oncogene. 1998;16(19):2479-87.
87. Huang YH, Wu JY, Zhang Y, Wu MX. Synergistic and opposing regulation of the stress-responsive gene IEX-1 by p53, c-Myc, and multiple NF-kappaB/rel complexes. Oncogene. 2002;21(44):6819-28.

88. Jin H, Suh DS, Kim TH, Yeom JH, Lee K, Bae J. IER3 is a crucial mediator of TAp73beta-induced apoptosis in cervical cancer and confers etoposide sensitivity. Scientific reports. 2015;5:8367.
89. Martin KR, Barrett JC. Reactive oxygen species as double-edged swords in cellular processes: low-dose cell signaling versus high-dose toxicity. Human & experimental toxicology. 2002;21(2):71-5.
90. Russell EG, Cotter TG. New Insight into the Role of Reactive Oxygen Species (ROS) in Cellular Signal-Transduction Processes. International review of cell and molecular biology. 2015;319:221-54.
91. Sies H, de Groot H. Role of reactive oxygen species in cell toxicity. Toxicology letters. 1992;64-65 Spec No:547-51.
92. Han Y, Chen JZ. Oxidative stress induces mitochondrial DNA damage and cytotoxicity through independent mechanisms in human cancer cells. BioMed research international. 2013;2013:825065.
93. Kim JJ, Lee SB, Park JK, Yoo YD. TNF-alpha-induced ROS production triggering apoptosis is directly linked to Romo1 and Bcl-X(L). Cell death and differentiation. 2010;17(9):1420-34.
94. Kakhlon O, Gruenbaum Y, Cabantchik ZI. Repression of ferritin expression increases the labile iron pool, oxidative stress, and short-term growth of human erythroleukemia cells. Blood. 2001;97(9):2863-71.
95. Fan GC, Zhou X, Wang X, Song G, Qian J, Nicolaou P, et al. Heat shock protein 20 interacting with phosphorylated Akt reduces doxorubicin-triggered oxidative stress and cardiotoxicity. Circulation research. 2008;103(11):1270-9.
96. Bruinsma IB, Bruggink KA, Kinast K, Versleijen AA, Segers-Nolten IM, Subramaniam V, et al. Inhibition of alpha-synuclein aggregation by small heat shock proteins. Proteins. 2011;79(10):2956-67.
97. Islamovic E, Duncan A, Bers DM, Gerthoffer WT, Mestril R. Importance of small heat shock protein 20 (hsp20) C-terminal extension in cardioprotection. Journal of molecular and cellular cardiology. 2007;42(4):862-9.
98. Mattson MP. Calcium and neurodegeneration. Aging cell. 2007;6(3):337-50.
99. Nose A, Mori Y, Uchiyama-Tanaka Y, Kishimoto N, Maruyama K, Matsubara H, et al. Regulation of glucose transporter (GLUT1) gene expression by angiotensin II in mesangial cells: involvement of HB-EGF and EGF receptor transactivation. Hypertension research : official journal of the Japanese Society of Hypertension. 2003;26(1):67-73.
100. Eggers C, Hilker R, Burghaus L, Schumacher B, Heiss WD. High resolution positron emission tomography demonstrates basal ganglia dysfunction in early Parkinson's disease. Journal of the neurological sciences. 2009;276(1-2):27-30.
101. Heilig C, Brosius F, Siu B, Concepcion L, Mortensen R, Heilig K, et al. Implications of glucose transporter protein type 1 (GLUT1)-haplodeficiency in embryonic stem cells for their survival in response to hypoxic stress. The American journal of pathology. 2003;163(5):1873-85.
102. Barthel A, Okino ST, Liao J, Nakatani K, Li J, Whitlock JP, Jr., et al. Regulation of GLUT1 gene transcription by the serine/threonine kinase Akt1. The Journal of biological chemistry. 1999;274(29):20281-6.
103. Edinger AL, Thompson CB. Akt maintains cell size and survival by increasing mTOR-dependent nutrient uptake. Molecular biology of the cell. 2002;13(7):2276-88.
104. Wieman HL, Wofford JA, Rathmell JC. Cytokine stimulation promotes glucose uptake via phosphatidylinositol-3 kinase/Akt regulation of Glut1 activity and trafficking. Molecular biology of the cell. 2007;18(4):1437-46.
105. Valente EM, Abou-Sleiman PM, Caputo V, Muqit MM, Harvey K, Gispert S, et al. Hereditary early-onset Parkinson's disease caused by mutations in PINK1. Science (New York, NY). 2004;304(5674):1158-60.
106. Hatano Y, Li Y, Sato K, Asakawa S, Yamamura Y, Tomiyama H, et al. Novel PINK1 mutations in early-onset parkinsonism. Annals of neurology. 2004;56(3):424-7.
107. Li Y, Tomiyama H, Sato K, Hatano Y, Yoshino H, Atsumi M, et al. Clinicogenetic study of PINK1 mutations in autosomal recessive early-onset parkinsonism. Neurology. 2005;64(11):1955-7.
108. Lin W, Wadlington NL, Chen L, Zhuang X, Brorson JR, Kang UJ. Loss of PINK1 attenuates HIF-1alpha induction by preventing 4E-BP1-dependent switch in protein translation under hypoxia. The Journal of neuroscience : the official journal of the Society for Neuroscience. 2014;34(8):3079-89.
109. Daoud SS, Munson PJ, Reinhold W, Young L, Prabhu VV, Yu Q, et al. Impact of p53 knockout and topotecan treatment on gene expression profiles in human colon carcinoma cells: a pharmacogenomic study. Cancer research. 2003;63(11):2782-93.
110. Schwartzenberg-Bar-Yoseph F, Armoni M, Karnieli E. The tumor suppressor p53 down-regulates glucose transporters GLUT1 and GLUT4 gene expression. Cancer research. 2004;64(7):2627-33.

111. Zawacka-Pankau J, Grinkevich VV, Hunten S, Nikulenkov F, Gluch A, Li H, et al. Inhibition of glycolytic enzymes mediated by pharmacologically activated p53: targeting Warburg effect to fight cancer. The Journal of biological chemistry. 2011;286(48):41600-15.
112. Yang C, Watson RT, Elmendorf JS, Sacks DB, Pessin JE. Calmodulin antagonists inhibit insulin-stimulated GLUT4 (glucose transporter 4) translocation by preventing the formation of phosphatidylinositol 3,4,5-trisphosphate in 3T3L1 adipocytes. Molecular endocrinology (Baltimore, Md). 2000;14(2):317-26.
113. Summers SA, Yin VP, Whiteman EL, Garza LA, Cho H, Tuttle RL, et al. Signaling pathways mediating insulin-stimulated glucose transport. Annals of the New York Academy of Sciences. 1999;892:169-86.
114. Karagiannides I, Stavrakis D, Bakirtzi K, Kokkotou E, Pirtskhalava T, Nayeb-Hashemi H, et al. Substance P (SP)-neurokinin-1 receptor (NK-1R) alters adipose tissue responses to high-fat diet and insulin action. Endocrinology. 2011;152(6):2197-205.
115. Karagiannides I, Bakirtzi K, Kokkotou E, Stavrakis D, Margolis KG, Thomou T, et al. Role of substance P in the regulation of glucose metabolism via insulin signaling-associated pathways. Endocrinology. 2011;152(12):4571-80.
116. Gullner HG, Yajima H, Harris V, Unger RH. Kassinin: stimulation of insulin and glucagon secretion in the rat. Endocrinology. 1982;110(4):1246-8.
117. Andreozzi F, D'Alessandris C, Federici M, Laratta E, Del Guerra S, Del Prato S, et al. Activation of the hexosamine pathway leads to phosphorylation of insulin receptor substrate-1 on Ser307 and Ser612 and impairs the phosphatidylinositol 3-kinase/Akt/mammalian target of rapamycin insulin biosynthetic pathway in RIN pancreatic beta-cells. Endocrinology. 2004;145(6):2845-57.
118. Lawrence MC, McGlynn K, Park BH, Cobb MH. ERK1/2-dependent activation of transcription factors required for acute and chronic effects of glucose on the insulin gene promoter. The Journal of biological chemistry. 2005;280(29):26751-9.
119. Nevins AK, Thurmond DC. A direct interaction between Cdc42 and vesicle-associated membrane protein 2 regulates SNARE-dependent insulin exocytosis. The Journal of biological chemistry. 2005;280(3):1944-52.
120. Kepner EM, Yoder SM, Oh E, Kalwat MA, Wang Z, Quilliam LA, et al. Cool-1/betaPIX functions as a guanine nucleotide exchange factor in the cycling of Cdc42 to regulate insulin secretion. American journal of physiology Endocrinology and metabolism. 2011;301(6):E1072-80.
121. Lee S, Zhang C, Liu X. Role of glucose metabolism and ATP in maintaining PINK1 levels during Parkin-mediated mitochondrial damage responses. The Journal of biological chemistry. 2015;290(2):904-17.
122. Li X, Li MR, Guo ZX. Effects of adiponectin on oxidative stress and apoptosis in human cardiac myocytes cultured with high glucose. Chinese medical journal. 2012;125(23):4209-13.
123. Carmeliet P, Dor Y, Herbert JM, Fukumura D, Brusselmans K, Dewerchin M, et al. Role of HIF-1alpha in hypoxia-mediated apoptosis, cell proliferation and tumour angiogenesis. Nature. 1998;394(6692):485-90.
124. Okoshi R, Ozaki T, Yamamoto H, Ando K, Koida N, Ono S, et al. Activation of AMP-activated protein kinase induces p53-dependent apoptotic cell death in response to energetic stress. The Journal of biological chemistry. 2008;283(7):3979-87.
125. Chen ZJ, Huang SM, Fan WX, Tang WX, Liu F, Qiu HY. [Effects of high glucose on expression of core binding factor alpha1 and osteocalcin in vascular smooth muscle cells]. Sichuan da xue xue bao Yi xue ban = Journal of Sichuan University Medical science edition. 2010;41(5):784-8.
126. Khan JA, Amazit L, Bellance C, Guiochon-Mantel A, Lombes M, Loosfelt H. p38 and p42/44 MAPKs differentially regulate progesterone receptor A and B isoform stabilization. Molecular endocrinology (Baltimore, Md). 2011;25(10):1710-24.
127. Mistafa O, Ghalali A, Kadekar S, Hogberg J, Stenius U. Purinergic receptor-mediated rapid depletion of nuclear phosphorylated Akt depends on pleckstrin homology domain leucine-rich repeat phosphatase, calcineurin, protein phosphatase 2A, and PTEN phosphatases. The Journal of biological chemistry. 2010;285(36):27900-10.
128. Chahdi A, Sorokin A. Endothelin-1 induces p66Shc activation through EGF receptor transactivation: Role of beta(1)Pix/Galpha(i3) interaction. Cellular signalling. 2010;22(2):325-9.
129. Ziegelbauer J, Wei J, Tjian R. Myc-interacting protein 1 target gene profile: a link to microtubules, extracellular signal-regulated kinase, and cell growth. Proceedings of the National Academy of Sciences of the United States of America. 2004;101(2):458-63.
130. Zhao L, Brinton RD. Vasopressin-induced cytoplasmic and nuclear calcium signaling in embryonic cortical astrocytes: dynamics of calcium and calcium-dependent kinase translocation. The Journal of neuroscience : the official journal of the Society for Neuroscience. 2003;23(10):4228-39.

131. Kawasaki Y, Kohno T, Zhuang ZY, Brenner GJ, Wang H, Van Der Meer C, et al. Ionotropic and metabotropic receptors, protein kinase A, protein kinase C, and Src contribute to C-fiber-induced ERK activation and cAMP response element-binding protein phosphorylation in dorsal horn neurons, leading to central sensitization. The Journal of neuroscience : the official journal of the Society for Neuroscience. 2004;24(38):8310-21.
132. He Z, Jiang J, Kokkinaki M, Golestaneh N, Hofmann MC, Dym M. Gdnf upregulates c-Fos transcription via the Ras/Erk1/2 pathway to promote mouse spermatogonial stem cell proliferation. Stem cells (Dayton, Ohio). 2008;26(1):266-78.
133. Liu N, Cigola E, Tinti C, Jin BK, Conti B, Volpe BT, et al. Unique regulation of immediate early gene and tyrosine hydroxylase expression in the odor-deprived mouse olfactory bulb. The Journal of biological chemistry. 1999;274(5):3042-7.
134. Suzuki T, Yamakuni T, Hagiwara M, Ichinose H. Identification of ATF-2 as a transcriptional regulator for the tyrosine hydroxylase gene. The Journal of biological chemistry. 2002;277(43):40768-74.
135. Svenningsson P, Nishi A, Fisone G, Girault JA, Nairn AC, Greengard P. DARPP-32: an integrator of neurotransmission. Annual review of pharmacology and toxicology. 2004;44:269-96.
136. Fernandez E, Schiappa R, Girault JA, Le Novere N. DARPP-32 is a robust integrator of dopamine and glutamate signals. PLoS computational biology. 2006;2(12):e176.
137. Yan Z, Feng J, Fienberg AA, Greengard P. D(2) dopamine receptors induce mitogen-activated protein kinase and cAMP response element-binding protein phosphorylation in neurons. Proceedings of the National Academy of Sciences of the United States of America. 1999;96(20):11607-12.
138. Gu L, Waliany S, Kane SE. Darpp-32 and its truncated variant t-Darpp have antagonistic effects on breast cancer cell growth and herceptin resistance. PloS one. 2009;4(7):e6220.
139. Westerlund M, Ran C, Borgkvist A, Sterky FH, Lindqvist E, Lundstromer K, et al. Lrrk2 and alpha-synuclein are co-regulated in rodent striatum. Molecular and cellular neurosciences. 2008;39(4):586-91.
140. Belkhiri A, Dar AA, Zaika A, Kelley M, El-Rifai W. t-Darpp promotes cancer cell survival by up-regulation of Bcl2 through Akt-dependent mechanism. Cancer research. 2008;68(2):395-403.
141. Liu F, Ma XH, Ule J, Bibb JA, Nishi A, DeMaggio AJ, et al. Regulation of cyclin-dependent kinase 5 and casein kinase 1 by metabotropic glutamate receptors. Proceedings of the National Academy of Sciences of the United States of America. 2001;98(20):11062-8.
142. Bolick DT, Hatley ME, Srinivasan S, Hedrick CC, Nadler JL. Lisofylline, a novel antiinflammatory compound, protects mesangial cells from hyperglycemia- and angiotensin II-mediated extracellular matrix deposition. Endocrinology. 2003;144(12):5227-31.
143. Tao X, Finkbeiner S, Arnold DB, Shaywitz AJ, Greenberg ME. Ca2+ influx regulates BDNF transcription by a CREB family transcription factor-dependent mechanism. Neuron. 1998;20(4):709-26.
144. Datta SR, Brunet A, Greenberg ME. Cellular survival: a play in three Akts. Genes & development. 1999;13(22):2905-27.
145. Pugazhenthi S, Nesterova A, Sable C, Heidenreich KA, Boxer LM, Heasley LE, et al. Akt/protein kinase B up-regulates Bcl-2 expression through cAMP-response element-binding protein. The Journal of biological chemistry. 2000;275(15):10761-6.
146. D'Amico M, Hulit J, Amanatullah DF, Zafonte BT, Albanese C, Bouzahzah B, et al. The integrin-linked kinase regulates the cyclin D1 gene through glycogen synthase kinase 3beta and cAMP-responsive element-binding protein-dependent pathways. The Journal of biological chemistry. 2000;275(42):32649-57.
147. Hayakawa J, Ohmichi M, Tasaka K, Kanda Y, Adachi K, Nishio Y, et al. Regulation of the PRL promoter by Akt through cAMP response element binding protein. Endocrinology. 2002;143(1):13-22.
148. Giebler HA, Lemasson I, Nyborg JK. p53 recruitment of CREB binding protein mediated through phosphorylated CREB: a novel pathway of tumor suppressor regulation. Molecular and cellular biology. 2000;20(13):4849-58.
149. Raymond J. Science in the service of medicine: germ theory, bacteriology and English public health, 1860-1914. The Society for the Social History of Medicine bulletin. 1985;37:43-5.
150. Okoshi R, Kubo N, Nakashima K, Shimozato O, Nakagawara A, Ozaki T. CREB represses p53-dependent transactivation of MDM2 through the complex formation with p53 and contributes to p53-mediated apoptosis in response to glucose deprivation. Biochemical and biophysical research communications. 2011;406(1):79-84.
151. Virbasius CM, Wagner S, Green MR. A human nuclear-localized chaperone that regulates dimerization, DNA binding, and transcriptional activity of bZIP proteins. Molecular cell. 1999;4(2):219-28.

152. Benito E, Valor LM, Jimenez-Minchan M, Huber W, Barco A. cAMP response element-binding protein is a primary hub of activity-driven neuronal gene expression. The Journal of neuroscience : the official journal of the Society for Neuroscience. 2011;31(50):18237-50.
153. Ondo WG, Dat Vuong K, Khan H, Atassi F, Kwak C, Jankovic J. Daytime sleepiness and other sleep disorders in Parkinson's disease. Neurology. 2001;57(8):1392-6.
154. Hogl B, Seppi K, Brandauer E, Glatzl S, Frauscher B, Niedermuller U, et al. Increased daytime sleepiness in Parkinson's disease: a questionnaire survey. Movement disorders : official journal of the Movement Disorder Society. 2003;18(3):319-23.
155. Cai Y, Liu S, Sothern RB, Xu S, Chan P. Expression of clock genes Per1 and Bmal1 in total leukocytes in health and Parkinson's disease. European journal of neurology. 2010;17(4):550-4.
156. Lin Q, Ding H, Zheng Z, Gu Z, Ma J, Chen L, et al. Promoter methylation analysis of seven clock genes in Parkinson's disease. Neuroscience letters. 2012;507(2):147-50.
157. Bolitho SJ, Naismith SL, Rajaratnam SM, Grunstein RR, Hodges JR, Terpening Z, et al. Disturbances in melatonin secretion and circadian sleep-wake regulation in Parkinson disease. Sleep medicine. 2014;15(3):342-7.
158. Videnovic A, Noble C, Reid KJ, Peng J, Turek FW, Marconi A, et al. Circadian melatonin rhythm and excessive daytime sleepiness in Parkinson disease. JAMA neurology. 2014;71(4):463-9.
159. Breen DP, Vuono R, Nawarathna U, Fisher K, Shneerson JM, Reddy AB, et al. Sleep and circadian rhythm regulation in early Parkinson disease. JAMA neurology. 2014;71(5):589-95.
160. Tanaka M, Yamaguchi E, Takahashi M, Hashimura K, Shibata T, Nakamura W, et al. Effects of age-related dopaminergic neuron loss in the substantia nigra on the circadian rhythms of locomotor activity in mice. Neuroscience research. 2012;74(3-4):210-5.
161. Travnickova-Bendova Z, Cermakian N, Reppert SM, Sassone-Corsi P. Bimodal regulation of mPeriod promoters by CREB-dependent signaling and CLOCK/BMAL1 activity. Proceedings of the National Academy of Sciences of the United States of America. 2002;99(11):7728-33.
162. Lemberger T, Parkitna JR, Chai M, Schutz G, Engblom D. CREB has a context-dependent role in activity-regulated transcription and maintains neuronal cholesterol homeostasis. FASEB journal : official publication of the Federation of American Societies for Experimental Biology. 2008;22(8):2872-9.
163. Balsalobre A, Marcacci L, Schibler U. Multiple signaling pathways elicit circadian gene expression in cultured Rat-1 fibroblasts. Current biology : CB. 2000;10(20):1291-4.
164. Miki T, Matsumoto T, Zhao Z, Lee CC. p53 regulates Period2 expression and the circadian clock. Nature communications. 2013;4:2444.
165. Yamaguchi S, Mitsui S, Yan L, Yagita K, Miyake S, Okamura H. Role of DBP in the circadian oscillatory mechanism. Molecular and cellular biology. 2000;20(13):4773-81.
166. Jin CM, Yang YJ, Huang HS, Kai M, Lee MK. Mechanisms of L-DOPA-induced cytotoxicity in rat adrenal pheochromocytoma cells: implication of oxidative stress-related kinases and cyclic AMP. Neuroscience. 2010;170(2):390-8.
167. Alcacer C, Santini E, Valjent E, Gaven F, Girault JA, Herve D. Galpha(olf) mutation allows parsing the role of cAMP-dependent and extracellular signal-regulated kinase-dependent signaling in L-3,4-dihydroxyphenylalanine-induced dyskinesia. The Journal of neuroscience : the official journal of the Society for Neuroscience. 2012;32(17):5900-10.
168. Cole DG, Kobierski LA, Konradi C, Hyman SE. 6-Hydroxydopamine lesions of rat substantia nigra up-regulate dopamine-induced phosphorylation of the cAMP-response element-binding protein in striatal neurons. Proceedings of the National Academy of Sciences of the United States of America. 1994;91(20):9631-5.
169. Liu WG, Chen Y, Li B, Lu GQ, Chen SD. Neuroprotection by pergolide against levodopa-induced cytotoxicity of neural stem cells. Neurochemical research. 2004;29(12):2207-14.
170. Nishi A, Kuroiwa M, Shuto T. Mechanisms for the modulation of dopamine d(1) receptor signaling in striatal neurons. Frontiers in neuroanatomy. 2011;5:43.
171. Yager LM, Garcia AF, Wunsch AM, Ferguson SM. The ins and outs of the striatum: role in drug addiction. Neuroscience. 2015;301:529-41.
172. Kaneko S, Hikida T, Watanabe D, Ichinose H, Nagatsu T, Kreitman RJ, et al. Synaptic integration mediated by striatal cholinergic interneurons in basal ganglia function. Science (New York, NY). 2000;289(5479):633-7.
173. Wang Z, Kai L, Day M, Ronesi J, Yin HH, Ding J, et al. Dopaminergic control of corticostriatal long-term synaptic depression in medium spiny neurons is mediated by cholinergic interneurons. Neuron. 2006;50(3):443-52.

174. Witten IB, Lin SC, Brodsky M, Prakash R, Diester I, Anikeeva P, et al. Cholinergic interneurons control local circuit activity and cocaine conditioning. Science (New York, NY). 2010;330(6011):1677-81.
175. Nelson AB, Hammack N, Yang CF, Shah NM, Seal RP, Kreitzer AC. Striatal cholinergic interneurons Drive GABA release from dopamine terminals. Neuron. 2014;82(1):63-70.
176. Zhou FM, Liang Y, Dani JA. Endogenous nicotinic cholinergic activity regulates dopamine release in the striatum. Nature neuroscience. 2001;4(12):1224-9.
177. Rahman S, McBride WJ. Involvement of GABA and cholinergic receptors in the nucleus accumbens on feedback control of somatodendritic dopamine release in the ventral tegmental area. Journal of neurochemistry. 2002;80(4):646-54.
178. Rice ME, Cragg SJ. Nicotine amplifies reward-related dopamine signals in striatum. Nature neuroscience. 2004;7(6):583-4.
179. Exley R, Cragg SJ. Presynaptic nicotinic receptors: a dynamic and diverse cholinergic filter of striatal dopamine neurotransmission. British journal of pharmacology. 2008;153 Suppl 1:S283-97.
180. Threlfell S, Clements MA, Khodai T, Pienaar IS, Exley R, Wess J, et al. Striatal muscarinic receptors promote activity dependence of dopamine transmission via distinct receptor subtypes on cholinergic interneurons in ventral versus dorsal striatum. The Journal of neuroscience : the official journal of the Society for Neuroscience. 2010;30(9):3398-408.
181. Higley MJ, Gittis AH, Oldenburg IA, Balthasar N, Seal RP, Edwards RH, et al. Cholinergic interneurons mediate fast VGluT3-dependent glutamatergic transmission in the striatum. PloS one. 2011;6(4):e19155.
182. Threlfell S, Lalic T, Platt NJ, Jennings KA, Deisseroth K, Cragg SJ. Striatal dopamine release is triggered by synchronized activity in cholinergic interneurons. Neuron. 2012;75(1):58-64.
183. Cachope R, Mateo Y, Mathur BN, Irving J, Wang HL, Morales M, et al. Selective activation of cholinergic interneurons enhances accumbal phasic dopamine release: setting the tone for reward processing. Cell reports. 2012;2(1):33-41.
184. Pisani A, Bernardi G, Ding J, Surmeier DJ. Re-emergence of striatal cholinergic interneurons in movement disorders. Trends in neurosciences. 2007;30(10):545-53.
185. Aosaki T, Graybiel AM, Kimura M. Effect of the nigrostriatal dopamine system on acquired neural responses in the striatum of behaving monkeys. Science (New York, NY). 1994;265(5170):412-5.
186. Graybiel AM, Aosaki T, Flaherty AW, Kimura M. The basal ganglia and adaptive motor control. Science (New York, NY). 1994;265(5180):1826-31.
187. Maurice N, Mercer J, Chan CS, Hernandez-Lopez S, Held J, Tkatch T, et al. D2 dopamine receptor-mediated modulation of voltage-dependent Na+ channels reduces autonomous activity in striatal cholinergic interneurons. The Journal of neuroscience : the official journal of the Society for Neuroscience. 2004;24(46):10289-301.
188. Martel P, Leo D, Fulton S, Berard M, Trudeau LE. Role of Kv1 potassium channels in regulating dopamine release and presynaptic D2 receptor function. PloS one. 2011;6(5):e20402.
189. Wilson CJ. The mechanism of intrinsic amplification of hyperpolarizations and spontaneous bursting in striatal cholinergic interneurons. Neuron. 2005;45(4):575-85.
190. Beatty JA, Sullivan MA, Morikawa H, Wilson CJ. Complex autonomous firing patterns of striatal low-threshold spike interneurons. Journal of neurophysiology. 2012;108(3):771-81.
191. Nakamura TY, Coetzee WA, Vega-Saenz De Miera E, Artman M, Rudy B. Modulation of Kv4 channels, key components of rat ventricular transient outward K+ current, by PKC. The American journal of physiology. 1997;273(4 Pt 2):H1775-86.
192. Shen W, Hernandez-Lopez S, Tkatch T, Held JE, Surmeier DJ. Kv1.2-containing K+ channels regulate subthreshold excitability of striatal medium spiny neurons. Journal of neurophysiology. 2004;91(3):1337-49.
193. Shen W, Hamilton SE, Nathanson NM, Surmeier DJ. Cholinergic suppression of KCNQ channel currents enhances excitability of striatal medium spiny neurons. The Journal of neuroscience : the official journal of the Society for Neuroscience. 2005;25(32):7449-58.
194. Shen W, Tian X, Day M, Ulrich S, Tkatch T, Nathanson NM, et al. Cholinergic modulation of Kir2 channels selectively elevates dendritic excitability in striatopallidal neurons. Nature neuroscience. 2007;10(11):1458-66.
195. Qian L, Huang Y, Spencer CI, Foley A, Vedantham V, Liu L, et al. In vivo reprogramming of murine cardiac fibroblasts into induced cardiomyocytes. Nature. 2012;485(7400):593-8.
196. Yang SH, Galanis A, Sharrocks AD. Targeting of p38 mitogen-activated protein kinases to MEF2 transcription factors. Molecular and cellular biology. 1999;19(6):4028-38.

197. Kyriakis JM, Avruch J. Mammalian mitogen-activated protein kinase signal transduction pathways activated by stress and inflammation. Physiological reviews. 2001;81(2):807-69.
198. Elmer BM, Estes ML, Barrow SL, McAllister AK. MHCI requires MEF2 transcription factors to negatively regulate synapse density during development and in disease. The Journal of neuroscience : the official journal of the Society for Neuroscience. 2013;33(34):13791-804.
199. Cavanaugh JE, Ham J, Hetman M, Poser S, Yan C, Xia Z. Differential regulation of mitogen-activated protein kinases ERK1/2 and ERK5 by neurotrophins, neuronal activity, and cAMP in neurons. The Journal of neuroscience : the official journal of the Society for Neuroscience. 2001;21(2):434-43.
200. Lyons MR, Schwarz CM, West AE. Members of the myocyte enhancer factor 2 transcription factor family differentially regulate Bdnf transcription in response to neuronal depolarization. The Journal of neuroscience : the official journal of the Society for Neuroscience. 2012;32(37):12780-5.
201. Parsian A, Sinha R, Racette B, Zhao JH, Perlmutter JS. Association of a variation in the promoter region of the brain-derived neurotrophic factor gene with familial Parkinson's disease. Parkinsonism & related disorders. 2004;10(4):213-9.
202. Guerini FR, Beghi E, Riboldazzi G, Zangaglia R, Pianezzola C, Bono G, et al. BDNF Val66Met polymorphism is associated with cognitive impairment in Italian patients with Parkinson's disease. European journal of neurology. 2009;16(11):1240-5.
203. Bialecka M, Kurzawski M, Roszmann A, Robowski P, Sitek EJ, Honczarenko K, et al. BDNF G196A (Val66Met) polymorphism associated with cognitive impairment in Parkinson's disease. Neuroscience letters. 2014;561:86-90.
204. Chen YA, Scales SJ, Patel SM, Doung YC, Scheller RH. SNARE complex formation is triggered by Ca2+ and drives membrane fusion. Cell. 1999;97(2):165-74.
205. Haucke V, Neher E, Sigrist SJ. Protein scaffolds in the coupling of synaptic exocytosis and endocytosis. Nature reviews Neuroscience. 2011;12(3):127-38.
206. Coppola T, Magnin-Luthi S, Perret-Menoud V, Gattesco S, Schiavo G, Regazzi R. Direct interaction of the Rab3 effector RIM with Ca2+ channels, SNAP-25, and synaptotagmin. The Journal of biological chemistry. 2001;276(35):32756-62.
207. Kaeser PS, Deng L, Wang Y, Dulubova I, Liu X, Rizo J, et al. RIM proteins tether Ca2+ channels to presynaptic active zones via a direct PDZ-domain interaction. Cell. 2011;144(2):282-95.
208. Muller CS, Haupt A, Bildl W, Schindler J, Knaus HG, Meissner M, et al. Quantitative proteomics of the Cav2 channel nano-environments in the mammalian brain. Proceedings of the National Academy of Sciences of the United States of America. 2010;107(34):14950-7.
209. Fan M, Buraei Z, Luo HR, Levenson-Palmer R, Yang J. Direct inhibition of P/Q-type voltage-gated Ca2+ channels by Gem does not require a direct Gem/Cavbeta interaction. Proceedings of the National Academy of Sciences of the United States of America. 2010;107(33):14887-92.
210. Gorini G, Ponomareva O, Shores KS, Person MD, Harris RA, Mayfield RD. Dynamin-1 co-associates with native mouse brain BKCa channels: proteomics analysis of synaptic protein complexes. FEBS letters. 2010;584(5):845-51.
211. Schollmeier Y, Krause JM, Kreye S, Malsam J, Sollner TH. Resolving the function of distinct Munc18-1/SNARE protein interaction modes in a reconstituted membrane fusion assay. The Journal of biological chemistry. 2011;286(35):30582-90.
212. Di Giovanni J, Iborra C, Maulet Y, Leveque C, El Far O, Seagar M. Calcium-dependent regulation of SNARE-mediated membrane fusion by calmodulin. The Journal of biological chemistry. 2010;285(31):23665-75.
213. Berggard T, Arrigoni G, Olsson O, Fex M, Linse S, James P. 140 mouse brain proteins identified by Ca2+-calmodulin affinity chromatography and tandem mass spectrometry. Journal of proteome research. 2006;5(3):669-87.
214. Verhage M, de Vries KJ, Roshol H, Burbach JP, Gispen WH, Sudhof TC. DOC2 proteins in rat brain: complementary distribution and proposed function as vesicular adapter proteins in early stages of secretion. Neuron. 1997;18(3):453-61.
215. Pang ZP, Xu W, Cao P, Sudhof TC. Calmodulin suppresses synaptotagmin-2 transcription in cortical neurons. The Journal of biological chemistry. 2010;285(44):33930-9.
216. Biederer T, Sudhof TC. Mints as adaptors. Direct binding to neurexins and recruitment of munc18. The Journal of biological chemistry. 2000;275(51):39803-6.
217. Ciufo LF, Barclay JW, Burgoyne RD, Morgan A. Munc18-1 regulates early and late stages of exocytosis via syntaxin-independent protein interactions. Molecular biology of the cell. 2005;16(2):470-82.

218. McFarland MA, Ellis CE, Markey SP, Nussbaum RL. Proteomics analysis identifies phosphorylation-dependent alpha-synuclein protein interactions. Molecular & cellular proteomics : MCP. 2008;7(11):2123-37.
219. Modregger J, Schmidt AA, Ritter B, Huttner WB, Plomann M. Characterization of Endophilin B1b, a brain-specific membrane-associated lysophosphatidic acid acyl transferase with properties distinct from endophilin A1. The Journal of biological chemistry. 2003;278(6):4160-7.
220. Cirnaru MD, Marte A, Belluzzi E, Russo I, Gabrielli M, Longo F, et al. LRRK2 kinase activity regulates synaptic vesicle trafficking and neurotransmitter release through modulation of LRRK2 macro-molecular complex. Frontiers in molecular neuroscience. 2014;7:49.
221. Shimura H, Schlossmacher MG, Hattori N, Frosch MP, Trockenbacher A, Schneider R, et al. Ubiquitination of a new form of alpha-synuclein by parkin from human brain: implications for Parkinson's disease. Science (New York, NY). 2001;293(5528):263-9.
222. Woods WS, Boettcher JM, Zhou DH, Kloepper KD, Hartman KL, Ladror DT, et al. Conformation-specific binding of alpha-synuclein to novel protein partners detected by phage display and NMR spectroscopy. The Journal of biological chemistry. 2007;282(47):34555-67.
223. Goold R, Baines AJ. Evidence that two non-overlapping high-affinity calmodulin-binding sites are present in the head region of synapsin I. European journal of biochemistry / FEBS. 1994;224(1):229-40.
224. Larson ME, Sherman MA, Greimel S, Kuskowski M, Schneider JA, Bennett DA, et al. Soluble alpha-synuclein is a novel modulator of Alzheimer's disease pathophysiology. The Journal of neuroscience : the official journal of the Society for Neuroscience. 2012;32(30):10253-66.
225. Shimokawa N, Haglund K, Holter SM, Grabbe C, Kirkin V, Koibuchi N, et al. CIN85 regulates dopamine receptor endocytosis and governs behaviour in mice. The EMBO journal. 2010;29(14):2421-32.
226. Trempe JF, Chen CX, Grenier K, Camacho EM, Kozlov G, McPherson PS, et al. SH3 domains from a subset of BAR proteins define a Ubl-binding domain and implicate parkin in synaptic ubiquitination. Molecular cell. 2009;36(6):1034-47.
227. Matta S, Van Kolen K, da Cunha R, van den Bogaart G, Mandemakers W, Miskiewicz K, et al. LRRK2 controls an EndoA phosphorylation cycle in synaptic endocytosis. Neuron. 2012;75(6):1008-21.
228. Kim JW, Kim DH, Kim SH, Cha JK. Association of the dopamine transporter gene with Parkinson's disease in Korean patients. Journal of Korean medical science. 2000;15(4):449-51.
229. Wang J, Liu Z, Chen B. [Association between genetic polymorphism of dopamine transporter gene and susceptibility to Parkinson's disease]. Zhonghua yi xue za zhi. 2000;80(5):346-8.
230. Zhai D, Li S, Zhao Y, Lin Z. SLC6A3 is a risk factor for Parkinson's disease: a meta-analysis of sixteen years' studies. Neuroscience letters. 2014;564:99-104.
231. Jones SR, Gainetdinov RR, Jaber M, Giros B, Wightman RM, Caron MG. Profound neuronal plasticity in response to inactivation of the dopamine transporter. Proceedings of the National Academy of Sciences of the United States of America. 1998;95(7):4029-34.
232. Perez RG, Waymire JC, Lin E, Liu JJ, Guo F, Zigmond MJ. A role for alpha-synuclein in the regulation of dopamine biosynthesis. The Journal of neuroscience : the official journal of the Society for Neuroscience. 2002;22(8):3090-9.
233. George RJ, Haycock JW, Johnston JP, Craviso GL, Waymire JC. In vitro phosphorylation of bovine adrenal chromaffin cell tyrosine hydroxylase by endogenous protein kinases. Journal of neurochemistry. 1989;52(1):274-84.
234. Iuvone PM. Calcium, ATP, and magnesium activate soluble tyrosine hydroxylase from rat striatum. Journal of neurochemistry. 1984;43(5):1359-68.
235. Volles MJ, Lansbury PT, Jr. Zeroing in on the pathogenic form of alpha-synuclein and its mechanism of neurotoxicity in Parkinson's disease. Biochemistry. 2003;42(26):7871-8.
236. Lindgren N, Usiello A, Goiny M, Haycock J, Erbs E, Greengard P, et al. Distinct roles of dopamine D2L and D2S receptor isoforms in the regulation of protein phosphorylation at presynaptic and postsynaptic sites. Proceedings of the National Academy of Sciences of the United States of America. 2003;100(7):4305-9.
237. Friedman WJ, Dreyfus CF, McEwen B, Black IB. Substance K (NKA) increases tyrosine hydroxylase mRNA in cultured substantia nigra. Brain research. 1988;427(2):203-5.
238. Hashimoto M, Rockenstein E, Mante M, Mallory M, Masliah E. beta-Synuclein inhibits alpha-synuclein aggregation: a possible role as an anti-parkinsonian factor. Neuron. 2001;32(2):213-23.
239. Hashimoto M, Bar-On P, Ho G, Takenouchi T, Rockenstein E, Crews L, et al. Beta-synuclein regulates Akt activity in neuronal cells. A possible mechanism for neuroprotection in Parkinson's disease. The Journal of biological chemistry. 2004;279(22):23622-9.

240. da Costa CA, Masliah E, Checler F. Beta-synuclein displays an antiapoptotic p53-dependent phenotype and protects neurons from 6-hydroxydopamine-induced caspase 3 activation: cross-talk with alpha-synuclein and implication for Parkinson's disease. The Journal of biological chemistry. 2003;278(39):37330-5.
241. Takahashi N, Miner LL, Sora I, Ujike H, Revay RS, Kostic V, et al. VMAT2 knockout mice: heterozygotes display reduced amphetamine-conditioned reward, enhanced amphetamine locomotion, and enhanced MPTP toxicity. Proceedings of the National Academy of Sciences of the United States of America. 1997;94(18):9938-43.
242. Glatt CE, Wahner AD, White DJ, Ruiz-Linares A, Ritz B. Gain-of-function haplotypes in the vesicular monoamine transporter promoter are protective for Parkinson disease in women. Human molecular genetics. 2006;15(2):299-305.
243. Brighina L, Riva C, Bertola F, Saracchi E, Fermi S, Goldwurm S, et al. Analysis of vesicular monoamine transporter 2 polymorphisms in Parkinson's disease. Neurobiology of aging. 2013;34(6):1712.e9-13.
244. Calabresi P, Picconi B, Tozzi A, Ghiglieri V, Di Filippo M. Direct and indirect pathways of basal ganglia: a critical reappraisal. Nature neuroscience. 2014;17(8):1022-30.
245. Grevle L, Guzey C, Hadidi H, Brennersted R, Idle JR, Aasly J. Allelic association between the DRD2 TaqI A polymorphism and Parkinson's disease. Movement disorders : official journal of the Movement Disorder Society. 2000;15(6):1070-4.
246. McGuire V, Van Den Eeden SK, Tanner CM, Kamel F, Umbach DM, Marder K, et al. Association of DRD2 and DRD3 polymorphisms with Parkinson's disease in a multiethnic consortium. Journal of the neurological sciences. 2011;307(1-2):22-9.
247. Hasbi A, Perreault ML, Shen MY, Zhang L, To R, Fan T, et al. A peptide targeting an interaction interface disrupts the dopamine D1-D2 receptor heteromer to block signaling and function in vitro and in vivo: effective selective antagonism. FASEB journal : official publication of the Federation of American Societies for Experimental Biology. 2014;28(11):4806-20.
248. Griffon N, Jeanneteau F, Prieur F, Diaz J, Sokoloff P. CLIC6, a member of the intracellular chloride channel family, interacts with dopamine D(2)-like receptors. Brain research Molecular brain research. 2003;117(1):47-57.
249. Hayes G, Biden TJ, Selbie LA, Shine J. Structural subtypes of the dopamine D2 receptor are functionally distinct: expression of the cloned D2A and D2B subtypes in a heterologous cell line. Molecular endocrinology (Baltimore, Md). 1992;6(6):920-6.
250. Lee SP, So CH, Rashid AJ, Varghese G, Cheng R, Lanca AJ, et al. Dopamine D1 and D2 receptor Co-activation generates a novel phospholipase C-mediated calcium signal. The Journal of biological chemistry. 2004;279(34):35671-8.
251. Heath MJ, Womack MD, MacDermott AB. Substance P elevates intracellular calcium in both neurons and glial cells from the dorsal horn of the spinal cord. Journal of neurophysiology. 1994;72(3):1192-8.
252. Tanabe T, Otani H, Zeng XT, Mishima K, Ogawa R, Inagaki C. Inhibitory effects of calcitonin gene-related peptide on substance-P-induced superoxide production in human neutrophils. European journal of pharmacology. 1996;314(1-2):175-83.
253. Mau SE, Witt MR, Saermark T, Vilhardt H. Substance P increases intracellular Ca2+ in individual rat pituitary lactotrophs, somatotrophs, and gonadotrophs. Molecular and cellular endocrinology. 1997;126(2):193-201.
254. Chu PJ, Best PM. Molecular cloning of calcium channel alpha(2)delta-subunits from rat atria and the differential regulation of their expression by IGF-1. Journal of molecular and cellular cardiology. 2003;35(2):207-15.
255. Kahle JJ, Gulbahce N, Shaw CA, Lim J, Hill DE, Barabasi AL, et al. Comparison of an expanded ataxia interactome with patient medical records reveals a relationship between macular degeneration and ataxia. Human molecular genetics. 2011;20(3):510-27.
256. Zhang BX, Ma X, Yeh CK, Lifschitz MD, Zhu MX, Katz MS. Epidermal growth factor-induced depletion of the intracellular Ca2+ store fails to activate capacitative Ca2+ entry in a human salivary cell line. The Journal of biological chemistry. 2002;277(50):48165-71.
257. Bandyopadhyay BC, Swaim WD, Liu X, Redman RS, Patterson RL, Ambudkar IS. Apical localization of a functional TRPC3/TRPC6-Ca2+-signaling complex in polarized epithelial cells. Role in apical Ca2+ influx. The Journal of biological chemistry. 2005;280(13):12908-16.
258. Carrillo C, Hichami A, Andreoletti P, Cherkaoui-Malki M, del Mar Cavia M, Abdoul-Azize S, et al. Diacylglycerol-containing oleic acid induces increases in [Ca(2+)](i) via TRPC3/6 channels in human T-cells. Biochimica et biophysica acta. 2012;1821(4):618-26.

259. Li XF, Kiedrowski L, Tremblay F, Fernandez FR, Perizzolo M, Winkfein RJ, et al. Importance of K+-dependent Na+/Ca2+-exchanger 2, NCKX2, in motor learning and memory. The Journal of biological chemistry. 2006;281(10):6273-82.
260. Ghavami A, Baruscotti M, Robinson RB, Hen R. Adenovirus-mediated expression of 5-HT1B receptors in cardiac ventricle myocytes; coupling to inwardly rectifying K+ channels. European journal of pharmacology. 1997;340(2-3):259-66.
261. Wahr PA, Michele DE, Metzger JM. Parvalbumin gene transfer corrects diastolic dysfunction in diseased cardiac myocytes. Proceedings of the National Academy of Sciences of the United States of America. 1999;96(21):11982-5.
262. Belge H, Gailly P, Schwaller B, Loffing J, Debaix H, Riveira-Munoz E, et al. Renal expression of parvalbumin is critical for NaCl handling and response to diuretics. Proceedings of the National Academy of Sciences of the United States of America. 2007;104(37):14849-54.
263. Waltereit R, Dammermann B, Wulff P, Scafidi J, Staubli U, Kauselmann G, et al. Arg3.1/Arc mRNA induction by Ca2+ and cAMP requires protein kinase A and mitogen-activated protein kinase/extracellular regulated kinase activation. The Journal of neuroscience : the official journal of the Society for Neuroscience. 2001;21(15):5484-93.
264. Martinez LA, Chen Y, Fischer SM, Conti CJ. Coordinated changes in cell cycle machinery occur during keratinocyte terminal differentiation. Oncogene. 1999;18(2):397-406.
265. See V, Rajala NK, Spiller DG, White MR. Calcium-dependent regulation of the cell cycle via a novel MAPK--NF-kappaB pathway in Swiss 3T3 cells. The Journal of cell biology. 2004;166(5):661-72.
266. Maccarrone M, Di Rienzo M, Battista N, Gasperi V, Guerrieri P, Rossi A, et al. The endocannabinoid system in human keratinocytes. Evidence that anandamide inhibits epidermal differentiation through CB1 receptor-dependent inhibition of protein kinase C, activation protein-1, and transglutaminase. The Journal of biological chemistry. 2003;278(36):33896-903.
267. Nishi A, Snyder GL, Greengard P. Bidirectional regulation of DARPP-32 phosphorylation by dopamine. The Journal of neuroscience : the official journal of the Society for Neuroscience. 1997;17(21):8147-55.
268. Impey S, Obrietan K, Wong ST, Poser S, Yano S, Wayman G, et al. Cross talk between ERK and PKA is required for Ca2+ stimulation of CREB-dependent transcription and ERK nuclear translocation. Neuron. 1998;21(4):869-83.
269. Veeranna, Kaji T, Boland B, Odrljin T, Mohan P, Basavarajappa BS, et al. Calpain mediates calcium-induced activation of the erk1,2 MAPK pathway and cytoskeletal phosphorylation in neurons: relevance to Alzheimer's disease. The American journal of pathology. 2004;165(3):795-805.
270. Dawson TM, Steiner JP, Dawson VL, Dinerman JL, Uhl GR, Snyder SH. Immunosuppressant FK506 enhances phosphorylation of nitric oxide synthase and protects against glutamate neurotoxicity. Proceedings of the National Academy of Sciences of the United States of America. 1993;90(21):9808-12.
271. Michel JB, Feron O, Sacks D, Michel T. Reciprocal regulation of endothelial nitric-oxide synthase by Ca2+-calmodulin and caveolin. The Journal of biological chemistry. 1997;272(25):15583-6.
272. Onions J, Hermann S, Grundstrom T. A novel type of calmodulin interaction in the inhibition of basic helix-loop-helix transcription factors. Biochemistry. 2000;39(15):4366-74.
273. Chakrabarty S, Wang H, Canaff L, Hendy GN, Appelman H, Varani J. Calcium sensing receptor in human colon carcinoma: interaction with Ca(2+) and 1,25-dihydroxyvitamin D(3). Cancer research. 2005;65(2):493-8.
274. Chodaparambil JV, Pate KT, Hepler MR, Tsai BP, Muthurajan UM, Luger K, et al. Molecular functions of the TLE tetramerization domain in Wnt target gene repression. The EMBO journal. 2014;33(7):719-31.
275. Corominas R, Yang X, Lin GN, Kang S, Shen Y, Ghamsari L, et al. Protein interaction network of alternatively spliced isoforms from brain links genetic risk factors for autism. Nature communications. 2014;5:3650.
276. Tang J, Lin Y, Zhang Z, Tikunova S, Birnbaumer L, Zhu MX. Identification of common binding sites for calmodulin and inositol 1,4,5-trisphosphate receptors on the carboxyl termini of trp channels. The Journal of biological chemistry. 2001;276(24):21303-10.
277. Taules M, Rius E, Talaya D, Lopez-Girona A, Bachs O, Agell N. Calmodulin is essential for cyclin-dependent kinase 4 (Cdk4) activity and nuclear accumulation of cyclin D1-Cdk4 during G1. The Journal of biological chemistry. 1998;273(50):33279-86.
278. Castets F, Rakitina T, Gaillard S, Moqrich A, Mattei MG, Monneron A. Zinedin, SG2NA, and striatin are calmodulin-binding, WD repeat proteins principally expressed in the brain. The Journal of biological chemistry. 2000;275(26):19970-7.

279. Perreault-Micale C, Shushan AD, Coluccio LM. Truncation of a mammalian myosin I results in loss of Ca2+-sensitive motility. The Journal of biological chemistry. 2000;275(28):21618-23.
280. Geeves MA, Perreault-Micale C, Coluccio LM. Kinetic analyses of a truncated mammalian myosin I suggest a novel isomerization event preceding nucleotide binding. The Journal of biological chemistry. 2000;275(28):21624-30.
281. Kawai T, Nomura F, Hoshino K, Copeland NG, Gilbert DJ, Jenkins NA, et al. Death-associated protein kinase 2 is a new calcium/calmodulin-dependent protein kinase that signals apoptosis through its catalytic activity. Oncogene. 1999;18(23):3471-80.
282. Plun-Favreau H, Klupsch K, Moisoi N, Gandhi S, Kjaer S, Frith D, et al. The mitochondrial protease HtrA2 is regulated by Parkinson's disease-associated kinase PINK1. Nature cell biology. 2007;9(11):1243-52.
283. Rakhilin SV, Olson PA, Nishi A, Starkova NN, Fienberg AA, Nairn AC, et al. A network of control mediated by regulator of calcium/calmodulin-dependent signaling. Science (New York, NY). 2004;306(5696):698-701.
284. Grand RJ, Perry SV. The binding of calmodulin to myelin basic protein and histone H2B. The Biochemical journal. 1980;189(2):227-40.
285. Chan KF, Robb ND, Chen WH. Myelin basic protein: interaction with calmodulin and gangliosides. Journal of neuroscience research. 1990;25(4):535-44.
286. VonDran MW, Singh H, Honeywell JZ, Dreyfus CF. Levels of BDNF impact oligodendrocyte lineage cells following a cuprizone lesion. The Journal of neuroscience : the official journal of the Society for Neuroscience. 2011;31(40):14182-90.
287. Takemoto-Kimura S, Terai H, Takamoto M, Ohmae S, Kikumura S, Segi E, et al. Molecular cloning and characterization of CLICK-III/CaMKIgamma, a novel membrane-anchored neuronal Ca2+/calmodulin-dependent protein kinase (CaMK). The Journal of biological chemistry. 2003;278(20):18597-605.
288. Pavon N, Martin AB, Mendialdua A, Moratalla R. ERK phosphorylation and FosB expression are associated with L-DOPA-induced dyskinesia in hemiparkinsonian mice. Biological psychiatry. 2006;59(1):64-74.
289. Westin JE, Vercammen L, Strome EM, Konradi C, Cenci MA. Spatiotemporal pattern of striatal ERK1/2 phosphorylation in a rat model of L-DOPA-induced dyskinesia and the role of dopamine D1 receptors. Biological psychiatry. 2007;62(7):800-10.
290. Gerfen CR, Paletzki R, Worley P. Differences between dorsal and ventral striatum in Drd1a dopamine receptor coupling of dopamine- and cAMP-regulated phosphoprotein-32 to activation of extracellular signal-regulated kinase. The Journal of neuroscience : the official journal of the Society for Neuroscience. 2008;28(28):7113-20.
291. Hong J, Katsha A, Lu P, Shyr Y, Belkhiri A, El-Rifai W. Regulation of ERBB2 receptor by t-DARPP mediates trastuzumab resistance in human esophageal adenocarcinoma. Cancer research. 2012;72(17):4504-14.
292. Vangamudi B, Zhu S, Soutto M, Belkhiri A, El-Rifai W. Regulation of beta-catenin by t-DARPP in upper gastrointestinal cancer cells. Molecular cancer. 2011;10:32.
293. Arita Y, Kihara S, Ouchi N, Maeda K, Kuriyama H, Okamoto Y, et al. Adipocyte-derived plasma protein adiponectin acts as a platelet-derived growth factor-BB-binding protein and regulates growth factor-induced common postreceptor signal in vascular smooth muscle cell. Circulation. 2002;105(24):2893-8.
294. Narita K, Chien J, Mullany SA, Staub J, Qian X, Lingle WL, et al. Loss of HSulf-1 expression enhances autocrine signaling mediated by amphiregulin in breast cancer. The Journal of biological chemistry. 2007;282(19):14413-20.
295. Hyder A, Ehnert S, Hinz H, Nussler AK, Fandrich F, Ungefroren H. EGF and HB-EGF enhance the proliferation of programmable cells of monocytic origin (PCMO) through activation of MEK/ERK signaling and improve differentiation of PCMO-derived hepatocyte-like cells. Cell communication and signaling : CCS. 2012;10(1):23.
296. Farkas LM, Krieglstein K. Heparin-binding epidermal growth factor-like growth factor (HB-EGF) regulates survival of midbrain dopaminergic neurons. Journal of neural transmission (Vienna, Austria : 1996). 2002;109(3):267-77.
297. Kim KA, Kim JH, Wang Y, Sul HS. Pref-1 (preadipocyte factor 1) activates the MEK/extracellular signal-regulated kinase pathway to inhibit adipocyte differentiation. Molecular and cellular biology. 2007;27(6):2294-308.
298. Wang Y, Zhao L, Smas C, Sul HS. Pref-1 interacts with fibronectin to inhibit adipocyte differentiation. Molecular and cellular biology. 2010;30(14):3480-92.

299. Romano D, Magalon K, Pertuit M, Rasolonjanahary R, Barlier A, Enjalbert A, et al. Conditional overexpression of the wild-type Gs alpha as the gsp oncogene initiates chronic extracellularly regulated kinase 1/2 activation and hormone hypersecretion in pituitary cell lines. Endocrinology. 2007;148(6):2973-83.
300. Pertuit M, Romano D, Zeiller C, Barlier A, Enjalbert A, Gerard C. The gsp oncogene disrupts Ras/ERK-dependent prolactin gene regulation in gsp inducible somatotroph cell line. Endocrinology. 2011;152(4):1234-43.
301. Garcia-Murillas I, Sharpe R, Pearson A, Campbell J, Natrajan R, Ashworth A, et al. An siRNA screen identifies the GNAS locus as a driver in 20q amplified breast cancer. Oncogene. 2014;33(19):2478-86.
302. Regier DS, Higbee J, Lund KM, Sakane F, Prescott SM, Topham MK. Diacylglycerol kinase iota regulates Ras guanyl-releasing protein 3 and inhibits Rap1 signaling. Proceedings of the National Academy of Sciences of the United States of America. 2005;102(21):7595-600.
303. Hsu EH, Lochan AC, Cowen DS. Activation of Akt1 by human 5-hydroxytryptamine (serotonin)1B receptors is sensitive to inhibitors of MEK. The Journal of pharmacology and experimental therapeutics. 2001;298(2):825-32.
304. Canals M, Milligan G. Constitutive activity of the cannabinoid CB1 receptor regulates the function of co-expressed Mu opioid receptors. The Journal of biological chemistry. 2008;283(17):11424-34.
305. Asimaki O, Mangoura D. Cannabinoid receptor 1 induces a biphasic ERK activation via multiprotein signaling complex formation of proximal kinases PKCepsilon, Src, and Fyn in primary neurons. Neurochemistry international. 2011;58(2):135-44.
306. Pan B, Zhong P, Sun D, Liu QS. Extracellular signal-regulated kinase signaling in the ventral tegmental area mediates cocaine-induced synaptic plasticity and rewarding effects. The Journal of neuroscience : the official journal of the Society for Neuroscience. 2011;31(31):11244-55.
307. Subbanna S, Shivakumar M, Psychoyos D, Xie S, Basavarajappa BS. Anandamide-CB1 receptor signaling contributes to postnatal ethanol-induced neonatal neurodegeneration, adult synaptic, and memory deficits. The Journal of neuroscience : the official journal of the Society for Neuroscience. 2013;33(15):6350-66.
308. Kehat I, Molkentin JD. Extracellular signal-regulated kinase 1/2 (ERK1/2) signaling in cardiac hypertrophy. Annals of the New York Academy of Sciences. 2010;1188:96-102.
309. Mori Sequeiros Garcia M, Gomez NV, Gorostizaga A, Acquier A, Gonzalez-Calvar SI, Mendez CF, et al. MAP kinase phosphatase-3 (MKP-3) is transcriptionally and post-translationally up-regulated by hCG and modulates cAMP-induced p21 expression in MA-10 Leydig cells. Molecular and cellular endocrinology. 2013;371(1-2):174-81.
310. Zhang Z, Mitra RS, Henson BS, Datta NS, McCauley LK, Kumar P, et al. Rap1GAP inhibits tumor growth in oropharyngeal squamous cell carcinoma. The American journal of pathology. 2006;168(2):585-96.
311. Fan HY, Liu Z, Johnson PF, Richards JS. CCAAT/enhancer-binding proteins (C/EBP)-alpha and -beta are essential for ovulation, luteinization, and the expression of key target genes. Molecular endocrinology (Baltimore, Md). 2011;25(2):253-68.
312. Trumper A, Trumper K, Trusheim H, Arnold R, Goke B, Horsch D. Glucose-dependent insulinotropic polypeptide is a growth factor for beta (INS-1) cells by pleiotropic signaling. Molecular endocrinology (Baltimore, Md). 2001;15(9):1559-70.
313. Cui H, Darmanin S, Natsuisaka M, Kondo T, Asaka M, Shindoh M, et al. Enhanced expression of asparagine synthetase under glucose-deprived conditions protects pancreatic cancer cells from apoptosis induced by glucose deprivation and cisplatin. Cancer research. 2007;67(7):3345-55.
314. See V, Boutillier AL, Bito H, Loeffler JP. Calcium/calmodulin-dependent protein kinase type IV (CaMKIV) inhibits apoptosis induced by potassium deprivation in cerebellar granule neurons. FASEB journal : official publication of the Federation of American Societies for Experimental Biology. 2001;15(1):134-44.
315. Du K, Montminy M. CREB is a regulatory target for the protein kinase Akt/PKB. The Journal of biological chemistry. 1998;273(49):32377-9.
316. Yang X, Lee FY, Sr., Wand GS. Increased expression of Gs(alpha) enhances activation of the adenylyl cyclase signal transduction cascade. Molecular endocrinology (Baltimore, Md). 1997;11(8):1053-61.
317. Fox KE, Fankell DM, Erickson PF, Majka SM, Crossno JT, Jr., Klemm DJ. Depletion of cAMP-response element-binding protein/ATF1 inhibits adipogenic conversion of 3T3-L1 cells ectopically expressing CCAAT/enhancer-binding protein (C/EBP) alpha, C/EBP beta, or PPAR gamma 2. The Journal of biological chemistry. 2006;281(52):40341-53.

318. Li D, Yea S, Li S, Chen Z, Narla G, Banck M, et al. Kruppel-like factor-6 promotes preadipocyte differentiation through histone deacetylase 3-dependent repression of DLK1. The Journal of biological chemistry. 2005;280(29):26941-52.
319. Barco A, Alarcon JM, Kandel ER. Expression of constitutively active CREB protein facilitates the late phase of long-term potentiation by enhancing synaptic capture. Cell. 2002;108(5):689-703.
320. Tsuchida T, Parker KC, Turner RV, McFarland HF, Coligan JE, Biddison WE. Autoreactive CD8+ T-cell responses to human myelin protein-derived peptides. Proceedings of the National Academy of Sciences of the United States of America. 1994;91(23):10859-63.
321. Ferreira P, Meyer I, Mollner S, Frank R, Pfeuffer T. Selective formation of Gsalpha-MHC I complexes after desensitization of human platelets with iloprost. European journal of biochemistry / FEBS. 1999;259(1-2):167-74.
322. Mimura K, Shiraishi K, Mueller A, Izawa S, Kua LF, So J, et al. The MAPK pathway is a predominant regulator of HLA-A expression in esophageal and gastric cancer. Journal of immunology (Baltimore, Md : 1950). 2013;191(12):6261-72.
323. Landles C, Bates GP. Huntingtin and the molecular pathogenesis of Huntington's disease. Fourth in molecular medicine review series. EMBO reports. 2004;5(10):958-63.
324. Carlezon WA, Jr., Thome J, Olson VG, Lane-Ladd SB, Brodkin ES, Hiroi N, et al. Regulation of cocaine reward by CREB. Science (New York, NY). 1998;282(5397):2272-5.
325. Schiffmann SN, Fisone G, Moresco R, Cunha RA, Ferre S. Adenosine A2A receptors and basal ganglia physiology. Progress in neurobiology. 2007;83(5):277-92.
326. Lee RJ, Albanese C, Stenger RJ, Watanabe G, Inghirami G, Haines GK, 3rd, et al. pp60(v-src) induction of cyclin D1 requires collaborative interactions between the extracellular signal-regulated kinase, p38, and Jun kinase pathways. A role for cAMP response element-binding protein and activating transcription factor-2 in pp60(v-src) signaling in breast cancer cells. The Journal of biological chemistry. 1999;274(11):7341-50.
327. Welsh CF, Roovers K, Villanueva J, Liu Y, Schwartz MA, Assoian RK. Timing of cyclin D1 expression within G1 phase is controlled by Rho. Nature cell biology. 2001;3(11):950-7.
328. Calipel A, Lefevre G, Pouponnot C, Mouriaux F, Eychene A, Mascarelli F. Mutation of B-Raf in human choroidal melanoma cells mediates cell proliferation and transformation through the MEK/ERK pathway. The Journal of biological chemistry. 2003;278(43):42409-18.
329. Wu K, Li S, Bodhinathan K, Meyers C, Chen W, Campbell-Thompson M, et al. Enhanced expression of Pctk1, Tcf12 and Ccnd1 in hippocampus of rats: Impact on cognitive function, synaptic plasticity and pathology. Neurobiology of learning and memory. 2012;97(1):69-80.
330. Yang ZF, Ho DW, Lam CT, Luk JM, Lum CT, Yu WC, et al. Identification of brain-derived neurotrophic factor as a novel functional protein in hepatocellular carcinoma. Cancer research. 2005;65(1):219-25.
331. Yeo EJ, Cassetta L, Qian BZ, Lewkowich I, Li JF, Stefater JA, 3rd, et al. Myeloid WNT7b mediates the angiogenic switch and metastasis in breast cancer. Cancer research. 2014;74(11):2962-73.
332. Gille H, Downward J. Multiple ras effector pathways contribute to G(1) cell cycle progression. The Journal of biological chemistry. 1999;274(31):22033-40.
333. Ackler S, Ahmad S, Tobias C, Johnson MD, Glazer RI. Delayed mammary gland involution in MMTV-AKT1 transgenic mice. Oncogene. 2002;21(2):198-206.
334. Grabinski N, Bartkowiak K, Grupp K, Brandt B, Pantel K, Jucker M. Distinct functional roles of Akt isoforms for proliferation, survival, migration and EGF-mediated signalling in lung cancer derived disseminated tumor cells. Cellular signalling. 2011;23(12):1952-60.
335. Hamanaka RB, Bennett BS, Cullinan SB, Diehl JA. PERK and GCN2 contribute to eIF2alpha phosphorylation and cell cycle arrest after activation of the unfolded protein response pathway. Molecular biology of the cell. 2005;16(12):5493-501.
336. Quasnichka H, Slater SC, Beeching CA, Boehm M, Sala-Newby GB, George SJ. Regulation of smooth muscle cell proliferation by beta-catenin/T-cell factor signaling involves modulation of cyclin D1 and p21 expression. Circulation research. 2006;99(12):1329-37.
337. Bedel A, Negre-Salvayre A, Heeneman S, Grazide MH, Thiers JC, Salvayre R, et al. E-cadherin/beta-catenin/T-cell factor pathway is involved in smooth muscle cell proliferation elicited by oxidized low-density lipoprotein. Circulation research. 2008;103(7):694-701.
338. Yeo CW, Ng FS, Chai C, Tan JM, Koh GR, Chong YK, et al. Parkin pathway activation mitigates glioma cell proliferation and predicts patient survival. Cancer research. 2012;72(10):2543-53.

339. Mejlvang J, Kriajevska M, Vandewalle C, Chernova T, Sayan AE, Berx G, et al. Direct repression of cyclin D1 by SIP1 attenuates cell cycle progression in cells undergoing an epithelial mesenchymal transition. Molecular biology of the cell. 2007;18(11):4615-24.
340. Karreth FA, Tay Y, Perna D, Ala U, Tan SM, Rust AG, et al. In vivo identification of tumor- suppressive PTEN ceRNAs in an oncogenic BRAF-induced mouse model of melanoma. Cell. 2011;147(2):382-95.
341. Li Y, Dowbenko D, Lasky LA. AKT/PKB phosphorylation of p21Cip/WAF1 enhances protein stability of p21Cip/WAF1 and promotes cell survival. The Journal of biological chemistry. 2002;277(13):11352-61.
342. Benzeno S, Narla G, Allina J, Cheng GZ, Reeves HL, Banck MS, et al. Cyclin-dependent kinase inhibition by the KLF6 tumor suppressor protein through interaction with cyclin D1. Cancer research. 2004;64(11):3885-91.
343. Comstock CE, Augello MA, Schiewer MJ, Karch J, Burd CJ, Ertel A, et al. Cyclin D1 is a selective modifier of androgen-dependent signaling and androgen receptor function. The Journal of biological chemistry. 2011;286(10):8117-27.
344. Ju X, Casimiro MC, Gormley M, Meng H, Jiao X, Katiyar S, et al. Identification of a cyclin D1 network in prostate cancer that antagonizes epithelial-mesenchymal restraint. Cancer research. 2014;74(2):508-19.
345. Jirawatnotai S, Hu Y, Michowski W, Elias JE, Becks L, Bienvenu F, et al. A function for cyclin D1 in DNA repair uncovered by protein interactome analyses in human cancers. Nature. 2011;474(7350):230-4.
346. Lee MY, Luciano AK, Ackah E, Rodriguez-Vita J, Bancroft TA, Eichmann A, et al. Endothelial Akt1 mediates angiogenesis by phosphorylating multiple angiogenic substrates. Proceedings of the National Academy of Sciences of the United States of America. 2014;111(35):12865-70.
347. Casanovas O, Miro F, Estanyol JM, Itarte E, Agell N, Bachs O. Osmotic stress regulates the stability of cyclin D1 in a p38SAPK2-dependent manner. The Journal of biological chemistry. 2000;275(45):35091-7.
348. Bisogno T, Di Marzo V. Cannabinoid receptors and endocannabinoids: role in neuroinflammatory and neurodegenerative disorders. CNS & neurological disorders drug targets. 2010;9(5):564-73.
349. Pisani V, Madeo G, Tassone A, Sciamanna G, Maccarrone M, Stanzione P, et al. Homeostatic changes of the endocannabinoid system in Parkinson's disease. Movement disorders : official journal of the Movement Disorder Society. 2011;26(2):216-22.
350. Pacher P, Batkai S, Kunos G. The endocannabinoid system as an emerging target of pharmacotherapy. Pharmacological reviews. 2006;58(3):389-462.
351. Parsons LH, Hurd YL. Endocannabinoid signalling in reward and addiction. Nature reviews Neuroscience. 2015;16(10):579-94.
352. Howlett AC, Barth F, Bonner TI, Cabral G, Casellas P, Devane WA, et al. International Union of Pharmacology. XXVII. Classification of cannabinoid receptors. Pharmacological reviews. 2002;54(2):161-202.
353. Meschler JP, Howlett AC. Signal transduction interactions between CB1 cannabinoid and dopamine receptors in the rat and monkey striatum. Neuropharmacology. 2001;40(7):918-26.
354. Garcia C, Palomo-Garo C, Gomez-Galvez Y, Fernandez-Ruiz J. Cannabinoid-dopamine interactions in the physiology and physiopathology of the basal ganglia. British journal of pharmacology. 2015.
355. Compagnucci C, Di Siena S, Bustamante MB, Di Giacomo D, Di Tommaso M, Maccarrone M, et al. Type-1 (CB1) cannabinoid receptor promotes neuronal differentiation and maturation of neural stem cells. PloS one. 2013;8(1):e54271.
356. Kreitzer AC, Malenka RC. Endocannabinoid-mediated rescue of striatal LTD and motor deficits in Parkinson's disease models. Nature. 2007;445(7128):643-7.
357. Di Filippo M, Picconi B, Tozzi A, Ghiglieri V, Rossi A, Calabresi P. The endocannabinoid system in Parkinson's disease. Current pharmaceutical design. 2008;14(23):2337-47.
358. Huot P, Johnston TH, Koprich JB, Fox SH, Brotchie JM. The pharmacology of L-DOPA-induced dyskinesia in Parkinson's disease. Pharmacological reviews. 2013;65(1):171-222.
359. Jarrahian A, Watts VJ, Barker EL. D2 dopamine receptors modulate Galpha-subunit coupling of the CB1 cannabinoid receptor. The Journal of pharmacology and experimental therapeutics. 2004;308(3):880-6.
360. Meurers BH, Dziewczapolski G, Shi T, Bittner A, Kamme F, Shults CW. Dopamine depletion induces distinct compensatory gene expression changes in DARPP-32 signal transduction cascades of striatonigral and striatopallidal neurons. The Journal of neuroscience : the official journal of the Society for Neuroscience. 2009;29(21):6828-39.

361. Nam DH, Lee MH, Kim JE, Song HK, Kang YS, Lee JE, et al. Blockade of cannabinoid receptor 1 improves insulin resistance, lipid metabolism, and diabetic nephropathy in db/db mice. Endocrinology. 2012;153(3):1387-96.
362. Maison P, Walker DJ, Walsh FS, Williams G, Doherty P. BDNF regulates neuronal sensitivity to endocannabinoids. Neuroscience letters. 2009;467(2):90-4.
363. Aguado T, Romero E, Monory K, Palazuelos J, Sendtner M, Marsicano G, et al. The CB1 cannabinoid receptor mediates excitotoxicity-induced neural progenitor proliferation and neurogenesis. The Journal of biological chemistry. 2007;282(33):23892-8.
364. Jordan JD, He JC, Eungdamrong NJ, Gomes I, Ali W, Nguyen T, et al. Cannabinoid receptor-induced neurite outgrowth is mediated by Rap1 activation through G(alpha)o/i-triggered proteasomal degradation of Rap1GAPII. The Journal of biological chemistry. 2005;280(12):11413-21.
365. Nie J, Lewis DL. The proximal and distal C-terminal tail domains of the CB1 cannabinoid receptor mediate G protein coupling. Neuroscience. 2001;107(1):161-7.
366. Maccarrone M, Battista N, Centonze D. The endocannabinoid pathway in Huntington's disease: a comparison with other neurodegenerative diseases. Progress in neurobiology. 2007;81(5-6):349-79.
367. Steiner H, Bonner TI, Zimmer AM, Kitai ST, Zimmer A. Altered gene expression in striatal projection neurons in CB1 cannabinoid receptor knockout mice. Proceedings of the National Academy of Sciences of the United States of America. 1999;96(10):5786-90.
368. Roche JP, Bounds S, Brown S, Mackie K. A mutation in the second transmembrane region of the CB1 receptor selectively disrupts G protein signaling and prevents receptor internalization. Molecular pharmacology. 1999;56(3):611-8.
369. Ahn KH, Mahmoud MM, Kendall DA. Allosteric modulator ORG27569 induces CB1 cannabinoid receptor high affinity agonist binding state, receptor internalization, and Gi protein-independent ERK1/2 kinase activation. The Journal of biological chemistry. 2012;287(15):12070-82.
370. van der Stelt M, Di Marzo V. The endocannabinoid system in the basal ganglia and in the mesolimbic reward system: implications for neurological and psychiatric disorders. European journal of pharmacology. 2003;480(1-3):133-50.
371. Loewinger GC, Beckert MV, Tejeda HA, Cheer JF. Methamphetamine-induced dopamine terminal deficits in the nucleus accumbens are exacerbated by reward-associated cues and attenuated by CB1 receptor antagonism. Neuropharmacology. 2012;62(7):2192-201.
372. Gebeh AK, Willets JM, Marczylo EL, Taylor AH, Konje JC. Ectopic pregnancy is associated with high anandamide levels and aberrant expression of FAAH and CB1 in fallopian tubes. The Journal of clinical endocrinology and metabolism. 2012;97(8):2827-35.
373. Blankman JL, Cravatt BF. Chemical probes of endocannabinoid metabolism. Pharmacological reviews. 2013;65(2):849-71.
374. Aaltonen N, Riera Ribas C, Lehtonen M, Savinainen JR, Laitinen JT. Brain regional cannabinoid CB(1) receptor signalling and alternative enzymatic pathways for 2-arachidonoylglycerol generation in brain sections of diacylglycerol lipase deficient mice. European journal of pharmaceutical sciences : official journal of the European Federation for Pharmaceutical Sciences. 2014;51:87-95.
375. Patsenker E, Stoll M, Millonig G, Agaimy A, Wissniowski T, Schneider V, et al. Cannabinoid receptor type I modulates alcohol-induced liver fibrosis. Molecular medicine (Cambridge, Mass). 2011;17(11-12):1285-94.
376. Maccarrone M, Salvati S, Bari M, Finazzi A. Anandamide and 2-arachidonoylglycerol inhibit fatty acid amide hydrolase by activating the lipoxygenase pathway of the arachidonate cascade. Biochemical and biophysical research communications. 2000;278(3):576-83.
377. Fowler CJ, Janson U, Johnson RM, Wahlstrom G, Stenstrom A, Norstrom K, et al. Inhibition of anandamide hydrolysis by the enantiomers of ibuprofen, ketorolac, and flurbiprofen. Archives of biochemistry and biophysics. 1999;362(2):191-6.
378. Robinson RT, Drafts BC, Fisher JL. Fluoxetine increases GABA(A) receptor activity through a novel modulatory site. The Journal of pharmacology and experimental therapeutics. 2003;304(3):978-84.
379. Sigel E, Baur R, Racz I, Marazzi J, Smart TG, Zimmer A, et al. The major central endocannabinoid directly acts at GABA(A) receptors. Proceedings of the National Academy of Sciences of the United States of America. 2011;108(44):18150-5.
380. Henry B, Duty S, Fox SH, Crossman AR, Brotchie JM. Increased striatal pre-proenkephalin B expression is associated with dyskinesia in Parkinson's disease. Experimental neurology. 2003;183(2):458-68.

381. Hanrieder J, Ljungdahl A, Falth M, Mammo SE, Bergquist J, Andersson M. L-DOPA-induced dyskinesia is associated with regional increase of striatal dynorphin peptides as elucidated by imaging mass spectrometry. Molecular & cellular proteomics : MCP. 2011;10(10):M111.009308.
382. Bourdenx M, Nilsson A, Wadensten H, Falth M, Li Q, Crossman AR, et al. Abnormal structure-specific peptide transmission and processing in a primate model of Parkinson's disease and l-DOPA-induced dyskinesia. Neurobiology of disease. 2014;62:307-12.
383. Bernardis LL, Bellinger LL. The lateral hypothalamic area revisited: ingestive behavior. Neuroscience and biobehavioral reviews. 1996;20(2):189-287.
384. Waschek JA, Dave JR, Eskay RL, Eiden LE. Barium distinguishes separate calcium targets for synthesis and secretion of peptides in neuroendocrine cells. Biochemical and biophysical research communications. 1987;146(2):495-501.
385. Salin P, Manrique C, Forni C, Kerkerian-Le Goff L. High-frequency stimulation of the subthalamic nucleus selectively reverses dopamine denervation-induced cellular defects in the output structures of the basal ganglia in the rat. The Journal of neuroscience : the official journal of the Society for Neuroscience. 2002;22(12):5137-48.
386. Xu K, Bastia E, Schwarzschild M. Therapeutic potential of adenosine A(2A) receptor antagonists in Parkinson's disease. Pharmacology & therapeutics. 2005;105(3):267-310.
387. Zuccato C, Cattaneo E. Role of brain-derived neurotrophic factor in Huntington's disease. Progress in neurobiology. 2007;81(5-6):294-330.
388. Aoyama S, Kase H, Borrelli E. Rescue of locomotor impairment in dopamine D2 receptor-deficient mice by an adenosine A2A receptor antagonist. The Journal of neuroscience : the official journal of the Society for Neuroscience. 2000;20(15):5848-52.
389. Liu B, Qin L, Yang SN, Wilson BC, Liu Y, Hong JS. Femtomolar concentrations of dynorphins protect rat mesencephalic dopaminergic neurons against inflammatory damage. The Journal of pharmacology and experimental therapeutics. 2001;298(3):1133-41.
390. Josefsen K, Buschard K, Sorensen LR, Wollike M, Ekman R, Birkenbach M. Glucose stimulation of pancreatic beta-cell lines induces expression and secretion of dynorphin. Endocrinology. 1998;139(10):4329-36.
391. Moratalla R, Xu M, Tonegawa S, Graybiel AM. Cellular responses to psychomotor stimulant and neuroleptic drugs are abnormal in mice lacking the D1 dopamine receptor. Proceedings of the National Academy of Sciences of the United States of America. 1996;93(25):14928-33.
392. Saban MR, Nguyen NB, Hammond TG, Saban R. Gene expression profiling of mouse bladder inflammatory responses to LPS, substance P, and antigen-stimulation. The American journal of pathology. 2002;160(6):2095-110.
393. Arenas E, Akerud P, Wong V, Boylan C, Persson H, Lindsay RM, et al. Effects of BDNF and NT-4/5 on striatonigral neuropeptides or nigral GABA neurons in vivo. The European journal of neuroscience. 1996;8(8):1707-17.
394. Perez-Navarro E, Alberch J, Neveu I, Arenas E. Brain-derived neurotrophic factor, neurotrophin-3 and neurotrophin-4/5 differentially regulate the phenotype and prevent degenerative changes in striatal projection neurons after excitotoxicity in vivo. Neuroscience. 1999;91(4):1257-64.
395. Koon HW, Zhao D, Na X, Moyer MP, Pothoulakis C. Metalloproteinases and transforming growth factor-alpha mediate substance P-induced mitogen-activated protein kinase activation and proliferation in human colonocytes. The Journal of biological chemistry. 2004;279(44):45519-27.
396. Backman LJ, Fong G, Andersson G, Scott A, Danielson P. Substance P is a mechanoresponsive, autocrine regulator of human tenocyte proliferation. PloS one. 2011;6(11):e27209.
397. Yoshino H, Wakita M, Izumi Y. Calcium-dependent changes in structure of calmodulin with substance P. The Journal of biological chemistry. 1993;268(16):12123-8.
398. Guo W, Wang H, Watanabe M, Shimizu K, Zou S, LaGraize SC, et al. Glial-cytokine-neuronal interactions underlying the mechanisms of persistent pain. The Journal of neuroscience : the official journal of the Society for Neuroscience. 2007;27(22):6006-18.
399. Zhang X, Wang J, Zhou Q, Xu Y, Pu S, Wu J, et al. Brain-derived neurotrophic factor-activated astrocytes produce mechanical allodynia in neuropathic pain. Neuroscience. 2011;199:452-60.
400. Kaminski MM, Sauer SW, Kaminski M, Opp S, Ruppert T, Grigaravicius P, et al. T cell activation is driven by an ADP-dependent glucokinase linking enhanced glycolysis with mitochondrial reactive oxygen species generation. Cell reports. 2012;2(5):1300-15.
401. Gong Q, Brown LJ, MacDonald MJ. Functional analysis of two promoters for the human mitochondrial glycerol phosphate dehydrogenase gene. The Journal of biological chemistry. 2000;275(48):38012-21.

402. Wishart DS, Knox C, Guo AC, Shrivastava S, Hassanali M, Stothard P, et al. DrugBank: a comprehensive resource for in silico drug discovery and exploration. Nucleic acids research. 2006;34(Database issue):D668-72.
403. Pinna A, Bonaventura J, Farre D, Sanchez M, Simola N, Mallol J, et al. L-DOPA disrupts adenosine A(2A)-cannabinoid CB(1)-dopamine D(2) receptor heteromer cross-talk in the striatum of hemiparkinsonian rats: biochemical and behavioral studies. Experimental neurology. 2014;253:180-91.
404. Martinez A, Macheda T, Morgese MG, Trabace L, Giuffrida A. The cannabinoid agonist WIN55212-2 decreases L-DOPA-induced PKA activation and dyskinetic behavior in 6-OHDA-treated rats. Neuroscience research. 2012;72(3):236-42.
405. Song L, Yang X, Ma Y, Wu N, Liu Z. The CB1 cannabinoid receptor agonist reduces L-DOPA-induced motor fluctuation and ERK1/2 phosphorylation in 6-OHDA-lesioned rats. Drug design, development and therapy. 2014;8:2173-9.
406. Cenci MA, Lee CS, Bjorklund A. L-DOPA-induced dyskinesia in the rat is associated with striatal overexpression of prodynorphin- and glutamic acid decarboxylase mRNA. The European journal of neuroscience. 1998;10(8):2694-706.
407. Tamim MK, Samadi P, Morissette M, Gregoire L, Ouattara B, Levesque D, et al. Effect of non-dopaminergic drug treatment on Levodopa induced dyskinesias in MPTP monkeys: common implication of striatal neuropeptides. Neuropharmacology. 2010;58(1):286-96.
408. Morin N, Jourdain VA, Morissette M, Gregoire L, Di Paolo T. Long-term treatment with l-DOPA and an mGlu5 receptor antagonist prevents changes in brain basal ganglia dopamine receptors, their associated signaling proteins and neuropeptides in parkinsonian monkeys. Neuropharmacology. 2014;79:688-706.
409. Ding Y, Won L, Britt JP, Lim SA, McGehee DS, Kang UJ. Enhanced striatal cholinergic neuronal activity mediates L-DOPA-induced dyskinesia in parkinsonian mice. Proceedings of the National Academy of Sciences of the United States of America. 2011;108(2):840-5.
410. Won L, Ding Y, Singh P, Kang UJ. Striatal cholinergic cell ablation attenuates L-DOPA induced dyskinesia in Parkinsonian mice. The Journal of neuroscience : the official journal of the Society for Neuroscience. 2014;34(8):3090-4.
411. Herdegen T, Leah JD. Inducible and constitutive transcription factors in the mammalian nervous system: control of gene expression by Jun, Fos and Krox, and CREB/ATF proteins. Brain research Brain research reviews. 1998;28(3):370-490.
412. Mount MP, Zhang Y, Amini M, Callaghan S, Kulczycki J, Mao Z, et al. Perturbation of transcription factor Nur77 expression mediated by myocyte enhancer factor 2D (MEF2D) regulates dopaminergic neuron loss in response to 1-methyl-4-phenyl-1,2,3,6-tetrahydropyridine (MPTP). The Journal of biological chemistry. 2013;288(20):14362-71.
413. Wilhelm KR, Yanamandra K, Gruden MA, Zamotin V, Malisauskas M, Casaite V, et al. Immune reactivity towards insulin, its amyloid and protein S100B in blood sera of Parkinson's disease patients. European journal of neurology. 2007;14(3):327-34.
414. Andican G, Konukoglu D, Bozluolcay M, Bayulkem K, Firtiina S, Burcak G. Plasma oxidative and inflammatory markers in patients with idiopathic Parkinson's disease. Acta neurologica Belgica. 2012;112(2):155-9.
415. Ohlsson BG, Englund MC, Karlsson AL, Knutsen E, Erixon C, Skribeck H, et al. Oxidized low density lipoprotein inhibits lipopolysaccharide-induced binding of nuclear factor-kappaB to DNA and the subsequent expression of tumor necrosis factor-alpha and interleukin-1beta in macrophages. The Journal of clinical investigation. 1996;98(1):78-89.
416. Ryoo S, Won M, Kim DU, Kim L, Han G, Park SK, et al. PPARalpha activation abolishes LDL-stimulated IL-8 production via AP-1 deactivation in human aortic smooth muscle cells. Biochemical and biophysical research communications. 2004;318(2):329-34.
417. Mikita T, Porter G, Lawn RM, Shiffman D. Oxidized low density lipoprotein exposure alters the transcriptional response of macrophages to inflammatory stimulus. The Journal of biological chemistry. 2001;276(49):45729-39.
418. Pei L, Castrillo A, Chen M, Hoffmann A, Tontonoz P. Induction of NR4A orphan nuclear receptor expression in macrophages in response to inflammatory stimuli. The Journal of biological chemistry. 2005;280(32):29256-62.
419. Kusari AB, Byon J, Bandyopadhyay D, Kenner KA, Kusari J. Insulin-induced mitogen-activated protein (MAP) kinase phosphatase-1 (MKP-1) attenuates insulin-stimulated MAP kinase activity: a mechanism

for the feedback inhibition of insulin signaling. Molecular endocrinology (Baltimore, Md). 1997;11(10):1532-43.
420. Metzler B, Li C, Hu Y, Sturm G, Ghaffari-Tabrizi N, Xu Q. LDL stimulates mitogen-activated protein kinase phosphatase-1 expression, independent of LDL receptors, in vascular smooth muscle cells. Arteriosclerosis, thrombosis, and vascular biology. 1999;19(8):1862-71.
421. Desbois-Mouthon C, Cadoret A, Blivet-Van Eggelpoel MJ, Bertrand F, Caron M, Atfi A, et al. Insulin-mediated cell proliferation and survival involve inhibition of c-Jun N-terminal kinases through a phosphatidylinositol 3-kinase- and mitogen-activated protein kinase phosphatase-1-dependent pathway. Endocrinology. 2000;141(3):922-31.
422. Ruggenenti P, Remuzzi G. Time to abandon microalbuminuria? Kidney international. 2006;70(7):1214-22.
423. Srivastava M, Gupta SP. Protein metabolism in Isoparorchis hypselobagri. Zeitschrift fur Parasitenkunde (Berlin, Germany). 1976;49(2):179-82.
424. Perkinton MS, Sihra TS, Williams RJ. Ca(2+)-permeable AMPA receptors induce phosphorylation of cAMP response element-binding protein through a phosphatidylinositol 3-kinase-dependent stimulation of the mitogen-activated protein kinase signaling cascade in neurons. The Journal of neuroscience : the official journal of the Society for Neuroscience. 1999;19(14):5861-74.
425. Gupta S, Stuffrein S, Plattner R, Tencati M, Gray C, Whang YE, et al. Role of phosphoinositide 3-kinase in the aggressive tumor growth of HT1080 human fibrosarcoma cells. Molecular and cellular biology. 2001;21(17):5846-56.
426. Choi J, Park SY, Joo CK. Hepatocyte growth factor induces proliferation of lens epithelial cells through activation of ERK1/2 and JNK/SAPK. Investigative ophthalmology & visual science. 2004;45(8):2696-704.
427. Reddy SA, Huang JH, Liao WS. Phosphatidylinositol 3-kinase in interleukin 1 signaling. Physical interaction with the interleukin 1 receptor and requirement in NFkappaB and AP-1 activation. The Journal of biological chemistry. 1997;272(46):29167-73.
428. Chen Y, Freund R, Listerud M, Wang Z, Talmage DA. Retinoic acid inhibits transformation by preventing phosphatidylinositol 3-kinase dependent activation of the c-fos promoter. Oncogene. 1999;18(1):139-48.
429. Dentelli P, Rosso A, Zeoli A, Gambino R, Pegoraro L, Pagano G, et al. Oxidative stress-mediated mesangial cell proliferation requires RAC-1/reactive oxygen species production and beta4 integrin expression. The Journal of biological chemistry. 2007;282(36):26101-10.
430. Dominguez JE, Munoz MC, Zafra D, Sanchez-Perez I, Baque S, Caron M, et al. The antidiabetic agent sodium tungstate activates glycogen synthesis through an insulin receptor-independent pathway. The Journal of biological chemistry. 2003;278(44):42785-94.
431. Ketsawatsomkron P, Stepp DW, Fulton DJ, Marrero MB. Molecular mechanism of angiotensin II-induced insulin resistance in aortic vascular smooth muscle cells: roles of Protein Tyrosine Phosphatase-1B. Vascular pharmacology. 2010;53(3-4):160-8.
432. Wang X, Wang L, Zhu L, Pan Y, Xiao F, Liu W, et al. PAQR3 modulates insulin signaling by shunting phosphoinositide 3-kinase p110alpha to the Golgi apparatus. Diabetes. 2013;62(2):444-56.
433. Iwata A, Maruyama M, Kanazawa I, Nukina N. alpha-Synuclein affects the MAPK pathway and accelerates cell death. The Journal of biological chemistry. 2001;276(48):45320-9.
434. Lin DC, Xu L, Chen Y, Yan H, Hazawa M, Doan N, et al. Genomic and Functional Analysis of the E3 Ligase PARK2 in Glioma. Cancer research. 2015;75(9):1815-27.
435. Perkinton MS, Ip JK, Wood GL, Crossthwaite AJ, Williams RJ. Phosphatidylinositol 3-kinase is a central mediator of NMDA receptor signalling to MAP kinase (Erk1/2), Akt/PKB and CREB in striatal neurones. Journal of neurochemistry. 2002;80(2):239-54.
436. Xu J, Kurup P, Zhang Y, Goebel-Goody SM, Wu PH, Hawasli AH, et al. Extrasynaptic NMDA receptors couple preferentially to excitotoxicity via calpain-mediated cleavage of STEP. The Journal of neuroscience : the official journal of the Society for Neuroscience. 2009;29(29):9330-43.
437. Link W, Konietzko U, Kauselmann G, Krug M, Schwanke B, Frey U, et al. Somatodendritic expression of an immediate early gene is regulated by synaptic activity. Proceedings of the National Academy of Sciences of the United States of America. 1995;92(12):5734-8.
438. Sun WL, Zhou L, Hazim R, Quinones-Jenab V, Jenab S. Effects of dopamine and NMDA receptors on cocaine-induced Fos expression in the striatum of Fischer rats. Brain research. 2008;1243:1-9.

439. Chen P, Hutter D, Yang X, Gorospe M, Davis RJ, Liu Y. Discordance between the binding affinity of mitogen-activated protein kinase subfamily members for MAP kinase phosphatase-2 and their ability to activate the phosphatase catalytically. The Journal of biological chemistry. 2001;276(31):29440-9.
440. Bueno OF, De Windt LJ, Lim HW, Tymitz KM, Witt SA, Kimball TR, et al. The dual-specificity phosphatase MKP-1 limits the cardiac hypertrophic response in vitro and in vivo. Circulation research. 2001;88(1):88-96.
441. Kassel O, Sancono A, Kratzschmar J, Kreft B, Stassen M, Cato AC. Glucocorticoids inhibit MAP kinase via increased expression and decreased degradation of MKP-1. The EMBO journal. 2001;20(24):7108-16.
442. Stockand JD. New ideas about aldosterone signaling in epithelia. American journal of physiology Renal physiology. 2002;282(4):F559-76.
443. Brion L, Maloberti PM, Gomez NV, Poderoso C, Gorostizaga AB, Mori Sequeiros Garcia MM, et al. MAPK phosphatase-1 (MKP-1) expression is up-regulated by hCG/cAMP and modulates steroidogenesis in MA-10 Leydig cells. Endocrinology. 2011;152(7):2665-77.
444. Liu F, Austin DA, Mellon PL, Olefsky JM, Webster NJ. GnRH activates ERK1/2 leading to the induction of c-fos and LHbeta protein expression in LbetaT2 cells. Molecular endocrinology (Baltimore, Md). 2002;16(3):419-34.
445. Bode AM, Dong Z. Mitogen-activated protein kinase activation in UV-induced signal transduction. Science's STKE : signal transduction knowledge environment. 2003;2003(167):Re2.
446. Hong JW, Ryu MS, Lim IK. Phosphorylation of serine 147 of tis21/BTG2/pc3 by p-Erk1/2 induces Pin-1 binding in cytoplasm and cell death. The Journal of biological chemistry. 2005;280(22):21256-63.
447. Lianguzova MS, Chuikin IA, Pospelov VA. [PI 3-kinase activity is necessary for F9 mouse embryonic carcinoma cell proliferation]. Tsitologiia. 2004;46(1):26-34.
448. Yang R, Barouch LA. Leptin signaling and obesity: cardiovascular consequences. Circulation research. 2007;101(6):545-59.
449. Byun HJ, Hong IK, Kim E, Jin YJ, Jeoung DI, Hahn JH, et al. A splice variant of CD99 increases motility and MMP-9 expression of human breast cancer cells through the AKT-, ERK-, and JNK-dependent AP-1 activation signaling pathways. The Journal of biological chemistry. 2006;281(46):34833-47.
450. Chandrasekharan UM, Yang L, Walters A, Howe P, DiCorleto PE. Role of CL-100, a dual specificity phosphatase, in thrombin-induced endothelial cell activation. The Journal of biological chemistry. 2004;279(45):46678-85.
451. Jin HS, Kim J, Lee SJ, Kim K, Go MJ, Lee JY, et al. The PARK2 gene is involved in the maintenance of pancreatic beta-cell functions related to insulin production and secretion. Molecular and cellular endocrinology. 2014;382(1):178-89.
452. Hwang SL, Kwon O, Kim SG, Lee IK, Kim YD. B-cell translocation gene 2 positively regulates GLP-1-stimulated insulin secretion via induction of PDX-1 in pancreatic beta-cells. Experimental & molecular medicine. 2013;45:e25.
453. Sabatini PV, Krentz NA, Zarrouki B, Westwell-Roper CY, Nian C, Uy RA, et al. Npas4 is a novel activity-regulated cytoprotective factor in pancreatic beta-cells. Diabetes. 2013;62(8):2808-20.
454. Briand O, Helleboid-Chapman A, Ploton M, Hennuyer N, Carpentier R, Pattou F, et al. The nuclear orphan receptor Nur77 is a lipotoxicity sensor regulating glucose-induced insulin secretion in pancreatic beta-cells. Molecular endocrinology (Baltimore, Md). 2012;26(3):399-413.
455. Pessin JE, Saltiel AR. Signaling pathways in insulin action: molecular targets of insulin resistance. The Journal of clinical investigation. 2000;106(2):165-9.
456. Song HP, Zhang L, Dang YM, Yan H, Chu ZG, Huang YS. The phosphatidylinositol 3-kinase-Akt pathway protects cardiomyocytes from ischaemic and hypoxic apoptosis via mitochondrial function. Clinical and experimental pharmacology & physiology. 2010;37(5-6):598-604.
457. Sheu ML, Ho FM, Chao KF, Kuo ML, Liu SH. Activation of phosphoinositide 3-kinase in response to high glucose leads to regulation of reactive oxygen species-related nuclear factor-kappaB activation and cyclooxygenase-2 expression in mesangial cells. Molecular pharmacology. 2004;66(1):187-96.
458. Assmann A, Ueki K, Winnay JN, Kadowaki T, Kulkarni RN. Glucose effects on beta-cell growth and survival require activation of insulin receptors and insulin receptor substrate 2. Molecular and cellular biology. 2009;29(11):3219-28.
459. Cao J, Yang X, Liu YN, Suo ZW, Shi L, Zheng CR, et al. GABAergic disinhibition induced pain hypersensitivity by upregulating NMDA receptor functions in spinal dorsal horn. Neuropharmacology. 2011;60(6):921-9.

460. Gallo EF, Iadecola C. Neuronal nitric oxide contributes to neuroplasticity-associated protein expression through cGMP, protein kinase G, and extracellular signal-regulated kinase. The Journal of neuroscience : the official journal of the Society for Neuroscience. 2011;31(19):6947-55.
461. Rao A, Luo C, Hogan PG. Transcription factors of the NFAT family: regulation and function. Annual review of immunology. 1997;15:707-47.
462. Lamph WW, Dwarki VJ, Ofir R, Montminy M, Verma IM. Negative and positive regulation by transcription factor cAMP response element-binding protein is modulated by phosphorylation. Proceedings of the National Academy of Sciences of the United States of America. 1990;87(11):4320-4.
463. Bachir LK, Garrel G, Lozach A, Laverriere JN, Counis R. The rat pituitary promoter of the neuronal nitric oxide synthase gene contains an Sp1-, LIM homeodomain-dependent enhancer and a distinct bipartite gonadotropin-releasing hormone-responsive region. Endocrinology. 2003;144(9):3995-4007.
464. Tullai JW, Chen J, Schaffer ME, Kamenetsky E, Kasif S, Cooper GM. Glycogen synthase kinase-3 represses cyclic AMP response element-binding protein (CREB)-targeted immediate early genes in quiescent cells. The Journal of biological chemistry. 2007;282(13):9482-91.
465. Klemm DJ, Leitner JW, Watson P, Nesterova A, Reusch JE, Goalstone ML, et al. Insulin-induced adipocyte differentiation. Activation of CREB rescues adipogenesis from the arrest caused by inhibition of prenylation. The Journal of biological chemistry. 2001;276(30):28430-5.
466. Nakanishi M, Hata K, Nagayama T, Sakurai T, Nishisho T, Wakabayashi H, et al. Acid activation of Trpv1 leads to an up-regulation of calcitonin gene-related peptide expression in dorsal root ganglion neurons via the CaMK-CREB cascade: a potential mechanism of inflammatory pain. Molecular biology of the cell. 2010;21(15):2568-77.
467. Benbrook DM, Jones NC. Heterodimer formation between CREB and JUN proteins. Oncogene. 1990;5(3):295-302.
468. David S, Kalb RG. Serum/glucocorticoid-inducible kinase can phosphorylate the cyclic AMP response element binding protein, CREB. FEBS letters. 2005;579(6):1534-8.
469. Ciani E, Guidi S, Della Valle G, Perini G, Bartesaghi R, Contestabile A. Nitric oxide protects neuroblastoma cells from apoptosis induced by serum deprivation through cAMP-response element-binding protein (CREB) activation. The Journal of biological chemistry. 2002;277(51):49896-902.
470. Lee SY, Gong EY, Hong CY, Kim KH, Han JS, Ryu JC, et al. ROS inhibit the expression of testicular steroidogenic enzyme genes via the suppression of Nur77 transactivation. Free radical biology & medicine. 2009;47(11):1591-600.
471. Kinoshita I, Leaner V, Katabami M, Manzano RG, Dent P, Sabichi A, et al. Identification of cJun-responsive genes in Rat-1a cells using multiple techniques: increased expression of stathmin is necessary for cJun-mediated anchorage-independent growth. Oncogene. 2003;22(18):2710-22.
472. Hsu JC, Laz T, Mohn KL, Taub R. Identification of LRF-1, a leucine-zipper protein that is rapidly and highly induced in regenerating liver. Proceedings of the National Academy of Sciences of the United States of America. 1991;88(9):3511-5.
473. Leaner VD, Kinoshita I, Birrer MJ. AP-1 complexes containing cJun and JunB cause cellular transformation of Rat1a fibroblasts and share transcriptional targets. Oncogene. 2003;22(36):5619-29.
474. Katiyar S, Jiao X, Addya S, Ertel A, Covarrubias Y, Rose V, et al. Mammary gland selective excision of c-jun identifies its role in mRNA splicing. Cancer research. 2012;72(4):1023-34.
475. Pearen MA, Muscat GE. Minireview: Nuclear hormone receptor 4A signaling: implications for metabolic disease. Molecular endocrinology (Baltimore, Md). 2010;24(10):1891-903.
476. Castro-Obregon S, Rao RV, del Rio G, Chen SF, Poksay KS, Rabizadeh S, et al. Alternative, nonapoptotic programmed cell death: mediation by arrestin 2, ERK2, and Nur77. The Journal of biological chemistry. 2004;279(17):17543-53.
477. Dhillo WS, Small CJ, Jethwa PH, Russell SH, Gardiner JV, Bewick GA, et al. Paraventricular nucleus administration of calcitonin gene-related peptide inhibits food intake and stimulates the hypothalamo-pituitary-adrenal axis. Endocrinology. 2003;144(4):1420-5.
478. Wright KL, Ward SG. Interactions between phosphatidylinositol 3-kinase and nitric oxide: explaining the paradox. Molecular cell biology research communications : MCBRC. 2000;4(3):137-43.
479. Alderton WK, Cooper CE, Knowles RG. Nitric oxide synthases: structure, function and inhibition. The Biochemical journal. 2001;357(Pt 3):593-615.
480. Sheu FS, Mahoney CW, Seki K, Huang KP. Nitric oxide modification of rat brain neurogranin affects its phosphorylation by protein kinase C and affinity for calmodulin. The Journal of biological chemistry. 1996;271(37):22407-13.

481. Prichard L, Deloulme JC, Storm DR. Interactions between neurogranin and calmodulin in vivo. The Journal of biological chemistry. 1999;274(12):7689-94.
482. Renodon A, Boucher JL, Sari MA, Delaforge M, Ouazzani J, Mansuy D. Strong inhibition of neuronal nitric oxide synthase by the calmodulin antagonist and anti-estrogen drug tamoxifen. Biochemical pharmacology. 1997;54(10):1109-14.
483. Castellani V, De Angelis E, Kenwrick S, Rougon G. Cis and trans interactions of L1 with neuropilin-1 control axonal responses to semaphorin 3A. The EMBO journal. 2002;21(23):6348-57.
484. Adamczyk A, Kazmierczak A, Czapski GA, Strosznajder JB. Alpha-synuclein induced cell death in mouse hippocampal (HT22) cells is mediated by nitric oxide-dependent activation of caspase-3. FEBS letters. 2010;584(15):3504-8.
485. Hyun DH, Lee M, Hattori N, Kubo S, Mizuno Y, Halliwell B, et al. Effect of wild-type or mutant Parkin on oxidative damage, nitric oxide, antioxidant defenses, and the proteasome. The Journal of biological chemistry. 2002;277(32):28572-7.
486. Olson AL, Pessin JE. Regulation of c-fos expression in adipose and muscle tissue of diabetic rats. Endocrinology. 1994;134(1):271-6.
487. Misra UK, Pizzo SV. Activated alpha2-macroglobulin binding to human prostate cancer cells triggers insulin-like responses. The Journal of biological chemistry. 2015;290(15):9571-87.
488. Lang F, Strutz-Seebohm N, Seebohm G, Lang UE. Significance of SGK1 in the regulation of neuronal function. The Journal of physiology. 2010;588(Pt 18):3349-54.
489. Reusch JE, Hsieh P, Klemm D, Hoeffler J, Draznin B. Insulin inhibits dephosphorylation of adenosine 3',5'-monophosphate response element-binding protein/activating transcription factor-1: effect on nuclear phosphoserine phosphatase-2a. Endocrinology. 1994;135(6):2418-22.

List of abbreviations

AC, anterior commissure;

ACh, acetylcholine;

CC, corpus callosum;

CNR, control (saline) not running;

CR, control (saline) running;

DA, dopamine;

DL, dorsolateral striatum;

FW, forward strand;

GPe, globus pallidus external;

GPi, globus pallidus internal;

L-DOPA, levodopa;

MNR, MPTP but not running;

MR, MPTP and running;

MSNs, medium spiny neurons;

PD, Parkinson's disease;

PFC, prefrontal cortex;

PPN, pedunculopontine nucleus;

RV, reverse strand;

SN, substantia nigra;

SNpc, substantia nigra pars compacta;

SNpr, substantia nigra pars reticularis;

STN, subthalamic nucleus;

VDCC, voltage-dependent calcium channel;

VM, ventromedial striatum;

VTA, ventral tegmental area;
